# Micronutrient fortification of food and its impact on woman and child health: a systematic review

**DOI:** 10.1186/2046-4053-2-67

**Published:** 2013-08-23

**Authors:** Jai K Das, Rehana A Salam, Rohail Kumar, Zulfiqar A Bhutta

**Affiliations:** 1 Centre of Excellence in Women & Child Health, Aga Khan University, Karachi 74800, Pakistan

**Keywords:** Child, Food, Fortification, Nutrition, Supplementation, Women

## Abstract

**Background:**

Vitamins and minerals are essential for growth and metabolism. The World Health Organization estimates that more than 2 billion people are deficient in key vitamins and minerals. Groups most vulnerable to these micronutrient deficiencies are pregnant and lactating women and young children, given their increased demands. Food fortification is one of the strategies that has been used safely and effectively to prevent vitamin and mineral deficiencies.

**Methods:**

A comprehensive search was done to identify all available evidence for the impact of fortification interventions. Studies were included if food was fortified with a single, dual or multiple micronutrients and impact of fortification was analyzed on the health outcomes and relevant biochemical indicators of women and children. We performed a meta-analysis of outcomes using Review Manager Software version 5.1.

**Results:**

Our systematic review identified 201 studies that we reviewed for outcomes of relevance. Fortification for children showed significant impacts on increasing serum micronutrient concentrations. Hematologic markers also improved, including hemoglobin concentrations, which showed a significant rise when food was fortified with vitamin A, iron and multiple micronutrients. Fortification with zinc had no significant adverse impact on hemoglobin levels. Multiple micronutrient fortification showed non-significant impacts on height for age, weight for age and weight for height Z-scores, although they showed positive trends. The results for fortification in women showed that calcium and vitamin D fortification had significant impacts in the post-menopausal age group. Iron fortification led to a significant increase in serum ferritin and hemoglobin levels in women of reproductive age and pregnant women. Folate fortification significantly reduced the incidence of congenital abnormalities like neural tube defects without increasing the incidence of twinning. The number of studies pooled for zinc and multiple micronutrients for women were few, though the evidence suggested benefit. There was a dearth of evidence for the impact of fortification strategies on morbidity and mortality outcomes in women and children.

**Conclusion:**

Fortification is potentially an effective strategy but evidence from the developing world is scarce. Programs need to assess the direct impact of fortification on morbidity and mortality.

## Background

Vitamins and minerals are essential for growth and metabolism. The World Health Organization (WHO) estimates that more than 2 billion people are deficient in key vitamins and minerals, particularly vitamin A, iodine, iron and zinc [1]. Most of these affected populations are from developing countries where multiple micronutrient (MMN) deficiencies coexist.

The population groups most vulnerable to these micronutrient deficiencies are pregnant and lactating women and young children, given their increased demands [2,3]. According to recent WHO estimates, globally about 190 million preschool children and 19.1 million pregnant women are vitamin A deficient (that is, have serum retinol <0.70 μmol/l) [4], approximately 100 million women of reproductive age (WRA) have iodine deficiency [5], and an estimated 82% of pregnant women worldwide have inadequate zinc intakes to meet the normal needs of pregnancy [6]. Iron deficiency is widespread and globally about 1.62 billion people are anemic, with the highest prevalence among preschool children (47%) followed by pregnant women (42%) [7]. Suboptimal vitamin B6 and B12 status have also been observed in many developing countries [8]. These micronutrient deficiencies are also associated with increased incidence and severity of infectious illness and mortality from diarrhea, measles, malaria and pneumonia [9]. The consequences of micronutrient deficiencies are not limited to health parameters alone but have far-reaching effects on economies through secondary physical and mental disabilities and altered work productivity.

Several strategies have been employed to supplement micronutrients to women and children [10-13]. These include education, dietary modification, food rationing, supplementation and fortification. Food fortification is one of the strategies that has been used safely and effectively to prevent micronutrient deficiencies and has been practiced in developed countries for well over a century now. In the early 20th century, salt iodization began in Switzerland; vitamin A-fortified margarine was introduced in Denmark in 1918; and in the 1930s, vitamin A-fortified milk and iron and B complex flour was introduced in a number of developed countries. These fortification strategies are now almost universal in the developed world and increasingly deployed in many middle-income countries. Relatively few of these programs have been adequately evaluated to assess their impact on population health [14].

WHO categorizes food fortification strategies into three possible approaches: mass, targeted, and market driven [14]. Mass fortification involves foods that are widely consumed, such as wheat, salt, sugar; targeted approaches fortify foods consumed by specific age groups like infant complementary foods; and the market-driven approach is when a food manufacturer fortifies a specific brand for a particular consumer niche. Food vehicles commonly used can be grouped into three broad categories: staples (wheat, rice, oils), condiments (salt, soy sauce, sugar), and processed commercial foods (noodles, infant complementary foods, dairy products).

Food fortification is an attractive public health strategy and has the advantage of reaching wider at-risk population groups through existing food delivery systems, without requiring major changes in existing consumption patterns [13,15]. Compared with other interventions, food fortification may be cost-effective and, if fortified foods are regularly consumed, has the advantage of maintaining steady body stores [15].

There are a number of systemic reviews and meta-analyses analyzing the effect of food fortification on health outcomes. An extensive review of zinc fortification in children described an overall significant impact on serum zinc levels with no adverse effects [16]. Reviews on MMN fortification in children showed improved micronutrient status and reduced anemia prevalence [10,17]. A review of iron fortification in the general population also showed an increase in serum hemoglobin levels and decreased anemia prevalence [18]. Although iron, zinc and MMN fortification has been consistently analyzed, literature on vitamin A, iodine and vitamin D fortification in children has not been sufficiently assessed. Furthermore, iron and MMN fortification in women of different age groups has not been adequately evaluated. We therefore feel that the current evidence of certain micronutrients and population groups may not be sufficient for providing a way forward. Hence we undertook a systematic review of the current evidence to assess the effectiveness of food fortification with single micronutrients (iron, folic acid, vitamin A, vitamin D, iodine, zinc) as well as MMN when compared with no fortification on the health and nutrition of women and children.

### Conceptual framework

**Figure 1:**
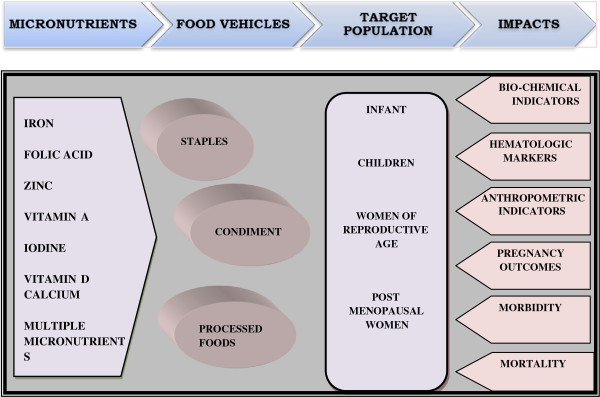
Conceptual framework.

We analyzed the impact of micronutrient fortification strategies - single, dual or multiple - on various outcomes guided by our conceptual framework (Figure 1). These micronutrients were administered through one of the three food vehicles (staples, condiments or processed foods) to reach the population targeted. For the scope of this review, we focused on *a priori* defined population groups of infants, children and adolescents under 18 years of age, WRA and post-menopausal women. The outcomes analyzed were broadly categorized into biochemical indicators, hematologic markers, anthropometric indicators, pregnancy outcomes, and relevant morbidity and mortality.

## Methods

### Search strategy

All available evidence for the impact of fortification interventions was systematically retrieved and analyzed. A comprehensive search was done for key words including Medical Subject Headings and free text terms for all the micronutrients included in this review. We searched MEDLINE, PubMed, POPLINE, Literatura Latino Americana em Ciências da Saúde, Cumulative Index to Nursing and Allied Health Literature, Cochrane Library, British Library for Development Studies at the International Development Statistics, WHO regional databases and the IDEAS database of unpublished working papers, Google and Google Scholar. Detailed manual searches were undertaken, including cross-references and bibliographies of available data and publications. Existing relevant reviews were used to identify additional sources of information. The search was extended to review the gray literature in non-indexed and non-electronic sources. The bibliographies of books with relevant sections were also searched manually to identify relevant reports and publications. We did not apply any language or date restriction and the date of last search was 1 November 2012.

### Inclusion and exclusion criteria

We included randomized controlled trials (RCTs), quasi- experimental and before-after studies. In addition, other less rigorous study designs like observational studies (cohort and case–control), program evaluations and descriptive studies were also reviewed to understand the context in which these interventions were implemented. Studies were included if food was fortified with a single, dual or multiple micronutrients and the impact of fortification was analyzed on the health outcomes and relevant biochemical indicators of women and children. We included studies with children and adolescents of all age groups - infants and preschool children (ages 2 to 5 years), school-going children (ages of above 5 years) and adolescents till 18 years of age. For women, we included studies on pre-pregnant women, WRA, and post-menopausal women. The control groups in the included trials either received unfortified foods or regular diets. Studies were included if they measured the relevant outcomes according to our conceptual framework described above and if the data for these outcomes were presented in a manner that could be included in the meta-analysis. Studies were not considered that focused on home fortification with micronutrient powders, food contents, intake levels, bioavailability, comparisons between different food vehicles or comparisons among compounds of the same micronutrient, comparisons between fortification and supplementation, bio-fortification and studies evaluating the sensory impacts of fortification.

### Study selection process

All the available studies underwent triage with standardized criteria for evaluating outputs from primary screening. Following an agreement on the search strategy, the abstracts and full texts were screened by two independent abstractors to identify studies adhering to the objectives. Any disagreements on selection of studies between the two primary abstractors were resolved by the third reviewer.

### Data extraction

After retrieval of the full texts of all the relevant studies, each study was double data abstracted into a standardized form, which included the following details.

#### General information

Title, author, year of publication, country of intervention, study design.

#### Intervention

Study duration, characteristics of intervention and comparison groups.

#### Fortification

Micronutrient fortified, food vehicle used, fortificant compound and its concentration in the food.

#### Key outcome measures

Outcome measures were identified and evaluated separately for the various micronutrients. Relevant biochemical indicators included serum micronutrient levels and hematologic markers (anemia, iron deficiency anemia, hemoglobin). Anthropometric indicators were stunting; wasting; underweight; and changes in Z-scores for height for age (HAZ), weight for age (WAZ) and weight for height (WHZ). Pregnancy outcomes included twinning and congenital abnormalities. Morbidity outcomes included diarrhea, pneumonia, malaria, urinary tract infections (UTI), fever and mortality (Table 1). These were reported as means, standard deviations, number of events or other usable outcomes that could be pooled. Where information was missing or incomplete, the authors were contacted for clarification and access to data. In cases where it was not possible, the outcome was not considered for further analysis.

**Table 1:** Outcomes for various micronutrients analyzed

**Micronutrient**	**Outcomes analyzed**
**Zinc**	Serum zinc concentration, height velocity, alkaline phosphatase, hemoglobin and serum copper concentration
**Folate**	Serum folate concentration, red blood cell folate concentration, folate deficiency, twinning, incidence of neural tube defects, anencephaly and spina bifida
**Vitamin A**	Serum vitamin A concentration, vitamin A deficiency and hemoglobin
**Vitamin D/calcium**	Serum 25-hydroxy vitamin D_3_ concentration, alkaline phosphatase and serum parathyroid hormone
**Iodine**	Serum thyroxin levels, prevalence of hypothyroidism and urinary iodine concentration
**Iron**	Serum ferritin, serum transferrin, hemoglobin and anemia
**Multiple micronutrients**	Serum ferritin, serum zinc, serum retinol, vitamin A deficiency, iron deficiency anemia, hemoglobin levels, height for age Z-scores, weight for age Z-scores, weight for height Z-scores and morbidity.

### Data analysis

We performed meta-analyses of all outcomes using Review Manager Software version 5.1. For dichotomous data, we presented results as summary risk ratio (RR) and odds ratio (OR) and their respective 95% confidence intervals (CI). For continuous data, we used the standard mean difference (SMD) if outcomes were comparable. We performed separate meta-analyses for RCTs or quasi experimental and before-after studies to maintain the quality of evidence. Results of before-after meta-analyses were only reported if there were no RCTs or quasi-experimental studies in a particular category of micronutrient fortification. For analyzing and pooling cluster randomized trial data, the entire cluster was used as the unit of randomization and the analysis was adjusted for design. The data of cluster-randomized trials were incorporated using a generic inverse variance method in which logarithms of RR estimates were used along with the standard error of the logarithms of RR estimates. Subgroup analyses were performed according to the different age groups, countries, population characteristics, type of food fortified and the duration of intervention. For the outcomes where only medians were reported, medians were converted to approximate of the means and then pooled for analysis [19].

The level of attrition was noted for each study and its impact on the overall assessment of treatment effect explored by using sensitivity analysis. Heterogeneity between trials was assessed using the I-squared statistic (I^2^ >30) and a *p*-value <0.1 (on chi-square) and by visual inspection of forest plots, and a random effect model was used.

### Risk of bias and quality assessment of evidence

We used the Cochrane Collaboration’s tool for assessing risk of bias for the included RCTs and quasi-experimental studies [20]. It is a two-part tool addressing the seven specific domains, namely sequence generation, allocation concealment, blinding of participants and personnel, blinding of outcome assessment, incomplete outcome data, selective outcome reporting, and ‘other issues’. Within each entry, the first part of the tool describes what was reported to have happened in the study in sufficient detail to support a judgment about the risk of bias. The second part of the tool assigns a judgment relating to the risk of bias for that entry. This is achieved by assigning a judgment of ‘low risk’, ‘high risk’ or ‘unclear risk’.

We summarized the evidence by outcome, including qualitative assessments of study quality and quantitative measures, according to the standard guidelines. For each outcome, the quality of the evidence was assessed independently by two review authors using the Grading of Recommendations Assessment, Development and Evaluation (GRADE) approach, which involves consideration of within-study risk of bias (methodological quality), directness of evidence, heterogeneity, precision of effect estimates, and risk of publication bias. A grade of ‘high’, ‘moderate’, ‘low’ and ‘very low’ was used for grading the overall evidence indicating the strength of an effect on specific health outcome [21-24].

## Results

**Figure 2:**
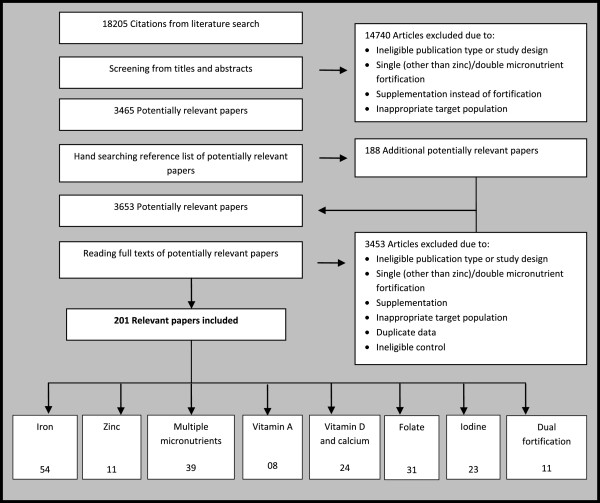
Summary of search strategy.

A total of 201 studies were identified for inclusion in this review. Of these, 121 [25-145] studies were on infants and children and 79 [146-224] were on women, while one study had both women and children as their study population [33] (Figure 2). Furthermore 125 [25-28,30,32-39,41-44,47,48,51,53],[54,56,59,60,62,63,65-73,75-77],[81-83,85,86,88-90,92,93,95-104,107-109],[111-117,119-127,129,130,132-136,139-144,223,225-228] of these trials were RCTs; the rest were quasi-experimental [46,55,61,74,78,87,91] and before-after studies [29,31,40,45,49,50,52,57],[58,79,80,84,94,95,105,131],[137,138,149,150,153,157,159-169,172],[174,177,181,183-186,189-191,193,195-200,202],[205-211,213,219-222,224]. Various micronutrients were used as fortificants in these studies including iron, zinc, vitamin A, folate, iodine, calcium and vitamin D alone and in combination. Food vehicles chosen varied for each micronutrient fortification and are described in detail in the relevant section. For the 125 RCTs included in this review, randomization and allocation concealment were adequate in 41 of the studies [33-37,56,60,63,65,68,69,72],[75,77,83,88,92,97,99,101],[103,107,108,112,113,115,116,122],[125,127,132,135,139,140,142,143],[146,147,188,214,217], information regarding attrition rates was adequately discussed with reasons in 110 studies [33-39,41-44,47,48,51,53,54,56],[59,60,62,63,65,67-73,75-77,81-83],[85,86,88-90,92,93,96-104,107-109,111-117],[119-127,129,130,132-136,139-144,171,173,175],[176,178-180,182,187,188,192,194,201],[212,214-218,223] and blinding was described adequately in 62 studies [25,26,28,30,32,35,38,39],[47,48,51,53,54,56,59,60],[62,65,67-72,75-77,81,83,86,93],[96,99,100,103,108,109,113,116],[119,123-125,130,133,134,140-144,146,170],[171,179,180,182,187,192,194,201]. The studies provided insufficient information on selective reporting, which limited us from making any judgment. We analyzed the outcomes separately for women and children and the outcomes analyzed for each micronutrient fortification are outlined in Table 1. Definitions of various outcomes used are outlined in Table 2. The characteristics of the included studies and quality assessment of the evidence are summarized below and in Tables 3, 4, 5, 6, 7 and 8 and further details along with the forest plots are provided in Additional files 1, 2 and 3.

**Table 2:** Definitions of various micronutrient deficiencies

**Term**	**Definition**
Anemia^a^	Children 6 to 59 months of age: Hb less than 110 g/l
	Children 5 to 11 years of age: Hb less than 115 g/l
	Children 12 to 14 years of age: Hb less than 120 g/l
	Women 15 years and older: Hb less than 120 g/l
Vitamin A deficiency^b^	Plasma (serum) retinol concentration of less than 20 μg/dl
Zinc deficiency^c^	Serum zinc concentration of less than 10.7 μmol/L
Asymptomatic zinc deficiency	Serum zinc concentration of less than 10.7 μmol/L with the absence of clinical signs and symptoms.

^a^ Adapted from WHO VIMNIS (available from http://www.who.int/vmnis/indicators/haemoglobin/en/) [229].

^b^ Adapted from WHO. Global prevalence of vitamin A deficiency in populations at risk 1995–2005 [230].

^c^ Adapted from WHO. Trace elements in human nutrition and health. Geneva, Switzerland: WHO, 1996 [231] Hb, hemoglobin.

**Table 3:** Summary of results for iron fortification in children

**Outcome/quality of evidence**	**Combined effect**	**Population**		**Age groups**		**Country**		**Fortificant type**		**Food vehicle**		
		**General/healthy population (studies/ participants)**	**Deficient population**	**Infants**	**Preschool and school- going children**	**Low- and lower middle income countries**	**Upper middle- and higher-income countries**	**Ferrous sulfate**	**NaFeEDTA**	**Processed food**	**Staples**	**Condiments**
**Hemoglobin levels**	**SMD: 0.55 (95% CI: 0.34, 0.76)**	**SMD: 0.50 (95% CI: 0.28, 0.71)**	**SMD: 0.79 (95% CI: 0.19, 1.38)**	**SMD: 0.81 (95% CI: 0.31, 1.31)**	**SMD: 0.46 (95% CI: 0.24, 0.67)**	**SMD: 0.32 (95% CI: 0.14, 0.50)**	**SMD: 0.67 (95% CI: 0.36, 0.97)**	**SMD: 0.50 (95% CI: 0.14, 0.86)**	**SMD: 0.76 (95% CI: 0.06, 1.47)**	**SMD: 0.55 (95% CI: 0.29, 0.82)**	**SMD: 0.44 (95% CI: 0.10, 0.79)**	SMD: 1.14 (95% CI: -0.09, 2.37)
***Quality of evidence: Moderate***		25 studies 5,083 participants	6 studies 1,184 participants	12 studies 1,834 participants	16 studies 3,832 participants	10 studies 1,937 participants	20 studies 4,176 participants	9 studies 1,076 participants	6 studies 1,172 participants	18 studies 3,317 participants	8 studies 1,941 participants	3 studies 705 participants
**Serum ferritin levels**	**SMD: 0.91 (95% CI: 0.38, 1.44)**	**SMD: 0.63 (95% CI: 0.28, 0.98)**	**SMD: 1.37 (95% CI: 0.01, 2.74)**	**SMD: 0.63 (95% CI: 0.28, 0.98)**	**SMD: 1.37 (95% CI: 0.01, 2.74)**	**SMD: 1.37 (95% CI: 0.01, 2.74)**	**SMD: 0.68 (95% CI: 0.49, 0.87)**	**SMD: 0.84 (95% CI: 0.60, 1.09)**	SMD: 1.83 (95% CI: -0.08, 3.73)	**SMD: 0.66 (95% CI: 0.36, 0.97)**	**SMD: 0.48 (95% CI: 0.17, 0.78)**	**SMD: 2.80 (95% CI: 2.43, 3.17)**
***Quality of evidence: Moderate***		4 studies 952 participants	3 studies 555 participants	4 studies 952 participants	3 studies 555 participants	3 studies 555 participants	3 studies 547 participants	3 studies 539 participants	2 studies 385 participants	5 studies 1,097 participants	1 study 170 participants	1 study 240 participants
**Effect on anemia**	**RR: 0.55 (95% CI: 0.42, 0.72)**	**RR: 0.64 (95% CI: 0.50, 0.82)**	RR: 0.23 (95% CI: 0.04, 1.25)	**RR: 0.42 (95% CI: 0.24, 0.72)**	**RR: 0.60 (95% CI: 0.43, 0.84)**	RR: 0.91 (95% CI: 0.72, 1.14)	**RR: 0.41 (95% CI: 0.28, 0.61)**	**RR: 0.52 (95% CI: 0.34, 0.79)**	RR: 0.46 (95% CI: 0.17, 1.23)	**RR: 0.46 (95% CI: 0.28, 0.76)**	RR: 0.81 (95% CI: 0.60, 1.10)	RR: 0.12 (95% CI: 0.01, 2.16)
***Quality of evidence: Moderate***		15 studies 3,096 participants	3 studies 555 participants	6 studies 1,234 participants	10 studies 2,013 participants	6 studies 1,171 participants	11 studies 2,396 participants	4 studies 630 participants	4 studies 800 participants	10 studies 1,617 participants	5 studies 1,312 participants	2 studies 568 participants

Estimates in **bold** show significant impacts. CI, confidence interval; NaFeEDTA, sodium iron ethylenediaminetetraacetate RR: relative risk; SMD: standard mean difference.

**Table 4:** Summary of results for zinc fortification in children

**Outcome/** **quality of evidence**	**Combined effect**	**Age groups**						**Zinc compound used for fortification**			**Food vehicle**		**Duration**	
		**Newborns with very low birth weight**	**Full term healthy infants**	**Infants at risk of stunting**	**Malnourished infants**	**School-going children**	**School-going children with asymptomatic zinc deficiency**	**Zinc sulfate**	**Zinc chloride**	**Zinc acetate**	**Milk products**	**Cereal products**	**Less than 6 months**	**6 months or more**
**Serum zinc levels**	**SMD: 1.28 (95% CI: 0.56, 2.01)**	SMD: 0.51 (95% CI: -0.32, 1.34)	SMD: 3.49 (95% CI: -0.36, 7.33)	SMD: 0.09 (95% CI: -0.24, 0.41)	SMD: 0.12 (95% CI: -0.55, 0.78)	SMD: 1.12 (95% CI: -0.21, 2.46)	**SMD: 2.77 (95% CI: 1.43, 4.11)**	No studies identified	No studies identified	No studies identified	**SMD: 1.46 (95% CI: 0.26, 2.66)**	**SMD: 1.16 (95% CI: 0.08, 2.24)**	**SMD: 1.48 (95% CI: 0.57, 2.39)**	**SMD: 1.51 (95% CI: 0.13, 2.89)**
***Quality of evidence: Moderate***		2 studies 64 participants	2 studies 71 infants	1 study 147 infants	1 study 35 infants	2 studies 347 participants	1 study 19 children				5 studies 176 participants	4 studies 513 participants	4 studies 381 participants	4 studies 155 participants
**Hemoglobin level**	SMD: -0.11 (95% CI: -0.52, 0.31)	No studies identified	No studies identified	No studies identified f	No studies identified	No studies identified	No studies identified	SMD: 0.00 (95% CI: -0.67, 0.67)	SMD: -0.39 (95% CI: -1.03, 0.24)	SMD: 0.28 (95% CI: -0.62, 1.19)	SMD: -0.21 (95% CI: -0.67, 0.25)	SMD: 0.28 (95% CI: -0.62, 1.19)	No studies identified	No studies identified
***Quality of evidence: Low***								1 study 34 children	1 study 39 children	1 study 19 children	2 studies 73 participants	1 study 19 participants		
**Serum copper levels**	SMD: 0.57 (95% CI: -0.91, 2.06)	No studies identified	SMD: 0.75 (95% CI: -1.65, 3.16)	No studies identified	SMD: 0.21 (95% CI: -0.46, 0.87)	No studies identified	No studies identified	No studies identified	No studies identified	No studies identified	SMD: 0.57 (95% CI: -0.91, 2.06)	SMD: -0.61 (95% CI: -1.54, 0.31)	No studies identified	No studies identified
***Quality of evidence: Very low***			3 studies 126 infants		1 study 35 infants						3 studies 142 participants	1 study 19 participants		
**Serum alkaline phosphatase levels**	SMD: 0.94 (95% CI: -0.29, 2.17)	No studies identified	SMD: 0.68 (95% CI: -0.90, 2.25)	No studies identified	No studies identified	No studies identified	**SMD: 1.56 (95% CI: 0.50, 2.62)**	No studies identified	No studies identified	No studies identified	SMD: 0.68 (95% CI: -0.90, 2.25)	**SMD: 1.56 (95% CI: 0.50, 2.62)**	No studies identified	No studies identified
***Quality of evidence: Low***			2 studies 100 infants				1 study 19 children				2 studies 100 participants	1 study 19 participants		
**Weight gain**	SMD: 0.50 (95% CI: -0.12, 1.11)	SMD: 0.33 (95% CI: -0.33, 0.99)	SMD: 1.48 (95% CI: -1.49, 4.45)	SMD: 0.00 (95% CI: -0.30, 0.30)	SMD: -0.10 (95% CI: -0.73, 0.52)	SMD: 0.25 (95% CI: -0.17, 0.67)	No studies identified	No studies identified	No studies identified	No studies identified	SMD: 0.77 (95% CI: -0.43, 1.96)	SMD: 0.08 (95% CI:-0.16, 0.33)	No studies identified	No studies identified
***Quality of evidence: Moderate***		1 study 36 infants	2 studies 80 infants	1 study 176 infants	1 study 39 infants	1 study 88 children					4 studies 155 participants	2 studies 264 participants		
**Height growth**	**SMD: 0.52 (95% CI: 0.01, 1.04)**	**SMD: 0.70 (95% CI: 0.02, 1.37)**	SMD: -0.48 (95% CI: -2.45, 1.48)	SMD: 0.05 (95% CI: -0.25, 0.35)	SMD: 0.16 (95% CI: -0.47, 0.79)	SMD: 0.31 (95% CI: -0.11, 0.74)	No studies identified	No studies identified	No studies identified	No studies identified	SMD: -0.09 (95% CI: -1.17, 0.99)	SMD: 0.14 (95% CI: -0.11, 0.38)	No studies identified	No studies identified
***Quality of evidence: Moderate***		1 study 36 infants	2 studies 80 infants	1 study 176 infants	1 study 39 infants	1 study 88 children					5 studies 187 participants	2 studies 264 participants		

CI, confidence interval; SMD: Standard mean difference. Estimates in **bold** show significant impacts.

**Table 5:** Summary of results for iodine, Vitamin A and calcium/vitamin D fortification in children

		**Iodine fortification**		
**Outcome/quality of evidence**	**Combined effect**			
**Serum thyroxin levels**	SMD: 0.45 (95% CI: -1.15, 2.06)	2 studies 1,131 participants reported this outcome. The studies were conducted in Africa on school-going children.		
***Quality of evidence: Low***				
**Urinary iodine concentrations**	**SMD: 6.39 (95% CI: 2.69, 10.08)**	2 studies 1,016 participants reported this outcome. The studies were conducted in Africa on school-going children.		
***Quality of evidence: Moderate***				
		**Vitamin A fortification**		
**Outcome/quality of evidence**	**Combined effect**			
**Hemoglobin levels**	**SMD: 0.48 (95% CI: 0.07, 0.89)**	2 studies with1,538 participants reported the outcome. The study by Zhang *et al.*[132] used four different comparison groups. The first group received low amounts of vitamin A-fortified biscuits for 9 months daily. The second group received high amount of vitamin A-fortified biscuits for 3 months daily. The third group received very high amounts of vitamin A-fortified biscuits for 3 months weekly. The standard mean differences for the three groups were 0.73 (95% CI: 0.42, 1.04), 0.62 (95% CI: 0.38, 0.85) and 0.63 (95% CI: 0.38, 0.88) respectively.		
***Quality of evidence: Low***				
**Serum vitamin A concentration**	**SMD: 0.61 (95% CI: 0.39, 0.83)**	3 studies with 2,362 participants reported the outcome. The study by Zhang *et al.*[132] used four different comparison groups as explained in comments for hemoglobin levels. The standard mean differences for the three groups were 0.52 (95% CI: 0.21, 0.82), 0.73 (95% CI: 0.49, 0.97) and 0.44 (95% CI: 0.22, 0.66).		
***Quality of evidence: Low***				
**Vitamin A deficiency**	RR: 0.39 (95% CI: 0.09, 1.74)	2 studies with 1,465 participants reported the outcome. The study by Zhang *et al.*[132] showed positive impacts in reducing vitamin A deficiency whereas the study by Solon *et al.*[110] showed negative impacts. The latter used monosodium glutamate for vitamin A fortification and the quantity of vitamin A used was much less than that used by Zhang *et al*.		
***Quality of evidence: Moderate***				
**Calcium and vitamin D fortification**				
**Outcome/quality of evidence**	**Combined effect**	**Calcium only**	**Vitamin D only**	**Calcium and vitamin D**
**Serum parathyroid hormone levels**	**SMD: -0.40 (95% CI: -0.56, -0.24)**	**SMD: -0.28 (95% CI: -0.50, -0.06)**	No studies identified	**SMD: -0.52 (95% CI: -0.74, -0.29)**
***Quality of evidence: Low***		2 studies 317 participants		2 studies 327 participants
**Serum vitamin D levels**	**SMD: 1.23 (95% CI: 0.35, 2.11)**	SMD: -0.15 (95% CI: -0.41, 0.10)	**SMD: 1.76 (95% CI: 0.37, 3.15)**	**SMD: 1.58 (95% CI: 1.28, 1.87)**
***Quality of evidence: Moderate***		1 study 233 participants	2 studies 651 participants	1 study 235 participants
**Serum calcium levels**	**SMD: -0.40 (95% CI: -0.59, -0.20)**	**SMD: -0.30 (95% CI: -0.56, -0.04)**	No studies identified	**SMD: -0.50 (95% CI: -0.76, -0.24)**
***Quality of evidence: Low***		1 study 231 participants		1 study 235 participants

Estimates in **bold** show significant impacts. CI, confidence intervals; RR: relative risk; SMD: standard mean difference.

**Table 6:** Summary of results for multiple micronutrient fortification in children

**Outcome/quality of evidence**	**Combined effect**			**Age groups**		**Country**		**Number of micronutrients used**			**Duration of fortification**		
		**General/healthy population**	**Deficient population**	**Infants**	**Preschool and school-going children**	**Low- and lower middle-income countries**	**Upper middle- and higher-income countries**	**5 or less**	**6 to 12**	**More than 12**	**Less than 6 months**	**6 to 11 months**	**12 months or more**
**Hemoglobin levels**	**SMD: 0.75 (95% CI: 0.41, 1.08)**	**SMD: 0.72 (95% CI: 0.37, 1.07)**	**SMD: 1.06 (95% CI: 0.75, 1.38)**	**SMD: 1.05 (95% CI: 0.48, 1.63)**	**SMD: 0.45 (95% CI: 0.12, 0.79**)	**SMD: 0.50 (95% CI: 0.21, 0.78)**	**SMD: 1.25 (95% CI: 0.45, 2.06)**	SMD: 1.50 (95% CI: -0.23, 3.23)	**SMD: 0.51 (95% CI: 0.24, 0.78)**	**SMD: 0.71 (95% CI: 0.32, 1.09)**	SMD: 0.07 (95% CI: -0.17, 0.31)	**SMD: 1.05 (95% CI: 0.55, 1.56)**	**SMD: 0.52 (95% CI: 0.07, 0.98)**
***Quality of evidence: Moderate***		13 studies 2,876 participants	1 study 175 participants	7 studies 1,508 participants	7 studies 1,543 participants	8 studies 1,769 participants	6 studies 1,282 participants	4 studies 747 participants	6 studies 1,449 participants	4 studies 855 participants	2 studies 310 participants	8 studies 2,025 participants	4 studies 716 participants
**Serum ferritin levels**	**SMD: 0.37 [0.13, 0.62**)	**SMD: 0.37 (95% CI: 0.13, 0.62)**	No studies identified	**SMD: 0.43 (95% CI: 0.17, 0.68)**	SMD: 0.06 (95% CI: -0.17, 0.29)	**SMD: 0.47 (95% CI: 0.14, 0.80)**	SMD: 0.18 (95% CI: -0.03, 0.40)	SMD: 0.45 (95% CI: 0.00, 0.90)	**SMD: 0.62 (95% CI: 0.18, 1.06)**	SMD: 0.17 (95% CI: -0.16, 0.50)	SMD: 0.45 (95% CI: 0.00, 0.90)	**SMD: 0.48 (95% CI: 0.07, 0.89)**	SMD: 0.20 (95% CI: -0.23, 0.64)
***Quality of evidence: Low***		7 studies 1,848 participants		6 studies 1,547 participants	1 study 301 participants	4 studies 1,380 participants	3 studies 468 participants	1 study 79 participants	2 studies 748 participants	4 studies 1,021 participants	1 study 79 participants	3 studies 1,049 participants	3 studies 7,20 participants
**Serum zinc levels**	SMD: 0.08 [−0.02, 0.19)	SMD: 0.08 (95% CI: -0.02, 0.19)	No studies identified	SMD: 0.04 (95% CI: -0.10, 0.17)	**SMD: 0.17 (95% CI: 0.04, 0.30)**	**SMD: 0.12 (95% CI: 0.01, 0.23)**	SMD: -0.02 (95% CI: -0.26, 0.23)	SMD: -0.12 (95% CI: -0.75, 0.51)	SMD: 0.13 (95% CI: -0.02, 0.29)	SMD: 0.06 (95% CI: -0.05, 0.17)	**SMD: 0.31 (95% CI: 0.06, 0.57)**	SMD: 0.06 (95% CI: -0.09, 0.21)	SMD: 0.02 (95% CI: -0.11, 0.15)
***Quality of evidence: Moderate***		11 studies 2,972 participants		7 studies 1,780 participants	4 studies 1,192 participants	7 studies 1,960 participants	4 studies 1,012 participants	2 studies 232 participants	4 studies 1,820 participants	5 studies 920 participants	1 study 223 children	6 studies 1,128 participants	4 studies 1,221 participants
**Serum retinol levels**	SMD: -0.05 [−0.23, 0.13)	SMD: 0.05 (95% CI: -0.23, 0.13)	No studies identified	SMD: 0.04 (95% CI: -0.22, 0.30)	SMD: -0.21 (95% CI: -0.34, -0.07)	SMD: -0.06 (95% CI: -0.25, 0.14)	SMD: 0.01 (95% CI: -0.63, 0.64)	SMD: -0.28 (95% CI: -0.45, -0.11)	SMD: -0.03 (95% CI: -0.33, 0.27)	SMD: 0.02 (95% CI: -0.27, 0.31)	No studies identified	SMD: -0.08 (95% CI: -0.29, 0.13)	SMD: 0.03 (95% CI: -0.37, 0.43)
***Quality of evidence: Low***		8 studies 1,927 participants		4 studies 964 participants	4 studies 963 participants	6 studies 1,320 participants	to 2 studies 607 participants	2 studies 533 participants	5 studies 878 participants	3 studies 516 participants		3 studies 1,676 participants	3 studies 251 participants
**Effect on anemia**	**RR: 0.55 (95% CI: 0.42, 0.71)**	**RR: 0.59 (95% CI: 0.46, 0.75)**	**RR: 0.20 (95% CI: 0.10, 0.40)**	**RR: 0.59 (95% CI: 0.50, 0.70)**	**RR: 0.45 (95% CI: 0.22, 0.89**)	**RR: 0.50 (95% CI: 0.37, 0.67)**	RR: 0.69 (95% CI: 0.41, 1.15)	No studies identified	No studies identified	No studies identified	RR: 0.77 (95% CI: 0.43, 1.36)	**RR: 0.54 (95% CI: 0.36, 0.83)**	**RR: 0.50 (95% CI: 0.33, 0.77)**
***Quality of evidence: Low***		9 studies 2,880 participants	1 study 175 participants	5 studies 1809 participants	5 studies 1246 participants	7 studies 2,229 participants	3 studies 826 participants				1 study 223 children	4 studies 1,097 participants	5 studies 1,726 participants
**Effect on vitamin A deficiency**	RR: 0.90 (95% CI: 0.76, 1.06)	RR: 0.90 (95% CI: 0.76, 1.06)	No studies identified	RR: 0.85 (95% CI: 0.69, 1.03)	RR: 1.02 (95% CI: 0.76, 1.38)	RR: 0.88 (95% CI: 0.73, 1.07)	RR: 0.95 (95% CI: 0.66, 1.36)	No studies identified	No studies identified	No studies identified	No studies identified	RR: 0.85 (95% CI: 0.71, 1.03)	RR: 1.14 (95% CI: 0.76, 1.71)
***Quality of evidence: Low***		6 studies 2,036 participants		3 studies 687 participants	3 studies 1349 participants	4 studies 1,391 participants	2 studies 645 participants					3 studies 1,308 participants	3 studies 728 participants
**Height-for-age Z-score**	SMD: 0.13 (95% CI: -0.04, 0.29)	SMD: 0.12 (95% CI: -0.09, 0.32)	SMD: 0.22 (95% CI: -0.04, 0.47)	**SMD: 0.26 (95% CI: 0.12, 0.40)**	SMD: -0.01 (95% CI: -0.21, 0.20)	SMD: 0.09 (95% CI: -0.17, 0.34)	**SMD: 0.18 (95% CI: 0.02, 0.34)**	SMD: -0.00 (95% CI: -0.19, 0.18)	SMD: 0.11 (95% CI: -0.12, 0.34)	SMD: 0.18 (95% CI: -0.07, 0.42)	SMD: 0.01 (95% CI: -0.13, 0.15)	SMD: 0.11 (95% CI: -0.12, 0.34)	**SMD: 0.39 (95% CI: 0.24, 0.55)**
***Quality of evidence: Low***		6 studies 1,853 participants	2 studies 245 participants	5 studies 1,083 participants	3 studies 1,009 participants	5 studies 1,485 participants	3 studies 607 participants	2 studies 624 participants	1 study 288 participants	5 studies 1,180 participants	5 studies 1,170 participants	1 study 288 participants	2 studies 634 participants
**Weight-for-age Z-score**	SMD: -0.12 (95% CI: -0.43, 0.20)	SMD: 0.05 (95% CI: -0.09, 0.18)	SMD: 0.18 (95% CI: -0.14, 0.49)	SMD: -0.28 (95% CI: -0.85, 0.29)	SMD: 0.09 (95% CI: -0.16, 0.33)	SMD: 0.10 (95% CI: -0.13, 0.32)	SMD: -0.48 (95% CI: -1.45, 0.49)	SMD: -0.02 (95% CI: -0.28, 0.24)	SMD: -0.03 (95% CI: -0.26, 0.20)	SMD: -0.22 (95% CI: -0.76, 0.33)	SMD: -0.25 (95% CI: -0.90, 0.41)	SMD: 0.06 (95% CI: -0.09, 0.21)	SMD: -0.68 (95% CI: -2.79, 1.42)
***Quality of evidence: Low***		6 studies 1,853 participants	2 studies 245 participants	5 studies 1083 participants	3 studies 1,009 participants	5 studies 1,485 participants	3 studies 607 participants	2 studies 624 participants	1 study 288 participants	5 studies 1,180 participants	5 studies 1,170 participants	1 study 288 participants	2 studies 634 participants
**Weight-for-height Z-score**	SMD: -0.11 (95% CI: -0.40, 0.17)	SMD: -0.17 (95% CI: -0.56, 0.22)	SMD: -0.56 (95% CI: -1.42, 0.31)	SMD: 0.08 (95% CI: -0.06, 0.21)	SMD: -0.39 (95% CI: -1.06, 0.28)	SMD: 0.07 (95% CI: -0.07, 0.20)	SMD: -0.40 (95% CI: -1.17, 0.38)	SMD: -0.01 (95% CI: -0.29, 0.26)	SMD: -0.14 (95% CI: -0.37, 0.09)	SMD: -0.16 (95% CI: -0.66, 0.34)	SMD: -0.10 (95% CI: -0.75, 0.56)	SMD: -0.23 (95% CI: -0.64, 0.18)	**SMD: 0.18 (95% CI: 0.02, 0.34)**
***Quality of evidence: Low***		6 studies 1,853 participants	2 studies 245 participants	5 studies 1,083 participants	3 studies 1,009 participants	5 studies 1,485 participants	3 studies 607 participants	2 studies 624 participants	1 study 288 participants	5 studies 1,180 participants	5 studies 1,170 participants	1 study 288 participants	2 studies 634 participants

Estimates in **BOLD** show significant impacts. CI, confidence intervals; RR: relative risk; SMD: standard mean difference.

**Table 7:** Summary of results for iron, folate and calcium/vitamin D fortification in women

**Iron fortification**										
**Outcome/quality of evidence**	**Combined effect**	**Population**		**Country**		**Fortificant type**		**Food vehicle**		
		**General/healthy population**	**Deficient population**	**Low- and lower middle-income countries**	**Upper middle- and higher-income countries**	**Ferrous sulfate**	**NaFeEDTA**	**Processed food**	**Staples**	**Condiments**
**Hemoglobin levels**	**SMD: 0.62 (95% CI: 0.36, 0.89)**	**SMD: 0.66 (95% CI: 0.30, 1.02)**	**SMD: 0.59 (95% CI: 0.34, 0.83)**	SMD: 0.52 (95% CI: -0.12, 1.16)	**SMD: 0.66 (95% CI: 0.31, 1.00)**	**SMD: 0.44 (95% CI: 0.21, 0.67)**	**SMD: 0.48 (95% CI: 0.22, 0.73)**	**SMD: 0.50 (95% CI: 0.30, 0.70)**	SMD: 0.90 (95% CI: -0.10, 1.90)	**SMD: 0.45 (95% CI: 0.22, 0.68)**
***Quality of evidence: Moderate***		7 studies 2,819 participants	4 studies 404 participants	2 studies 524 participants	8 studies 2,588 participants	3 studies 310 participants	4 studies 1,588 participants	4 studies 398 participants	3 studies 1,211 participants	4 studies 1,614 participants
**Serum ferritin levels**	**SMD: 0.41 (95% CI: 0.23, 0.60)**	**SMD: 0.23 (95% CI: 0.08, 0.38)**	**SMD: 0.75 (95% CI: 0.47, 1.03)**	**SMD: 0.48 (95% CI: 0.31, 0.66)**	**SMD: 0.39 (95% CI: 0.17, 0.61)**	**SMD: 0.42 (95% CI: 0.05, 0.78)**	**SMD: 0.45 (95% CI: 0.21, 0.69)**	**SMD: 0.46 (95% CI: 0.10, 0.82)**	SMD: 0.17 (95% CI: -0.15, 0.49)	**SMD: 0.50 (95% CI: 0.33, 0.68)**
***Quality of evidence: Low***		6 studies 2,609 participants	4 studies 400 participants	2 studies 525 participants	8 studies 2,484 participants	1 study 117 participants	4 studies 1,559 participants	5 studies 1,390 participants	to 2 studies 993 participants	3 studies 626 participants
**Effect on anemia**	**RR: 0.68 (95% CI: 0.49, 0.93)**	RR: 0.65 (95% CI: 0.40, 1.04)	**RR: 0.74 (95% CI: 0.61, 0.90)**	**RR: 0.74 (95% CI: 0.61, 0.90)**	RR: 0.67 (95% CI: 0.39, 1.17)	**RR: 0.57 (95% CI: 0.34, 0.94)**	**RR: 0.74 (95% CI: 0.61, 0.90)**	RR: 0.51 (95% CI: 0.21, 1.25)	RR: 0.67 (95% CI: 0.39, 1.17)	**RR: 0.74 (95% CI: 0.61, 0.90)**
***Quality of evidence: Low***		3 studies 1,180 participants	1 study 136 participants	1 study 136 participants	2 studies 1,056 participants	2 studies 199 participants	1 study 136 participants	1 study 124 participants	2 studies 1,056 participants	1 study 136 participants
**Folate fortification**										
**Outcome/quality of evidence**	**Combined effect**	**Quantity**					**Duration**			
		**40 μg/100 g**			**More than 100 μg/100 g**		**Less than 1 year**		**Greater than 1 year**	
**Neural tube defects**	**RR: 0.57 (95% CI: 0.45, 0.73)**	**RR: 0.80 (95% CI: 0.75, 0.86)**			**RR: 0.55 (95% CI: 0.41, 0.75)**		**RR: 0.52 (95% CI: 0.28, 0.97)**		**RR: 0.60 (95% CI: 0.50, 0.72)**	
***Quality of evidence: Moderate***		3 studies			2 studies		3 studies		4 studies	
**Spina bifida**	**RR: 0.64 (95% CI: 0.57, 0.71)**	**RR: 0.72 (95% CI: 0.65, 0.80)**			**RR: 0.51 (95% CI: 0.41, 0.65)**		**RR: 0.72 (95% CI: 0.65, 0.80)**		**RR: 0.56 (95% CI: 0.49, 0.65)**	
***Quality of evidence: Moderate***		6 studies			3 studies		3 studies		7 studies	
**Anencephaly**	**RR: 0.76 (95% CI: 0.68, 0.85)**	**RR: 0.81 (95% CI: 0.74, 0.88)**			RR: 0.67 (95% CI: 0.43, 1.04)		**RR: 0.85 (95% CI: 0.79, 0.93)**		**RR: 0.70 (95% CI: 0.59, 0.83)**	
***Quality of evidence: Moderate***		6 studies			2 studies		3 studies		7 studies	
**Serum folate levels**	SMD: 1.38 (95% CI: -0.20, 2.95)	No studies identified			No studies identified		SMD: 1.65 (95% CI: -0.51, 3.82)		SMD: 0.45 (95% CI: -0.06, 0.97)	
***Quality of evidence: Low***							3 studies		2 studies	
**Iodine fortification**										
**Outcome/quality of evidence**		**Combined effect**								
**Effect on hypothyroidism**		**RR: 1.42 (95% CI: 1.11, 1.82)**								
***Quality of evidence: Low***		2 studies								
**Urinary iodine concentrations**		**SMD: 7.16 (95% CI: 1.00, 13.31)**								
***Quality of evidence: Moderate***		4 studies								
**Calcium and vitamin D fortification**										
**Outcome/quality of evidence**		**Women of reproductive age**					**Post-menopausal women**			
		**Vitamin D only**			**Calcium and vitamin D**		**Calcium only**		**Vitamin D only**	
**Serum parathyroid hormone levels**		SMD: 0.10 (95% CI: -0.28, 0.48)			SMD: -0.23 (95% CI: -0.78, 0.32)		No studies identified		No studies identified	
***Quality of evidence: Low***		2 studies 108 participants			1 study 52 participants					
**Serum vitamin D levels**		SMD: 0.26 (95% CI: -0.22, 0.75)			SMD: -2.50 (95% CI: -3.22, -1.78)		**SMD: 0.69 (95% CI: 0.38, 1.00)**		No studies identified	
***Quality of evidence: Moderate***		1 study 66 participants			1 study 55 participants		3 studies 228			

Estimates in **BOLD** show significant impacts. CI, confidence intervals; NaFeEDTA: sodium iron ethylenediaminetetraacetate; RR: relative risk; SMD: standard mean difference.

**Table 8:** Summary of results for multiple micronutrient fortification in women

**Outcome/quality of evidence**	**Combined effect**
**Hemoglobin levels**	**SMD: 0.31 (95% CI: 0.13, 0.48)**
***Quality of evidence: Moderate***	1 study 516 women
**Serum ferritin levels**	**SMD: 0.47 (95% CI: 0.36, 0.58)**
***Quality of evidence: Moderate***	2 studies 1,214 women
**Serum zinc levels**	**SMD: 0.50 (95% CI: 0.38, 0.61)**
***Quality of evidence: Moderate***	2 studies 1214 women
**Serum retinol levels**	**SMD: 0.47 (95% CI: 0.30, 0.65)**
***Quality of evidence: Moderate***	1 study 516 women
**Effect on anemia**	RR: 0.76 (95% CI: 0.48, 1.21)
***Quality of evidence: Moderate***	1 study 516 women

Estimates in **bold** show significant impacts. RR: relative risk; SMD: standard mean difference.

### Children

#### Iron fortification

A total of 41 [25,26,28,30-32,35,39,48,49],[54,56,57,65,70,77,80,81],[83,85,88,89,97,99,101,104],[106-108,112-114,116,120,124,126],[127,129,135,136,139] studies reported outcomes for impact of iron fortification. Four of the studies were before-after studies and the rest were RCTs. Nineteen of these studies had infants as their study population, fortifying formula milk, cow’s milk and complementary baby foods. Condiments such as curry powder, fish sauce and soy sauce were the vehicles used in a few studies targeting Asian populations. Drinking water, food bars, candies, noodles and fruit juices were also assessed as potential vehicles by various studies. Seven studies used sodium iron ethylenediaminetetraacetate (NaFeEDTA) and 15 used ferrous sulfate as the fortificant. Other forms of iron used were ferrous pyrophosphate, unspecified elemental iron and hydrogen-reduced iron. Twenty-two of the studies were carried out in upper middle income countries (UMIC) and higher income countries (HIC), and 18 were from lower middle income countries (LMIC) and lower income countries (LIC). Eight of the studies were carried out in populations with some form of iron deficiency or a comorbidity. A summary of results is presented in Table 3.

Pooled analyses from the RCTs demonstrated a significant increase in hemoglobin concentration (SMD: 0.55 (95% CI: 0.34, 0.76)), serum ferritin levels (SMD: 0.91 (95% CI: 0.38, 1.44)) and reduction in anemia (RR: 0.55 (95% CI: 0.42, 0.72)). A few studies reported the effect of iron fortification on cognition, but these could not be pooled together because the reported outcomes varied significantly.

Subgroup analysis according to the various age groups (infants, preschool and school going), countries (LIC/LMIC and UMIC/HIC) and baseline micronutrient status (deficient and normal populations) showed consistent findings for all the outcomes assessed. The impacts on serum ferritin levels and anemia were significant for all the three food vehicles, that is, processed food, condiments and staples, whereas the impact on hemoglobin levels was significant for processed food and staples only. The effect of NaFeEDTA when used as the fortificant was significant on hemoglobin levels and anemia but was non-significant on serum ferritin levels. The impacts of ferrous sulfate when used as the fortificant were significant for all the three outcomes assessed.

#### Zinc

Our search strategy identified 10 studies examining zinc fortification [34,41,44,53,59,78,91,100],[103,125], seven on infants, where infant formula feeds or milk were fortified, and three on school children, where porridge or bread was fortified [53,59,91]. Eight of the studies were RCTs and two were quasi-experimental designs. The studies varied in the duration of intervention ranging from 1 month to 12 months. Five studies employed zinc sulfate as the fortificant; other compounds used were zinc oxide, zinc chloride and zinc acetate. A summary of results is presented in Table 4.

Results from the RCTs showed a significant impact of zinc fortification on increasing serum zinc concentration (SMD: 1.28 (95% CI: 0.56, 2.01)), whereas non-significant impacts were observed for height velocity (SMD: 0.08 (95% CI: -0.53, 0.69)), weight gain (SMD: 0.50 (95% CI: -0.12, 1.11)), serum alkaline phosphatase (SMD: 0.94 (95% CI: -0.29, 2.17)), hemoglobin levels (SMD: -0.11, (95% CI: -0.52 to 0.31)) and serum copper levels (SMD: 0.22 (95% CI: -1.14 to 1.59)). Visual inspection of the forest plot for height velocity showed that the study by Salmenpera *et al.*[98] was the main outlier; after sensitivity analysis, it showed significant improvements (SMD: 0.52 (95% CI: 0.01, 1.04)).

Subgroup analysis showed a consistent positive effect on serum zinc levels for both milk- and cereal-based products, and for various durations of fortification (less than and more than six months). For the various age groups assessed, the effect was significant for only school-going children with an asymptomatic deficiency at baseline. The impact on weight gain was non-significant across all the various age groups while the effect on height gain was significant only for infants with very low birth weight.

#### Vitamin D and calcium

A total of seven RCTs, one quasi-experimental and two before-after studies were identified for vitamin D and calcium fortification [33,42,45,51,55,60,66,96],[133,134]. Milk was the preferred food vehicle and the amount of micronutrient used varied significantly among the studies. One study was on preterm infants while the rest were on children and adolescents ranging from 6 years to 18 years of age. A summary of results is presented in Table 5.

Analysis of the results from RCTs showed that fortification with vitamin D significantly increased serum concentration of 25-hydroxy-vitamin D_3_ (SMD: 1.23 (95% CI: 0.35, 2.11)) and reduced serum parathyroid hormone concentration (SMD: -0.40 (95% CI: -0.56, -0.24)).

#### Vitamin A

A total of three RCTs, one quasi-experimental and four before-after studies were identified for vitamin A fortification [29,36,79,84,95,110,111,132]. The studies used monosodium glutamate, sugar and flour as the fortificant. These studies spanned from 6 months to 2 years in duration. Five studies had children of ages ranging from 1 to 6 years, and three studies had a range of 6 to 16 years of age. A summary of results is presented in Table 5.

Analysis of the RCTs showed significant impacts on serum retinol concentration (SMD: 0.61 (95% CI: 0.39, 0.83)) and hemoglobin levels (SMD: 0.48 (95% CI: 0.07, 0.89)). A non-significant effect was shown on prevalence of vitamin A deficiency (RR: 0.39 (95% CI: 0.09, 1.74)).

#### Iodine

Iodine fortification was identified in seven studies [40,52,58,94,131,137,138]. All were before-after study designs evaluations of mass salt fortification programs.

The analysis showed a significant effect on median urinary iodine concentrations (SMD: 6.39 (95% CI: 2.69, 10.08)) whereas the effect on serum thyroxin levels was non-significant (SMD: 0.45 (95% CI: -1.15, 2.06)). A summary of results is presented in Table 5.

#### Dual fortification

Ten studies were identified to study dual fortification [27,90,109,123,128,140-144]. Nine of the studies were RCTs and one was a quasi-experimental design and included children ranging from 5 to 17 years. Iron and iodine were the most commonly used micronutrients with salt as the food vehicle. Our analysis showed a significant impact of iron and iodine fortification on hemoglobin concentration (SMD: 0.53 (95% CI: 0.18, 0.89)) and on reducing the prevalence of anemia (RR: 0.47 (95% CI: 0.34, 0.66)).

#### Multiple micronutrients

MMN fortification was considered when three or more micronutrients were fortified together. A total of 34 studies were identified that reported outcomes on children [36-38,43,46,47,50,61-64],[67-69,71-76,82,86,87,92,93,98],[102,105,115,117,118,121,122,130]. Three of these were before-after studies and the rest were RCTs and quasi-experimental studies. Eleven studies had infants as their study population, others included children up to 15 years of age. The duration of fortification ranged from 3 months to 4 years. All the studies employed targeted fortification as their fortification strategy. A summary of results is presented in Table 6.

Analysis of the RCTs showed a significant effect of MMN fortification on hemoglobin levels (SMD: 0.75 (95% CI: 0.41, 1.08)), serum ferritin concentration (SMD: 0.37 (95% CI: 0.13, 0.62)) and on reducing anemia prevalence (RR: 0.55 (95% CI: 0.42, 0.71)). The impacts were non-significant on serum zinc concentration, serum retinol concentration and vitamin A deficiency. MMN fortification also had a non-significant impact on HAZ, WAZ and WHZ with a SMD of 0.13 (95% CI: -0.04, 0.29), 0.11 (95% CI: -0.06, 0.28) and 0.04 (95% CI: -0.08, 0.16) respectively, although showing positive trends. Analysis showed a non-significant effect on overall morbidity (including fever, UTI, diarrhea and respiratory infections) with a RR of 1.19 (95% CI: 0.98, 1.44). There was a non-significant impact on diarrhea and respiratory infections but the incidence of UTIs was significantly reduced, although the evidence of UTIs was from a single study.

Subgroup analyses for various age groups demonstrated a significant impact on hemoglobin levels, serum ferritin levels, anemia prevalence and HAZ in infants. Significant effects were observed in hemoglobin levels, serum ferritin levels and anemia prevalence for preschool and school-going children. Subgroup analyses according to countries showed that MMN fortification in LMIC/LIC and UMIC/HIC had significant impacts on hemoglobin levels, whereas in LIC/LMIC, the significant impact was on serum ferritin levels and serum zinc levels.

### Women

#### Iron fortification

A total of 13 studies, including 11 RCTs, reported outcomes for the impact of iron fortification in women [35,151,154-156,173,175,178,203,204],[216,218,223]. Various fortificants, including NaFeEDTA, ferrous sulfate and ferrous pyrophosphate, were used. Ten of the studies were carried out in UMIC/HIC and three studies were from LIC/LMIC. Four of the studies were carried out in populations with some form of micronutrient deficiency or comorbidity. The most commonly reported outcomes included hemoglobin levels, serum ferritin levels and anemia. A summary of results is presented in Table 7.

Analysis of the RCTs showed a significant effect of iron fortification on hemoglobin concentration (SMD: 0.64 (95% CI: 0.30, 0.97)), serum ferritin levels (SMD: 0.41 (95% CI: 0.23, 0.60)) and on prevalence of anemia (RR: 0.68 (95% CI: 0.49, 0.93)).

Subgroup analyses showed that there was a significant impact on hemoglobin and ferritin levels for both the healthy and iron-deficient population, whereas the impact on anemia was only significant for iron-deficient population. Further subgroup analyses based on countries showed significant impacts of iron fortification on anemia and serum ferritin levels in LIC/LMIC and UMIC/HIC whereas hemoglobin was significantly affected only in UMIC/HIC. All different types of fortificants used showed positive impact on anemia prevalence and hemoglobin levels.

#### Folate

Thirty one before-after mass fortification studies were identified [149,150,153,157,159,164,168,169],[172,174,177,181,184-186,189,193,195-197],[199,200,202,205,206,209-211,221,222],[224]. Flour was used as the food vehicle with the level of fortification varying from 40 μg/100 g to 500 μg/100 g. Duration of intervention varied between 1 and 10 years. A summary of results is presented in Table 7. Although anencephaly and spina bifida are two specific forms of neural tube defects, some studies described results for neural tube defects without mentioning the specific type. Thus we have pooled anencephaly, spina bifida and neural tube defects separately as mentioned in studies to avoid any misinterpretation.

Our analysis showed that folate fortification had a significant impact in reducing neural tube defects (RR: 0.57 (95% CI: 0.45, 0.73)), spina bifida (RR: 0.64 (95% CI: 0.57, 0.71)) and anencephaly (RR: 0.80 (95% CI: 0.73, 0.87)). The impacts were non-significant for red blood cell folate levels (SMD: 1.26 (95% CI: -0.49, 3.01)), serum folate concentration (SMD: 1.38 (95% CI: -0.20, 2.95)) and twinning (RR: 1.06 (95% CI: 0.92, 1.22)).

Subgroup analyses showed that use of various concentration of folate (40 μg/100 g or more than 100 μg/100 g) had consistent significant impacts in reducing neural tube defects and spina bifida, whereas only 40 μg/100 g folate fortification was effective in reducing incidence of anencephaly. In a subgroup analysis based on duration of intervention of 1 year or more than 1 year, both had significant impacts in reducing neural tube defects, spina bifida and anencephaly.

#### Iodine

A total of 16 studies were identified investigating iodine fortification [160-163,165-167,183,190,191,198,207,208],[213,219,220]. All of these were before-after studies that used mass fortification as the strategy with salt being the most commonly used food vehicle.

The analysis showed that iodine fortification had a significant impact on median urinary iodine concentrations with a SMD of 7.16 (95% CI: 1.00, 13.31) and on the incidence of hypothyroidism with a RR of 1.42 (95% CI: 1.11, 1.82). The effect was non-significant on serum thyroxin levels with a SMD of 0.45 (95% CI: -1.15, 2.06).

#### Vitamin D and calcium

A total of 13 RCTs and one before-after study were identified for vitamin D and calcium fortification [146,147,158,170,171,179,180,182],[187,192,194,201,212,215]. The level of fortification varied significantly. Fortification durations varied from 2 weeks to 2 years. Reported outcomes included serum parathyroid hormone levels, serum vitamin D levels, serum calcium levels and bone mineral density. Target populations included WRA and post-menopausal women. A summary of results is presented in Table 7.

The analysis showed that vitamin D and calcium fortification in WRA had a non-significant impact on vitamin D_3_ levels with a SMD of −1.10 (95% CI: -3.81, 1.60) and serum parathyroid hormone levels with SMD of −0.01 (95% CI: -0.32, 0.30). For the post-menopausal women, pooled analysis showed significant impacts on serum concentration of 25-hydroxy-D_3_ with a SMD of 0.82 (95% CI: 0.30, 1.34) and on serum parathyroid hormone concentration with SMD of −2.53 (95% CI: -4.42, -0.65).

Results also indicated a significant impact of vitamin D and calcium fortification on reducing the serum levels of bone resorption markers: P1NP and CTx. Specifically, pooled analyses showed significantly reduced serum levels of P1NP (three studies; SMD of −3.36 (95% CI: -6.37, -0.35)) and CTx (four studies; SMD of −4.93 (95% CI: -7.78, -2.08)) in both WRA and post-menopausal women

#### Multiple micronutrients

Five RCTs were identified for the impact of MMN fortification on women [148,176,188,214,217]. Varying number (3 to 20) of micronutrients were used and the duration of intervention ranged from 6 months to 2 years. The study population included WRA, pregnant and post-menopausal women. A summary of results is presented in Table 8.

Analysis showed that MMN fortification significantly improved hemoglobin levels (SMD: 0.31 (95% CI: 0.13, 0.48)), serum ferritin (SMD: 0.47 (95% CI: 0.36, 0.58)), serum zinc (SMD: 0.50 (95% CI: 0.38, 0.61)) and serum retinol (SMD: 0.47 (95% CI: 0.30, 0.65)), and reduced anemia prevalence (RR: 0.76 (95% CI: 0.48, 1.21)). However, these findings should be interpreted with caution as only a few studies were pooled for each outcome.

## Discussion

Various strategies have been pursued globally to alleviate nutritional deficiencies including food programs, diet modification, supplementation and fortification. In this review, we have attempted to broadly quantify the impacts of fortification of various commodities with micronutrients, singly or in combination, and to compare benefits.

Our review of fortification strategies among children does show significant impacts on increasing respective serum micronutrient concentrations, which could indirectly be used to assess impact at population level. Zinc fortification led to a significant increase in serum zinc concentration, and the same was observed with vitamin A and vitamin D fortification on respective serum micronutrient concentrations. Fortification with iron or MMN fortification was associated with a significant increase in serum ferritin, whereas the effect of MMN fortification on serum retinol and serum zinc was non-significant. Other hematologic markers also improved following food fortification among children with vitamin A, iron and MMN, suggesting some conjoint benefits. Comparable to the findings by others [65], the observed lack of negative effects of zinc fortification on hemoglobin concentrations suggests that that the quantities of zinc present in fortified foods is not high enough to alter the absorption of iron. Although benefits of zinc fortification were seen on linear growth, MMN fortification was not associated with any significant benefits on HAZ, WAZ and WHZ.

Our analysis of the impact of fortification among women indicates that calcium and vitamin D fortification do not lead to an increase in serum vitamin D_3_ levels in WRA but has a significant effect in post-menopausal age groups. Iron fortification led to a significant increase in serum ferritin and hemoglobin levels in WRA and pregnant women. Folate fortification significantly reduced the incidence of congenital abnormalities like anencephaly, spinal bifida and neural tube defects. Similarly, fortification of salt with iodine decreased the incidence of hypothyroidism and led to higher median urinary iodine concentrations. This evidence suggests that mass fortification strategies can be extremely productive and beneficial for numerous health outcomes. The total number of studies pooled for zinc and MMN fortification in women were insufficient to draw firm conclusions.

Few studies in our review reported the direct impact of fortification on morbidity. However, as many supplementation trials have already proven the effects of improved micronutrient status on morbidity, it could be inferred that these improvements in micronutrient status, reflected in changes in serum concentrations, could also have beneficial effects on health outcomes including morbidity and mortality. However, this cannot be definitely concluded without large scale studies measuring the direct impact of fortification on such outcomes or direct comparisons of fortification and supplementation strategies.

Notwithstanding the conclusions above, we are aware that biochemical responses to specific micronutrient fortification strategies may differ depending on the underlying nutritional conditions of the individuals or populations, the micronutrient compound used for fortification, and the food vehicle chosen. To cater to these variations, which are inherent to this strategy, we undertook diverse subgroup analyses to confirm and account for the differences, where possible. For this, we divided the studies and reported separate estimates based on the various age groups targeted, baseline micronutrient status, compound and foods chosen, concentration of the micronutrient in the food, the duration of intervention, and whether the evidence was from HIC or LMIC. Our analyses suggest that the impact of food fortification were stronger in populations with nutritional deficiencies at baseline, as there was greater hematological impact from food fortification with iron and MMNs in marginalized at-risk populations. We also analyzed the impact of food fortification on infants alone and the results indicated that iron fortification was associated with a significant increase in serum ferritin and also a reduction in the incidence of anemia, although effects on hemoglobin concentrations were non-significant. Zinc fortification in infants raised serum zinc concentrations without any observed benefits on linear growth and weight gain. MMN fortification significantly improved hematological parameters and was also associated with significant gains in HAZ scores, whereas no beneficial effects were observed for WAZ and WHZ.

The choice of food and fortificant are critically important. The fortificant must be stable, have a long shelf life and should not alter the color, taste and appearance of food. The food matrix should not interfere with the absorption of the micronutrient, thereby affecting its bioavailability, for example through phytates. Apart from the scientific aspects, the choice of food should be such that it is readily available to the target population and the quantity consumed should be enough to match the dietary requirements. This is particularly true for children, who typically do not consume the same foods and staples. A variety of food vehicles have been chosen for fortification, broadly classified as staples, condiments and commercially processed foods. Our review shows that, overall, staples have been the primary choice as they are widely consumed by the population, whereas processed foods and cereals have been chosen when infants were the target population. Iodine fortification almost exclusively used salt as a traditional and proven method, folate fortification was commonly employed through grains or grain products, and milk has been the carrier of choice for vitamin D and calcium. Of the outcomes analyzed in this review, we could not conclude whether a specific compound was better for impacting individual micronutrient status, as consistent results were observed for iron sulfate and NaFeEDTA when used in iron fortification, and zinc sulfate, zinc chloride and zinc oxide when used in zinc fortification. However, we did not compare costs or the cost effectiveness of various approaches.

The process of fortification comes with significant costs, which have to be ultimately borne by the end consumer or the state. As the poor have limited purchasing power, funding agencies and public private partnerships are needed to provide costs subsidies and cost-sharing to ensure that the products reach those who need them most. Additional demand creation and social marketing may be necessary through campaigns to ensure adequate marketing pull factors and consumption.

Fortification promises to tackle a multitude of micronutrient deficiencies through a single intervention: a one-window solution. Although the results are encouraging, more is needed. The evidence from these subgroup analyses for different fortification vehicles and compounds was not conclusive, and suggests that further trials are needed with defined functional and biological outcomes. Most national programs analyzed were from developed countries and data from the developing world were relatively scarce. However, where available, the results showed comparable benefits. This scarcity of studies from developing countries is due to the fact that national fortification programs require large resources; robust scientific and research facilities are required to identify the ideal food, micronutrient compound and industrial support. The experience of commodities such as pre-fortified ready-to-use fortified foods indicates that global procurement and supply as well as local production is possible.

Our review has many limitations because we included a range of studies of varying sizes and scientific rigor. This was due to the fact that large scale fortification programs are, in general, before-after evaluations. Many studies used different foods and concentrations of micronutrients and the frequency of feeding/intake was not uniform. Limited information on confounding factors like age and nutritional status at the initiation of the intervention also limited inference of outcomes by age bands and nutrition categories, and the duration of the intervention period evaluated also varied across studies. There is limited evidence available for the direct impact of fortification on anthropometric measures, morbidity and mortality and these are essential to evaluate future benefits and effective strategies. We propose that further future trials are warranted, which should take into consideration the limitations identified in this review and draw upon this, so that we have a pool of appropriate studies with good quality study designs to impute the actual impacts of this promising strategy.

## Conclusion

Fortification, though promising, is not an answer to the global widespread nutritional deficiencies. With high burdens of diarrhea and enteropathy, widespread malabsorption may be a barrier to this strategy taking maximal effect. Integration of fortification and supplementation strategies together with other mother and child health and prevention programs may be the answer to address the widespread global under-nutrition and to ensure sustainable benefits. Community education and promotion campaigns should also be implemented parallel to the primary fortification programs to increase awareness, acceptability and equity. Fortification is potentially an effective strategy but evidence from the developing world is scarce and future programs also need to assess the direct impact of fortification on morbidity and mortality.

## Abbreviations

CI: Confidence interval; GRADE: Grading of Recommendations Assessment, Development and Evaluation; HAZ: Height for age Z-scores; HIC: High-income countries; LIC: Lower-income countries; LMIC: Lower middle-income countries; MMN: Multiple micronutrient; OR: Odds ratio; RCT: Randomized controlled trial; RR: Relative risk; SMD: Standard mean difference; UMIC: Upper middle-income countries; UTI: Urinary tract infections; WAZ: Weight for age Z-scores; WHZ: Weight for height Z-scores; WHO: World Health Organization; WRA: Women of reproductive age.

## Competing interests

The authors declare that they have no competing interests.

## Authors’ contributions

ZAB was responsible for designing and coordinating the review. JKD, RAS and RK were responsible for data collection, screening the search results, screening retrieved papers against inclusion criteria, appraising quality of papers, abstracting data from papers, entering data into RevMan, analysis and interpretation of data and writing the review. ZAB and JKD critically reviewed and modified the manuscript. All authors read and approved the final manuscript.

## Supplementary Material

Additional file 1 Characteristics of included studies. Click here for file

Additional file 2 Forest plots. Click here for file

Additional file 3 Summary of findings and quality of outcomes. Click here for file
