# Supplementary material for: Micronutrient fortification of food and its impact on woman and child health: a systematic review
Source: Syst Rev. 2013 Aug 23;2:67. doi: 10.1186/2046-4053-2-67 (PMC3765883; doi:10.1186/2046-4053-2-67)
Supplement: Additional file 1 — Characteristics of included studies. [file 2046-4053-2-67-S1.pdf]

Table S1 Study characteristics for iron fortification in children

Iron Fortification									
Author	Year	Country	Study design	Target population	Fortification strategy	Fortification compound and Carrier	Amount of micronutrient used for fortification	Duration of intervention period evaluated	Target population daily micronutrient Intake
Abizari[1]	2012	Ghana	RCT	5 -17 year old children	Targeted	Cowpea flour with NaFeEDTA	10 mg Fe/meal as NaFeEDTA	7 months	Not mentioned
Andang'o[2]	2007	Kenya	RCT	3-8 year old children	Targeted	NaFeEDTA or electrolytic iron fortified maize flour	Not mentioned	20 weeks	Not mentioned
Arcanjo[3]	2010	Brazil	RCT	2 to 5 year old children	Targeted	Iron fortified drinking water	10mg/L	16 weeks	Not mentioned
Daly Williams[4]	1996/1999	UK	RCT	Infants 6-8 months age	Targeted	Formula milk	12 mg/L	24 months	52 μg/100 mL unmodified cow's milk
Barbosa[5]	2012	Brazil	RCT	2 to 6 year old children	Targeted	Wheat rolls with microencapsulated iron	4mg/20g	24 weeks	Not mentioned
Beinner[6]	2005	Brazil	Before-After	Preschool children ages 6-59 months	Targeted	iron sulfatein Drinking water 5d/week	12 g elemental iron	8 months	Not mentioned
Beinner[7]	2010	Brazil	RCT	Anemic children 6-24 months age	Targeted	micronized ferric pyrophosphate in Rice	23.4g/kg	5 months	Not mentioned
Chen[8]	2005	China	RCT	Children above the age of 3 years	Targeted	NaFeEDTA in Soy sauce	296mg/L	18 months	Not mentioned
De Almeida[9]	2005	Brazil	RCT	Children at day care center (5 years and less)	Targeted	Water fortified with elemental iron	10mg elemental iron per LL	Six months	Not mentioned
Gill[10]	1997	UK and Ireland	RCT	6 months old infants	Targeted	Formula milk	12.3 mg/L	9 months	Not mentioned
Giorgini[11]	2001	Brazil	Before-After	Preschool children (12-72 months old)	Targeted	Sweet rolls	80mg/kg	6 months	Not mentioned
Haschke[12]	1988	Austria	RCT	Infants (less than 24 months old)	Targeted	Ferrous sulphate in Formula milk and Meat-containing baby food	20 mg/kg	8 months	Not mentioned
Huo[13]	2002	China	RCT	Anemic children 11-17 years of age	Targeted	NaFeEDTA in Soy sauce	Low: 1 g/L High 4g/L	3 months	Not mentioned
Iost[14]	1998	Brazil	Before-	Infants 6- 24 month old	Targeted	iron amino acid chelate in Butterfat	3mg/L	32 weeks	Not mentioned

			After			milk			
Thi Le[15]	2006	Vietnam	RCT	Anemic children of ages 5 to 12 years	Targeted	Instant noodles 5d/week	205.8 mg/kg	6 months	Not mentioned
Longfils[16]	2008	Cambodia	RCT	Students age 6-21 years	Targeted	NaFeEDTA and $FeSO_4$+citrate in fish sauce 6d/week	1.09 g/L	21 weeks	Not mentioned
Marsh[17]	1959	USA	RCT	Infants (less than 24 months)	Targeted	Formula milk with $FeSo_4$	12mg Fe in 32 ounces of milk	9 months	Not mentioned
Miglioranza[18]	2008	Brazil	Before-After	Children age 7-14 years	Mass	H2-reduced Fe in corn-flour	98 mg/kg	6 months	Not mentioned
Morley[19]	1999	UK	RCT	Infants (less than 24 months)	Targeted	ferrous sulphate in Formula milk	1.2 mg/L	9 months	Not mentioned
Moretti[20]	2006	India	RCT	Iron-depleted 6-13 year old children	Mass	ferric pyrophosphate in Rice-based meal 6d/week	200 mg/kg	7 month	Not mentioned
Muthayya[21]	2012	India	RCT	6 to 15 years old iron depleted children	Targeted	Wheat based lunch with NaFeEDTA fortification	6mg Fe/100g flour	28 weeks	Not mentioned
Nogueria[22]	2012	Brazil	RCT	Infants (less than 24 months)	Targeted	Rice with micronized ferric pyrophosphate	56.5mg/50g	18 weeks	Not mentioned
Nogueria[23]	2012	Brazil	RCT	Infants (less than 24 months)	Targeted	$FeSO_4$ in porridge	10mg per portion	14 weeks	Not mentioned
Rim[24]	2008	Korea	RCT	Infants 6-12 months age	Targeted	ferrous sulphate in Water in which rice porridge cooked	0.5g/kg	6 months	Not mentioned
Rohner[25]	2010	Cotˆ de ’Ivoire	RCT	6-14 year old school children	Targeted	Electrolytic iron in Biscuits 4 times/week	Not mentioned	6 month	Not mentioned
Sari[26]	2001	Indonesia	RCT	children age 4-6 years	Targeted	Elemental iron in candies (10/week over 3d)	1 g/kg	12 week	Not mentioned
Stevens[27]	1995	UK	RCT	Infants (less than 12 months)	Targeted	Ferrous Sulphate in Formula milk	12 mg/L	1 year	Not mentioned
Sketel[28]	1988	Chile	RCT	3 to 15 months old healthy infants	Targeted	full-fat acidified milk fortified with $FeS0_4$	15 mg of elemental Fe as ferrous sulfate/100g powder	1 year	Not mentioned
Singhal[29]	2000	UK	RCT	Infants (less than 12 months)	Targeted	Ferrous Sulphate in Formula milk	12 mg/L	1 year	69 µg/L
Shamah-Levy[30]	2008	Mexico	RCT	Toddlers 12-30 months	Targeted	Baby food	227.3 mg/kg	6 months	9090µg/kg
Semba[31]	2010	Indonesia	Survey	Children age 6-59 months	Mass	Milk and instant noodles	Not mentioned	Variable	Not mentioned

Schu¨mann[32]	2005	Guatemala	RCT	Children age 12-36 months	Targeted	Iron sulphate in refried black beans 5d/week	224 mg/kg	10 weeks	Not mentioned
Walter[33]	1993	Chile	RCT	Children of ages 2 to 15 years	Mass	Hb concentrate cookies	Not mentioned	3 years	Not mentioned
Walter[34]	1993	USA	RCT	4 months old infants	Targeted	Iron fortified infant cereal	Not mentioned	44 weeks	Not mentioned
Van Stuijvenberg [35]	2008	South Africa	RCT	Children age 6-11 years with Hb< 125 g/L	Targeted	Brown bread	Not mentioned	34 weeks	1196.6 μg/kg
Sun[36]	2007	China	RCT	anemic students 11-18 years old	Targeted	sodium iron ethylenediaminetraacetic acid (NaFeEDTA) in Wheat flour	Not mentioned	6 months	Not mentioned
Tuthill[37]	2002	UK	RCT	Infants (less than 12 months)	Targeted	formula milk	5 mg/L	3 months	Not mentioned
Virtanen[38]	2001	Sweden	RCT	Healthy 12 months old infants	Targeted	ferrous gluconate or ferrous lactate in cow's milk	7 mg/L, 14.9 mg/L	6 months	Not mentioned
Ziegler[39]	2009	USA	RCT	Infants (less than 12 months)	Targeted	Ferrous sulphate in cereal	61.9 mg/kg	2 years	Not mentioned
Zimmerman [40]	2010	Ivory	RCT	6 to 14 year old children	Targeted	Elemental Iron fortified biscuits	20mg Fe/day	24 weeks	Not mentioned
Zimmerman [41]	2006	India	RCT	5-9 year old children	Targeted	Iron fortified rice meal (ferric pyrophosphate)	15mg Fe/day	16 weeks	Not mentioned

Table S2 Study characteristics for zinc fortification in children

Zinc									
**Author**	**Year**	**Country**	**Study design**	**Target population**	**Fortification strategy**	**Fortification compound and Carrier**	**Amount of micronutrient used for fortification**	**Duration of intervention period evaluated**	**Target population daily micronutrient Intake**
Brown[42]	2007	Peru	RCT	Infants aged 6 to 8 months with risk of stunting	Targeted	Zinc Sulphate in Porridge	150 mg/kg dry weight	6 months	Not mentioned
Diaz Gomez[43]	2003	Spain	RCT	VLBW Preterm infants	Targeted	Zinc sulphate in Formula feed	10 mg/L (5 mg/L)	6 months	Not mentioned
Friel[44]	2003	Canada	RCT	VLBW Newborns	Targeted	Zinc Sulphate in Formula feed	11 mg/L (6.7 mg/L)	6 months	Not mentioned
Hambidge[45]	1979	USA	RCT	2-7 year old children	Targeted	Zinc oxide in cereals	3.75 mg/ounce	9 months	Not mentioned
Kilic[46]	1998	Turkey	RCT	7-11 year old school going children with asymptomatic zinc deficiency	Targeted	Zinc acetate in Bread	400 mg/ loaf	3 months	Not mentioned
Matsuda[47]	1984	Japan	Quasi	full term newborns	Targeted	Formula feed	3.2 mg/L (1.0 mg/L)	5 months	Not mentioned
Ohiokpehai[48]	2009	Kenya	Quasi	6-9 years old school going children	Targeted	Porridge	5 mg/100g	3 months	Not mentioned
Salmenpera[49]	1994	Finland	RCT	Full term Newborns	Targeted	Formula feed	1. 1.1 mg/L	12 months	Not mentioned
Schlesinger[50]	1992	Chile	RCT	Malnourished Infants	Targeted	Zinc chloride in Formula feed	15 mg/L (3.2 mg/L)	3.5 months	Not mentioned
Walravens[51]	1976	USA	RCT	Full term Newborns	Targeted	Zinc sulphate in Formula feed	5.8 mg/L (1.8 mg/L)	6 months	Not mentioned

Table S3 Study characteristics for Vitamin A fortification in children

Vitamin A									
**Author**	**Year**	**Country**	**Study design**	**Target population**	**Fortification strategy**	**Fortification compound and Carrier**	**Amount of micronutrient used for fortification**	**Duration of intervention period evaluated**	**Target population daily micronutrient Intake**
Arroyave[52]	1981	Guatemala	Before-After	Children aged 1 to 5 years	Targeted	Sugar	Not mentioned	2 years	Not mentioned
Chen[53]	2008	China	RCT	2-6 year old preschool children	Targeted	Vitamin A acetate in Seasoning powder	500 µg / 2.5g of powder	6 months	Not mentioned
Mejia[54] .	1982	Guatemala	Before-After	Preschool Children (ages less than 6 years)	Targeted	Sugar	Not mentioned	2 years	Not mentioned
Muhilal[55].	1988	Indonesia	Before-After	Children (0-5 years)	Targeted	MSG (Mono sodium glutamate)	810 µg /g MSG	1 year	Not mentioned
Ribaya-Mercado[56]	2004	Nicaragua	Before-After	Children aged 5 to 9 years	Targeted	Vitamin A acetate in Sugar	Not mentioned	1 year	Not mentioned
Solon[57]	2000	Philippines	RCT	Children aged 6 to 13 years	Targeted	Vitamin A palmitate n Flour (pandesal)	133 µg /pandesal (33% of RDA)	30 weeks	Not mentioned
Solon[58]	1979	Philippines	Quasi	Children 1-16 years	Targeted	MSG	15000 IU/packet	2 years	Not mentioned
Zhang[59]	2010	China	RCT	Children aged 3 to 6 years,	Targeted	Biscuits	Group 1: 500 IU / piece (30%,RDA) Group 2: 1666 IU/piece (100% RDA)	9 months	Not mentioned

Table S4 Study characteristics for Vitamin D and Calcium fortification in children

Vitamin D and calcium									
**Author**	**Year**	**Country**	**Study design**	**Target population**	**Fortification strategy**	**Fortification compound and Carrier**	**Amount of micronutrient used for fortification**	**Duration of intervention period evaluated**	**Target population daily micronutrient Intake**
Bonjour[60]	1997	Geneva	RCT	Pre-pubertal Girls (Range 6.6-9.4 years)	Targeted	cakes, biscuits, fruit juices, chocolate bars	Not mentioned	48 weeks	850 mg of calcium
Du[61]	2004	China	RCT	Children 10-12 years old	Targeted	Milk	560mg Calcium and 5-8 micrograms Vitamin D/500ml	2 year	245 mg Calcium and 3·33 mg cholecalciferol/day
Ganmaa [62]	2008	Mongolia	Before-After	Children (9-11 years)	Targeted	Milk	100 IU of Vitamin D/236ml of milk	1 month	300IU or 7.5 Vitamin D
Graham [63]	2008	New Zealand.	RCT	Children of ages 6 to 9 years	Targeted	Milk	Vitamin D3 1.5 µg Ca 480 mg	Not mentioned	Ca 60% RDA
Ho[64]	2005	China	Quasi	Adolescent girls (14-16 years)	Targeted	Soy milk	600 mg Ca / 375 ml	1years	600 mg of Calcium
Koo [65]	2006	USA	RCT	Preterm infants	Targeted	Formula Milk	105 mg Ca, 80 IU Vit D	1 year	Not mentioned
Rich-Edwards [66]	2011	Mongolia	RCT	Children (9-11 years)	Targeted	Milk	Not mentioned	2 months	Not mentioned
Veromaa [67]	2008	Finland	Before-After	Adolescent girls (12-18 years)	Targeted	Fluid milk and margarine	Not mentioned	4 years	73% of the participants consumed at least 800 mg Ca/d 16% consumed 7·5 µg vitamin D/d.
Zhu[68]	2005	China	RCT	Girls (10-12 years)	Targeted	Milk	1.7 mg/ml	24 months	245mgCa and, 3.33 µg vitamin D.
Zhu[69]	2008	China	RCT	Girls (10-12 years)	Targeted	Milk	Calcium 560mg/330ml Vit D 5-8 µg	24 months	245 mg calcium, 3.33 µg vit D

Table S5 Study characteristics for folate fortification in children

Folate									
Author	Year	Country	Study design	Target population	Fortification strategy	Fortification compound and Carrier	Amount of micronutrient used for fortification	Duration of intervention period evaluated	Target population daily micronutrient Intake
Grupp[70]	2011	Canada	Before-After	children aged 9 and below	Mass	Flour	Not mentioned	9 years	Not mentioned
Wiltshire[71]	2004	Australia	Before-After	Children (10 to 14 years)	Mass	cereal and bread products	Not mentioned	1 year	Not mentioned

Table S6 Study characteristics for iodine fortification in children

Iodine								
Author	Year	Country	Study design	Target population	Fortification strategy	Fortification compound and Carrier	Amount of micronutrient used for fortification	Target population daily micronutrient Intake
Delange [72]	1999	Africa	Before-After	Children of ages 6 to 14 years	Mass	Salt	Not Mentioned	Not mentioned
Guttikonda [73]	2002	Australia	Before-After	Children of ages 4 to 17 years	Mass	Salt	Not Mentioned	Not mentioned
Jooste [74]	2000	South africa	Before-After	Children of ages 8 to 16 years	Mass	Salt	40–60 mg/g	Not mentioned
Pongpaew [75]	1998	Thailand	Before-After	Children of ages 5 to 17 years	Mass	Salt	50 g/100 kg 200μg 80μg	Not mentioned
Yamamah [76]	2003	Egypt	Before-After	Girls of ages 8 to 16 years	Mass	Salt	50-80 ppm	Not mentioned
Zimmermann[77]	2003	Côte d ’ivoire	Before-After	Children of ages 5 to 17 years	Mass	Salt	30–50 ppm	Not mentioned
Zimmermann [78]	2006	Morocco Albania South Africa	Before-After	Children of ages 5 to 17 years	Mass	Salt	25ppm	Not mentioned

Table S7 Study characteristics for dual fortification in children

Dual Fortification									
**Author**	**Year**	**Country**	**Study design**	**Target population**	**Fortification strategy**	**Fortification Vehicle**	**Amount of micronutrient used for fortification**	**Duration of intervention period evaluated**	**Micronutrients used**
Andersson[79]	2008	India	RCT	Children(5-15 years)	Targeted	Salt	both at 2 mg/g salt	1 year	Iodine, iron
Nugroho[80]	2010	Indonesia	RCT	Children (7-9 year old)	Targeted	Milk	Iron 12.15 mg Zn 4.4 mg	3 months	Fe,Zn
Sivakumar[81]	2002	India	RCT	Children (14-17 years)	Targeted	Salt	Not mentioned	2 years	Iodine, iron
Vinodkumar[82]	2009	India	RCT	Children(7-11 years)	Targeted	Salt	28 mg of elemental iron 1 mg of riboflavin	1 year	Iron Riboflavin
Wegmuller[83]	2006	Coˆ te d'Ivoire	QUASI	Children (5-15 years)	Targeted	Salt	3 mg Fe/g 53.8 6 18.2 mg I /g salt	6 months	Iodine, iron
Zimmermann[84]	2003	Morocco	RCT	Children (6-15 years)	Targeted	Salt	25 μg/g salt. 1 mg Fe/g salt	40 weeks	Iodine, iron
Zimmermann[85]	2004	Morocco	RCT	Children (6-15 years)	Targeted	Salt	25 g I (as potassium iodate)/g salt and 2 mg Fe/g salt	10 months	Iodine, iron
Zimmermann[86]	2002	Morocco	RCT	Children (6-15 years)	Targeted	Salt	25 mg iodine/g salt (1 mg Fe/g salt	10 months	Iodine, iron
Zlotkin[87]	2001	Ghana	RCT	Infants (less than 24 months)	Targeted	Meals	Not mentioned	2months	Fe, Ascorbic Acid
Zlotkin[88]	2003	Ghana	RCT	Infants (less than 24 months)	Targeted	Weaning foods	(80 mg of elemental iron 10 mg of zinc)	2 Months	Fe,Zn

Table S8 Study characteristics for multiple-micronutrient fortification in children

Multiple Micronutrients									
**Author**	**Year**	**Country**	**Study design**	**Target population**	**Fortification strategy**	**Fortification compound and Carrier**	**Number of micronutrients used**	**Duration of intervention period evaluated**	**Target population daily micronutrient Intake**
Chen[89]	2011	China	RCT	Children (2-6 years)	Targeted	Food	8	6 months	Not mentioned
Chen[90]	2008	China	RCT	Children (2-6 years)	Targeted	Food	8	6 months	Not mentioned
Faber[91]	2005	South Africa.	RCT	Children (6-12 months)	Targeted	maize meal	10	6 months	Not mentioned
Gershoff[92]	1977	Thailand	Quasi	Children (1.5-9 years)	Targeted	Rice	6	4 years	Not mentioned
Gibson[93]	2011	Zambia	RCT	Children (6 months)	Targeted	Porridge	19	12 months	Not mentioned
Goyle[94]	2010	India	Before-After	adolescent girls (10-16 years)	Targeted	Biscuits	5	4 months	Not mentioned
Kumar[95]	2007	India	Quasi	Children(7-11 years)	Targeted	Salt	10	1 year	Not mentioned
Kuusipalo[96]	2006	Malawi	RCT	6-17 months underweight infants	Targeted	fortified spread	20	12 weeks	Not mentioned
Lartey[97]	1999	Ghana	RCT	Infants (6-12 month)	Targeted	maize corn	16	1 year	Not mentioned
Layrisse[98]	1996	Venezuela	Before-After	Children (7-15 years)	Targeted	Maize	3	1 year	Not mentioned
Lien[99]	2009	Vietnam	RCT	Children (7-8 years)	Targeted	Milk	19	6 months	Not mentioned
Lin[100]	2008	Malawi	RCT	Children (6 months)	Targeted	peanut-/soy-based fortified spread vs corn porridge	14	1 year	Not mentioned
Liu[101]	1993	China	RCT	Children (6-13 months)	Targeted	Rusk	10	3 months	Not mentioned
Lopriore[102]	2004	Algeria	RCT	Children (3-6 years)	Targeted	Spread	18	6 months	Not mentioned
Loui[103]	2004	Germany	RCT	Preterm infants (<33 weeks)	Targeted	human milk	7	6 weeks	Not mentioned
Lucas[104]	1996	United kingdom	RCT	Preterm infants (23-36 weeks)	Targeted	human milk	20	18 months	Not mentioned
Lutter[105]	2007	Santo Domingo	quasi	Children (9-14 months)	Targeted	Milk	18	1 year	Not mentioned
Manger[106]	2008	Thailand	RCT	Children (5.5-13.4 years)	Targeted	Food	4	31 week	Not mentioned

Manno[107]	2011	Zambia	RCT	Children (6 months)	Targeted	Porridge	18	1 year	Not mentioned
Morgan[108]	2010	New Zealand	RCT	Infants (12-20 months)	Targeted	red meat and milk	5	20 weeks	Not mentioned
Nesamvuni[109]	2005	Africa	RCT	Children (1-3 years)	Targeted	maize meal	4	12months	Not mentioned
Nga[110]	2009	Vietnam	quasi	Children (6-8year)	Targeted	Biscuits	11	4 months	Not mentioned
Osei[111]	2010	India	RCT	Children (6-10 year)	Targeted	Meal	5	10 months	Not mentioned
Oue´draogo[112]	2010	Burkina Faso	RCT	Children (6-23 months)	Targeted	ingredient-based gruel	5	6 months	Not mentioned
Phu[113]	2010	Vietnam	RCT	Infants (5 month old)	Targeted	flour (FF) or a food complement (FC)	6	6 months	Not mentioned
Rivera[114]	2010	Mexico	RCT	Children(12-30 months)	Targeted	Milk	9	12 months	Not mentioned
Sazawal[115]	2010	India	RCT	Children (1-3 years)	Targeted	Milk	24	1 year	Not mentioned
Seal [116]	2007	Zambia	Before-After	Adolescents (10-18 years)	Targeted	maize meal	9	12 months	Not mentioned
Stekel[117]	1988	Chile	RCT	Children (3-15 months)	Targeted	Milk	4	3-15 months	Not mentioned
Stuijvenberg[118]	1999	South Africa	RCT	Children (6-11 years)	Targeted	Biscuits	3	43 weeks over a 12 months	Not mentioned
Torrejo´n[119]	2004	Chile	RCT	Infants (< 6 months)	Targeted	Milk	3	12 months	Not mentioned
Varma[120]	2007	India	RCT	Children (36-66 months)	Targeted	Khichdi	3	24 weeks	Not mentioned
Villalpando,[121]	2006	Mexico	RCT	Children (10-30 months)	Targeted	Milk	5	6 months	Not mentioned
Winichagoon[122]	2006	Thailand	RCT	Children (5.5-14 years)	Targeted	Lunch	4	31 weeks	Not mentioned

Table S9 Study characteristics for iron fortification in women

Author	Year	Country	Study design	Target population	Fortification strategy	Fortification compound and Carrier	Amount of micronutrient used for fortification	Duration of intervention period evaluated	Target population daily micronutrient Intake
**Iron**									
Andersson[123]	2010	Switzerland	RCT	18-40 year old females with low serum ferritin	Targeted	Microionised pyrophosphate or NaFeEDTA in margarine	Not mentioned	32 weeks	Not mentioned
Biebinger[124]	2009	Kuwait	RCT	College women ages 18-35	Targeted	Ferrous sulphate in Wheat-flour biscuits	800 mg/kg	22 weeks	Not mentioned
Blanco-Rojo[125]	2012	Spain	RCT	18-35 year old females with Fe deficiency	Targeted	Iron phoshate in Fruit juice	Not mentioned	16 weeks	Not mentioned
Blanco-Rojo[126]	2011	Spain	RCT	Women aged 18–35 years	Targeted	Iron phoshate in Fruit juice	36mg/L	16 weeks	Not mentioned
Chen[8]	2005	China	RCT	Male and female above the age of 3 years	Targeted	NaFeEDTA in Soy sauce	296mg/l	18 months	NR- baseline and final plasma retinol reported
Hoa[127]	2005	Vietnam	Cluster (communes)	Pregnant women ages 20-32 years	Targeted	Ferrous Fumarate in Milk	37.5 mg/L	16 weeks	Not mentioned
Hotz[128]	2008	Mexico	RCT	Women 18-49 years of age	Targeted	micronized ferric pyrophosphate in Rice 5d/week	266.7 mg/Kg	6 months	Not mentioned
Karl[129]	2010	USA	RCT	Female army recruits	Targeted	ferrous sulfate in Food bars	797 mg/kg	9 week	Not mentioned
Sadighi[130]	2008	Iran	Survey	Women age 15-49 years	Targeted	Ferros sulphate in Wheat flour	Not mentioned	3 years	Not mentioned
Sadighi[131]	2009	Iran	Before-After	Women age 15-49 years	Targeted	Ferrous sulphate in Wheat flour	Not mentioned	8 years	Not mentioned
Thuy[132]	2003	Vietnam	RCT	17-49 year old non-pregnant women > 80 but < 120 g/L	Targeted	NaFeEDTA in Fish sauce 6d/week	1 g/L	6 months	Not mentioned
Thuy[133]	2005	Vietnam	RCT	Non-pregnant woman between 16 and 49 y of age	Targeted	NaFeEDTA in fish sauce	500 mg/L	18 months	0.1 mg/d
Zimermman[134]	2005	Thailand	RCT	18-50 year old women	Targeted	Fortified snacks with $FeSO_4$, Electrolytic iron and hydrogen based iron	Not mentioned	35 weeks	NR

Table S10 Study characteristics for zinc fortification in women

Zinc									
Author	Year	Country	Study design	Target population	Fortification strategy	Fortification compound and Carrier	Amount of micronutrient used for fortification	Duration of intervention period evaluated	Target population daily micronutrient Intake
Badii[135]	2012	Iran	RCT	Zinc-deficient women aged 19 to 49 years	Targeted	Zinc sulfate in bread	50 and 100 mg/Kg	1 month	Not mentioned

Table S11 Study characteristics for folate fortification in women

Folate									
Author	Year	Country	Study design	Target population	Fortification strategy	Fortification compound and Carrier	Amount of micronutrient used for fortification	Duration of intervention period evaluated	Target population daily micronutrient Intake
Alasfoor[136]	2008	Oman	Before-After	Pregnant women	Mass	Flour	5mg/kg	10 years	Not mentioned
Amarin[137]	2010	Jordon	Before-After	WRA*	Mass	grain products	Not mentioned	3 years	Not mentioned
Bar-oz[138]	2008	Canada	Before-After	WRA (14-45)	Mass	Flour	0.15mg/100gm	8 years	Not mentioned
Bol[139]	2006	US	Before-After	WRA	Mass	Flour	40 µg /100 g	3 years	Not mentioned
Boulet[140]	2008	US	Before-After	WRA	Mass	cereal grain products	40 µg /100 g	5 years	Not mentioned
Canfield[141]	2005	US	Before-After	WRA	Mass	Flour	40 µg /100 g	2 years	Not mentioned
Chen[142]	2004	Costa Rica	Before-After	WRA (15-44 years)	Mass	wheat and corn flour	1.8mg/kg	3 years	108 µg / day
Chen[143]	2008	US	Before-After	WRA	Mass	cereal grain products	40 µg /100 g	7 years	Not mentioned
Hertrampf[144]	2003	Chile	Before-After	WRA	Mass	Wheat Flour	2.2 mg/kg	1 year	400 µg / day
Honein[145]	2001	USA	Before-After	WRA	Mass	Flour	40 µg /100 g	1 year	Not mentioned
Ionescu-ittu[146]	2009	Canada	Before-After	WRA	Mass	Flour & Pasta products	Not mentioned	7 years	Not mentioned
Kucik[147]	2004	USA	Before-After	WRA	Mass	Cereal products	40 µg /100 g	Not mentioned	Not mentioned

Lawrence[148]	2004	USA	Before-After	WRA	Mass	cereal grain products	40 µg /100 g	2 years	Not mentioned
Liu[149]	2004	Canada	Before-After	WRA	Mass	Grain products	Not mentioned	1 years	Not mentioned
Lopez-Comello[150]	2005	Chile	Before-After	WRA	Mass	Wheat Flour	Not mentioned	5 years	Not mentioned
Modjadji[151]	2007	South Africa	Before-After	WRA (18-44)	Mass	Staple Food	Not mentioned	1 year	250 µg / day
Murphy[152]	2000	UK	Before-After	WRA	Mass	cereal and bread	Not mentioned	Not mentioned	Not mentioned
Orioli[153]	2011	Brazil	Before-After	WRA	Mass	wheat and maize flour	150 µg /100g	2 years	Not mentioned
Pacheco[154]	2009	Brazil	Before-After	WRA	Mass	Wheat	150 µg /100g	2 years	Not mentioned
Persad[155]	2002	Canada	Before-After	WRA	Mass	Grains	0.15mg/100g	3 years	Not mentioned
Ray[156]	2004	Canada	Before-After	WRA	Mass	cereal grains	0.15mg/100g	3 years	Not mentioned
Ray[157]	2003	Canada	Before-After	WRA	Mass	cereal grains	0.15mg/100g	3 years	Not mentioned
Robbins[158]	2006	USA	Before-After	WRA	Mass	cereal grains	40 µg /100 g	4 years	Not mentioned
Safdar[159]	2007	Saudi Arabia	Before-After	WRA	Mass	Flour	16.53 g of folic in 1000g	4 years	Not mentioned
Sayed[160]	2008	South Africa	Before-After	WRA	Mass	Staple foods	Not mentioned	1 year	Not mentioned
Shaw[161]	2003	USA	Before-After	WRA	Mass	grain products	40 µg /100 g	1 year	Not mentioned
Signore[162]	2005	USA	Before-After	WRA	Mass	grain product s	40 µg /100 g	2 years	Not mentioned
Simmons[163]	2004	USA	Before-After	WRA	Mass	grain products	40 µg /100 g	2 years	Not mentioned
Waller[164]	2003	USA	Before-After	WRA	Mass	Grains	40 µg /100 g	1 year	Not mentioned
Williams[165]	2002	USA	Before-After	WRA	Mass	Grains	40 µg /100 g	1 year	Not mentioned

*WRA: Women of reproductive age

Table S12 Study characteristics for Vitamin D and calcium fortification in women

Vitamin D and calcium									
**Author**	**Year**	**Country**	**Study design**	**Target population**	**Fortification strategy**	**Fortification compound and Carrier**	**Amount of micronutrient used for fortification**	**Duration of intervention period evaluated**	**Target population daily micronutrient Intake**
Adolphi [166]	2009	Germany	RCT	Post-Menopausal (48-67 years)	Targeted	fermented milk	Control 210 mg Ca and Intervention 510 mg Ca	2 weeks	Not mentioned
Bonjour [167]	2009	France	Before-After	Elderly Women (Mean Age 84.8 years)	Targeted	Soft plain cheese	Calcium: 151 mg/100g of cheese	30 days	2·5 µg vitamin D, 302 mg Ca (7-45% RDA)
Chee [168]	2003	Malaysia	RCT	Post Menopausal (55-65 years)	Targeted	Milk	24 mg/g of skimmed milk	24 months	1200 mg calcium/ day
Ferrar [169]	2010	UK	RCT	WRA* (20-39 years)	Targeted	Ice cream	Group 1: Ca 244 mg<br>Group 2: 459 mg<br>Group 3: 676 mg	28 days	Group 1: Ca 244mg<br>Group 2: 459mg<br>Group 3: 676 mg
Green [170]	2010	New Zealand.	RCT	Women (18-47 years)	Targeted	Milk	vitamin D (5+)	12 weeks	5ug of Vitamin D
Kruger[171]	2006	New Zealand	RCT	Pre-Menopausal Women (20-35 years)	Targeted	Skimmed milk	1000 mg of calcium, 5 g of vitamin D3	16 weeks	Not mentioned
Kruger[172]	2010	Indonesia Philippines	Quasi	Postmenopausal women (>55 years of age)	Targeted	Milk	1200 mg calcium and 9.6 µg vit D per 60 g serving	16 weeks	Not mentioned
Lau[173]	2001	China	RCT	Postmenopausal women (55-59 years)	Targeted	Milk	40mg/g	2 years	800 mg
Manios [174]	2009	Greece	Quasi	Post-menopausal women (55-65 years)	Targeted	Dairy products	Not mentioned	3 months	1200 mg of calcium and 7.5 µg of vitamin D3
Moschonis [175]	2006	Greece	RCT	post-menopausal women (55-65 years)	Targeted	Dairy products	Not mentioned	12 months	1200 mg calcium/d and 7·5 mg vitamin D3/d
Natri [176]	2006	Denmark	RCT	Women (25- 45 years)	Targeted	Bread	10 µg/100g	3 weeks	10 µg Vit D
Recker [177]	1985	USA	RCT	Postmenopausal women (45-70years)	Targeted	Milk	Not mentioned	24 months	Not mentioned
Storm [178]	1998	USA	RCT	Elderly women (>65years)	Targeted	Milk	Not mentioned	2 years	(CaCO3-1000 mg/day
Tenta [179]	2010	Greece	RCT	Postmenopausal women (55-65 years)	Targeted	Dairy products	Not mentioned	30 months	1,200 mg of calcium and 7.5 µg of vitamin D

*WRA: Women of reproductive age

Table S13 Study characteristics for iodine fortification in women

Iodine								
**Author**	**Year**	**Country**	**Study design**	**Target population**	**Fortification strategy**	**Fortification compound and Carrier**	**Amount of micronutrient used for fortification**	**Target population daily micronutrient Intake**
Burgees [180]	2007	Australia	Before-After	Women	Mass	Salt in bread	Not Mentioned	Not mentioned
Camargo [181]	2008	Brazil	RCT	Women	Mass	Salt	Not Mentioned	Not mentioned
Carle[182]	2006	Denmark	Before-After	Women	Mass	Salt and salt in bread	13ppm	Not mentioned
Cerqueira [183]	2009	Denmark	Before-After	Women	Mass	Salt	13ppm	Not mentioned
Cerqueira [184]	2011	Denmark	Before-After	Women	Mass	Salt	Not Mentioned	Not mentioned
Laurberg [185]	2006	Denmark	Before-After	Women	Mass	Salt	8ppm	Not mentioned
Moleti [186]	2008	Italy	Before-After	Pregnant women	Mass	Salt	Not Mentioned	Not mentioned
Moleti [187]	2011	Italy	Before-After	Pregnant women	Mass	Salt	150µg	Not mentioned
Pedersen [188]	2007	Denmark	Before-After	Women	Mass	Salt	8ppm than 13ppm	Not mentioned
Pedersen [189]	2011	Denmark	Before-After	Women	Mass	Salt	Not Mentioned	Not mentioned
Rasmussen [190]	2008	Denmark	Before-After	Women	Mass	Salt and bread	Not Mentioned	Not mentioned
Seal [191]	2007	Australia	Before-After	General	Mass	Bread	Not Mentioned	Not mentioned
Sebotsa [192]	2005	Lesotho	Before-After	General	Mass	Salt	40 to 60 ppm	Not mentioned
Sultanalieva [193]	2009	Kyrgyzstan	RCT	General	Mass	Salt	25–55 mg/kg	Not mentioned
Vejbjerg [194]	2007	Denmark	Before-After	Women	Mass	Salt	13ppm	Not mentioned
Vejbjerg [195]	2009	Denmark	Before-After	Women	Mass	Salt	13ppm	Not mentioned

Table S14 Study characteristics for dual fortification in women

Dual Fortification									
Author	Year	Country	Study design	Target population	Fortification strategy	Fortification compound and Carrier	Amount of micronutrient used for fortification	Duration of intervention period evaluated	Target population daily micronutrient Intake
Viteri[196]	1995	Guatemala	RCT	General Population	Mass	sugar	Iron<br>Vit A	32 month	Not mentioned

Table S15 Study characteristics for multiple-micronutrient fortification in women

Multiple Micronutrients									
Author	Year	Country	Study design	Target population	Fortification strategy	Fortification compound and Carrier	Number of micronutrients	Duration of intervention period evaluated	Target population daily micronutrient Intake
Adu-Afarwuah[197]	2007	Ghana	RCT	General	Targeted	Homemade food	19	1.5 years	Not mentioned
Huo[198]	2011	China	Quasi	Female (20-60 years)	Targeted	Flour	7	3 years	Not mentioned
Mardones[199]	2007	Chile	RCT	Pregnant women	Targeted	Food	20	9 months	Not mentioned
Tapola[200]	2004	Finland	RCT	Men & Women (26-65 years)	Targeted	Mineral Water	5	8 weeks	Not mentioned
Tucker[201]	2004	US	RCT	Volunteers (50-85 years)	Targeted	Breakfast	3	14 weeks	Not mentioned

REFERENCES

1. Abizari AR, Moretti D, Zimmermann MB, Armar-Klemesu M, Brouwer ID: **Whole Cowpea Meal Fortified with NaFeEDTA Reduces Iron Deficiency among Ghanaian School Children in a Malaria Endemic Area**. *The Journal of nutrition* 2012, **142**(10):1836-1842.
2. Andang'o PE, Osendarp SJ, Ayah R, West CE, Mwaniki DL, De Wolf CA, Kraaijenhagen R, Kok FJ, Verhoef H: **Efficacy of iron-fortified whole maize flour on iron status of schoolchildren in Kenya: a randomised controlled trial**. *Lancet* 2007, **369**(9575):1799-1806.
3. Arcanjo FP, Amancio OM, Braga JA, de Paula Teixeira Pinto V: **Randomized controlled trial of iron-fortified drinking water in preschool children**. *Journal of the American College of Nutrition* 2010, **29**(2):122-129.
4. Williams J, Wolff A, Daly A, MacDonald A, Aukett A, Booth IW, Logan S: **Iron supplemented formula milk related to reduction in psychomotor decline in infants from inner city areas: randomised studyCommentary: Iron deficiency and developmental deficit–the jury is still out**. *Bmj* 1999, **318**(7185):693.
5. Barbosa TN, Taddei JA, Palma D, Ancona-Lopez F, Braga JA: **Double-blind randomized controlled trial of rolls fortified with microencapsulated iron**. *Rev Assoc Med Bras* 2012, **58**(1):118-124.
6. Beinner MA, Lamounier JA, Tomaz C: **Effect of iron-fortified drinking water of daycare facilities on the hemoglobin status of young children**. *Journal of the American College of Nutrition* 2005, **24**(2):107.
7. Beinner MA, Velasquez-Meléndez G, Pessoa MC, Greiner T: **Iron-Fortified Rice Is As Efficacious As Supplemental Iron Drops in Infants and Young Children**. *The Journal of nutrition* 2010, **140**(1):49.
8. Chen J, Zhao X, Zhang X, Yin S, Piao J, Huo J, Yu B, Qu N, Lu Q, Wang S: **Studies on the effectiveness of NaFeEDTA-fortified soy sauce in controlling iron deficiency: a population-based intervention trial**. *Food &# 38; Nutrition Bulletin* 2005, **26**(2):177-186.
9. de Almeida CA, Dutra-De-Oliveira JE, Crott GC, Cantolini A, Ricco RG, Del Ciampo LA, Baptista ME: **Effect of fortification of drinking water with iron plus ascorbic acid or with ascorbic acid alone on hemoglobin values and anthropometric indicators in preschool children in day-care centers in Southeast Brazil**. *Food and nutrition bulletin* 2005, **26**(3):259-265.
10. Gill DG, Vincent S, Segal DS: **Followâ€on formula in the prevention of iron deficiency: a multicentre study**. *Acta Paediatrica* 1997, **86**(7):683-689.
11. Giorgini E, Fisberg M, De Paula RA, Ferreira AM, Valle J, Braga JA: **The use of sweet rolls fortified with iron bis-glycinate chelate in the prevention of iron deficiency anemia in preschool children**. *Archivos Latinoamericanos de Nutricion* 2001, **51**(1):48-53.
12. Haschke F, Pietschnig B, Vanura H, Heil M, Steffan I, Hobiger G, Schuster E, Camaya Z: **Iron intake and iron nutritional status of infants fed iron-fortified beikost with meat**. *The American journal of clinical nutrition* 1988, **47**(1):108.
13. Huo J, Sun J, Miao H, Yu B, Yang T, Liu Z, Lu C, Chen J, Zhang D, Ma Y: **Therapeutic effects of NaFeEDTAâ€fortified soy sauce in anaemic children in China**. *Asia Pacific journal of clinical nutrition* 2002, **11**(2):123-127.
14. Iost C, Jeppsen RB, Ashmead HDW: **Repleting hemoglobin in iron deficiency anemia in young children through liquid milk fortification with bioavailable iron amino acid chelate**. *Journal of the American College of Nutrition* 1998, **17**(2):187-194.
15. Le Huong T, Inge B, Jan B, Khan N, Frans K: **Efficacy of iron fortification compared to iron supplementation among Vietnamese schoolchildren**. *Nutrition Journal* 2006, **5**.

16. Longfils P, Monchy D, Weinheimer H, Chavasit V, Nakanishi Y, Schumann K: **A comparative intervention trial on fish sauce fortified with NaFe-EDTA and FeSO4+ citrate in iron deficiency anemic school children in Kampot, Cambodia**. *Asia Pacific journal of clinical nutrition* 2008, **17**(2):250-257.
17. Marsh A, Long H, Stierwalt E: **Comparative hematologic response to iron fortification of a milk formula for infants**. *Pediatrics* 1959, **24**:404-412.
18. Miglioranza LH, BreganÃ³ JW, Dichi I, Matsuo T, Dichi JB, Barbosa DS: **Effectiveness of fortification of corn flour-derived products with hydrogen-reduced elemental iron on iron-deficiency anaemia in children and adolescents in southern Brazil**. *Public Health Nutr* 2009, **12**(02):244.
19. Morley R, Abbott R, Fairweather-Tait S, MacFadyen U, Stephenson T, Lucas A: **Iron fortified follow on formula from 9 to 18 months improves iron status but not development or growth: a randomised trial**. *Archives of disease in childhood* 1999, **81**(3):247-252.
20. Moretti D, Zimmermann MB, Muthayya S, Thankachan P, Lee TC, Kurpad AV, Hurrell RF: **Extruded rice fortified with micronized ground ferric pyrophosphate reduces iron deficiency in Indian schoolchildren: a double-blind randomized controlled trial**. *American Journal of Clinical Nutrition* 2006, **84**(4):822.
21. Muthayya S, Thankachan P, Hirve S, Amalrajan V, Thomas T, Lubree H, Agarwal D, Srinivasan K, Hurrell RF, Yajnik CS *et al*: **Iron Fortification of Whole Wheat Flour Reduces Iron Deficiency and Iron Deficiency Anemia and Increases Body Iron Stores in Indian School-Aged Children**. *The Journal of nutrition* 2012.
22. Nogueira Arcanjo FP, Roberto Santos P, Arcanjo CP, Amancio OM, Braga JA: **Use of Iron-Fortified Rice Reduces Anemia in Infants**. *Journal of tropical pediatrics* 2012.
23. Nogueira Arcanjo FP, Costa Arcanjo C, Nogueira Arcanjo FC, de Albuquerque Campos L, Silverio Amancio OM, Pellegrini Braga JA: **Milk-based Cornstarch Porridge Fortified with Iron is Effective in Reducing Anemia: A Randomized, Double-blind, Placebo-controlled Trial**. *Journal of tropical pediatrics* 2012, **58**(5):370-374.
24. Rim HY, Kim SH, Sim BC, Gang HY, Kim HY, Kim YR, Kim RC, Yang MH, Kim SP: **Effect of iron fortification of nursery complementary food on iron status of infants in the DPRKorea**. *Asia Pacific journal of clinical nutrition* 2008, **17**(2):264-269.
25. Rohner F, Zimmermann MB, Amon RJ, Vounatsou P, Tschannen AB, N'Goran EK, Nindjin C, Cacou MC, TÃ©-BonlÃ© MD, Utzinger J: **In a randomized controlled trial of iron fortification, anthelmintic treatment, and intermittent preventive treatment of malaria for anemia control in Ivorian children, only anthelmintic treatment shows modest benefit**. *Journal of Nutrition* 2010, **140**(3):635.
26. Sari M, Bloem MW, de Pee S, Schultink WJ, Sastroamidjojo S: **Effect of iron-fortified candies on the iron status of children aged 4â€“6 y in East Jakarta, Indonesia**. *American Journal of Clinical Nutrition* 2001, **73**(6):1034.
27. Stevens D, Nelson A: **The effect of iron in formula milk after 6 months of age**. *Archives of disease in childhood* 1995, **73**(3):216.
28. Stekel A, Olivares M, Cayazzo M, Chadud P, Llaguno S, Pizarro F: **Prevention of iron deficiency by milk fortification. II. A field trial with a full-fat acidified milk**. *Am J Clin Nutr* 1988, **47**(2):265-269.
29. Singhal A, Morley R, Abbott R, Fairweather-Tait S, Stephenson T, Lucas A: **Clinical safety of iron-fortified formulas**. *Pediatrics* 2000, **105**(3):e38.

30. Shamah-Levy T, Villalpando S, Rivera-Dommarco JA, Mundo-Rosas V, Cuevas-Nasu L, JimÃ©nez-Aguilar A: **Ferrous gluconate and ferrous sulfate added to a complementary food distributed by the Mexican nutrition program Oportunidades have a comparable efficacy to reduce iron deficiency in toddlers**. *Journal of pediatric gastroenterology and nutrition* 2008, **47**(5):660.
31. Semba RD, Moench-Pfanner R, Sun K, de Pee S, Akhter N, Rah JH, Campbell AA, Badham J, Bloem MW, Kraemer K: **Iron-fortified milk and noodle consumption is associated with lower risk of anemia among children aged 6â€“59 mo in Indonesia**. *American Journal of Clinical Nutrition* 2010, **92**(1):170.
32. Schümann K, Romero-Abal ME, MÃ¤urer A, Luck T, Beard J, Murray-Kolb L, Bulux J, Mena I, Solomons NW: **Haematological response to haem iron or ferrous sulphate mixed with refried black beans in moderately anaemic Guatemalan pre-school children**. *Public health nutrition* 2005, **8**(06):572-581.
33. Walter T, Hertrampf E, Pizarro F, Olivares M, Llaguno S, Letelier A, Vega V, Stekel A: **Effect of bovine-hemoglobin-fortified cookies on iron status of schoolchildren: a nationwide program in Chile**. *Am J Clin Nutr* 1993, **57**(2):190-194.
34. Walter T, Dallman PR, Pizarro F, Velozo L, Pena G, Bartholmey SJ, Hertrampf E, Olivares M, Letelier A, Arredondo M: **Effectiveness of iron-fortified infant cereal in prevention of iron deficiency anemia**. *Pediatrics* 1993, **91**(5):976-982.
35. van Stuijvenberg ME, Smuts CM, Lombard CJ, Dhansay MA: **Fortifying brown bread with sodium iron EDTA, ferrous fumarate, or electrolytic iron does not affect iron status in South African schoolchildren**. *Journal of Nutrition* 2008, **138**(4):782.
36. Sun X, Guo Y, Wang S, Sun J: **Social marketing improved the consumption of iron-fortified soy sauce among women in China**. *Journal of Nutrition Education and Behavior* 2007, **39**(6):302-310.
37. Tuthill DP, Cosgrove M, Dunstan F, Stuart ML, Wells JCK, Davies DP: **Randomized doubleâ€�blind controlled trial on the effects on iron status in the first year between a no added iron and standard infant formula received for three months***. *Acta Paediatrica* 2002, **91**(2):119-124.
38. Virtanen MA, Svahn CJE, Viinikka LU, Raiha NCR, Siimes MA, Axelsson IEM: **Ironâ€�fortified and unfortified cow's milk: Effects on iron intakes and iron status in young children**. *Acta Paediatrica* 2001, **90**(7):724-731.
39. Ziegler EE, Nelson SE, Jeter JM: **Iron status of breastfed infants is improved equally by medicinal iron and iron-fortified cereal**. *American Journal of Clinical Nutrition* 2009, **90**(1):76.
40. Zimmermann MB, Chassard C, Rohner F, N'Goran E K, Nindjin C, Dostal A, Utzinger J, Ghattas H, Lacroix C, Hurrell RF: **The effects of iron fortification on the gut microbiota in African children: a randomized controlled trial in Cote d'Ivoire**. *Am J Clin Nutr* 2010, **92**(6):1406-1415.
41. Zimmermann MB, Muthayya S, Moretti D, Kurpad A, Hurrell RF: **Iron fortification reduces blood lead levels in children in Bangalore, India**. *Pediatrics* 2006, **117**(6):2014-2021.
42. Brown KH, Lopez de Romana D, Arsenault JE, Peerson JM, Penny ME: **Comparison of the effects of zinc delivered in a fortified food or a liquid supplement on the growth, morbidity, and plasma zinc concentrations of young Peruvian children**. *Am J Clin Nutr* 2007, **85**(2):538-547.
43. Diaz-Gomez NM, Domenech E, Barroso F, Castells S, Cortabarria C, Jimenez A: **The effect of zinc supplementation on linear growth, body composition, and growth factors in preterm infants**. *Pediatrics* 2003, **111**(5):1002.

44. Friel JK, Andrews WL, Matthew JD, Long DR, Cornel AM, Cox M, McKim E, Zerbe GO: **Zinc supplementation in very-low-birth-weight infants**. *J Pediatr Gastroenterol Nutr* 1993, **17**(1):97-104.
45. Hambidge KM, Chavez MN, Brown RM, Walravens PA: **Zinc nutritional status of young middle-income children and effects of consuming zinc-fortified breakfast cereals**. *Am J Clin Nutr* 1979, **32**(12):2532.
46. Kiliç I, Özalp I, Coskun T, Tokatli A, Emre S, Saldamli I, Köksel H, Ozboy O: **The effect of zinc-supplemented bread consumption on school children with asymptomatic zinc deficiency**. *Journal of pediatric gastroenterology and nutrition* 1998, **26**(2):167.
47. Matsuda I, Higashi A, Ikeda T, Uehara I, Kuroki Y: **Effects of zinc and copper content of formulas on growth and on the concentration of zinc and copper in serum and hair**. *Journal of pediatric gastroenterology and nutrition* 1984, **3**(3):421.
48. Ohiokpehai OMD, Dorcus; Kamau, Joyce: **Serum zinc levels of school children on a corn-soy blend feeding trial in primary schools in Suba district, Kenya**. *J Appl Biosci* 2009, **17**:904 - 912.
49. Salmenpera L, Perheentupa J, Pakarinen P, Siimes MA: **Zinc supplementation of infant formula**. *Am J Clin Nutr* 1994, **59**(5):985.
50. Schlesinger L, Arevalo M, Arredondo S, Diaz M, Lonnerdal B, Stekel A: **Effect of a zinc-fortified formula on immunocompetence and growth of malnourished infants**. *Am J Clin Nutr* 1992, **56**(3):491.
51. Walravens PA, Hambidge KM: **Growth of infants fed a zinc supplemented formula**. *Am J Clin Nutr* 1976, **29**(10):1114.
52. Arroyave G, Mejia LA, Aguilar JR: **The effect of vitamin A fortification of sugar on the serum vitamin A levels of preschool Guatemalan children: a longitudinal evaluation**. *The American journal of clinical nutrition* 1981, **34**(1):41.
53. Chen K, Li TY, Chen L, Qu P, Liu YX: **Effects of vitamin A, vitamin A plus iron and multiple micronutrient-fortified seasoning powder on preschool children in a suburb of Chongqing, China**. *Journal of nutritional science and vitaminology* 2008, **54**(6):440-447.
54. Mejia LA, Arroyave G: **The effect of vitamin A fortification of sugar on iron metabolism in preschool children in Guatemala**. *The American journal of clinical nutrition* 1982, **36**(1):87.
55. Muhilal P: **Vitamin A-fortified monosodium glutamate and health, growth, and survival of children: a controlled field trial**. *American journal of clinical nutrition (USA)* 1988.
56. Ribaya-Mercado JD, Solomons NW, Medrano Y, Bulux J, Dolnikowski GG, Russell RM, Wallace CB: **Use of the deuterated-retinol-dilution technique to monitor the vitamin A status of Nicaraguan schoolchildren 1 y after initiation of the Nicaraguan national program of sugar fortification with vitamin A**. *The American journal of clinical nutrition* 2004, **80**(5):1291.
57. Solon FS, Klemm RDW, Sanchez L, Darnton-Hill I, Craft NE, Christian P, West KP: **Efficacy of a vitamin A–fortified wheat-flour bun on the vitamin A status of Filipino schoolchildren**. *The American journal of clinical nutrition* 2000, **72**(3):738.
58. Solon FS, Fernandez TL, Latham MC, Popkin BM: **Planning, implementation, and evaluation of a fortification program. Control of vitamin A deficiency in the Philippines**. *Journal of the American Dietetic Association* 1979, **74**(2):112.
59. Zhang X, Chen K, Qu P, Liu YX, Li TY: **Effect of biscuits fortified with different doses of vitamin A on indices of vitamin A status, haemoglobin and physical growth levels of pre-school children in Chongqing**. *Public health nutrition* 2010, **13**(09):1462-1471.
60. Bonjour JP, Carrie AL, Ferrari S, Clavien H, Slosman D, Theintz G, Rizzoli R: **Calcium-enriched foods and bone mass growth in prepubertal girls: a randomized, double-blind, placebo-controlled trial**. *Journal of Clinical Investigation* 1997, **99**(6):1287.

61. Du X, Zhu K, Trube A, Zhang Q, Ma G, Hu X, Fraser DR, Greenfield H: **School-milk intervention trial enhances growth and bone mineral accretion in Chinese girls aged 10–12 years in Beijing**. *British Journal of Nutrition* 2004, **92**(01):159-168.
62. Ganmaa D, Tserendolgor U, Frazier L, Nakamoto E, Jargalsaikhan N, Rich-Edwards J: **Effects of vitamin D fortified milk on vitamin D status in Mongolian school age children**. *Asia Pacific journal of clinical nutrition* 2008, **17**(1):68-71.
63. Graham D, Kira G, Conaglen J, McLennan S, Rush E: **Vitamin D status of Year 3 children and supplementation through schools with fortified milk**. *Public health nutrition* 2009, **12**(12):2329-2334.
64. Ho SC, Guldan GS, Woo J, Yu R, Tse MM, Sham A, Cheng J: **A prospective study of the effects of 1-year calcium-fortified soy milk supplementation on dietary calcium intake and bone health in Chinese adolescent girls aged 14 to 16**. *Osteoporosis International* 2005, **16**(12):1907-1916.
65. Koo WWK, Hockman EM: **Posthospital discharge feeding for preterm infants: effects of standard compared with enriched milk formula on growth, bone mass, and body composition**. *The American journal of clinical nutrition* 2006, **84**(6):1357.
66. Rich-Edwards JW, Ganmaa D, Kleinman K, Sumberzul N, Holick MF, Lkhagvasuren T, Dulguun B, Burke A, Frazier AL: **Randomized trial of fortified milk and supplements to raise 25-hydroxyvitamin D concentrations in schoolchildren in Mongolia**. *The American journal of clinical nutrition* 2011.
67. Lehtonen-Veromaa M, Mottonen T, Leino A, Heinonen OJ, Rautava E, Viikari J: **Prospective study on food fortification with vitamin D among adolescent females in Finland: minor effects**. *British Journal of Nutrition* 2008, **100**(2):418-423.
68. Zhu K, Du X, Cowell CT, Greenfield H, Blades B, Dobbins TA, Zhang Q, Fraser DR: **Effects of school milk intervention on cortical bone accretion and indicators relevant to bone metabolism in Chinese girls aged 10–12 y in Beijing**. *The American journal of clinical nutrition* 2005, **81**(5):1168.
69. Zhu K, Greenfield H, Zhang Q: **Effects of two year's milk supplementation on site-corrected bone mineral density of Chinese girls**. *Asia Pac J Clin Nutr* 2008, **17**(S1):147-150.
70. Grupp SG, Greenberg ML, Ray JG, Busto U, Lanctôt KL, Nulman I, Koren G: **Pediatric cancer rates after universal folic acid flour fortification in Ontario**. *The Journal of Clinical Pharmacology* 2011, **51**(1):60.
71. Wiltshire EJ, Couper JJ: **Improved folate status in children and adolescents during voluntary fortification of food with folate**. *Journal of Paediatrics and Child Health* 2004, **40**(1 2):44-47.
72. Delange F, De Benoist B, Alnwick D: **Risks of Iodine-Induced Hyperthyroidism After Correction of Iodine Deficiency by Iodized Salt***. *Thyroid* 1999, **9**(6):545-556.
73. Guttikonda K, Burgess JR, Hynes K, Boyages S, Byth K, Parameswaran V: **Recurrent iodine deficiency in Tasmania, Australia: a salutary lesson in sustainable iodine prophylaxis and its monitoring**. *Journal of Clinical Endocrinology & Metabolism* 2002, **87**(6):2809.
74. Jooste P, Zimmermann MB: **Progress towards eliminating iodine deficiency in South Africa**. *South African Journal of Clinical Nutrition* 2008, **21**(1):08.
75. Pongpaew P, Tungtrongchitr R, Phonrat B, Supawan V, Schelp EP, Intarakhao C, Mahaweerawat U, Saowakontha S: **Nutritional status of school children in an endemic area of iodine deficiency disorders (IDD) after one year of iodine supplementation**. *Southeast Asian journal of tropical medicine and public health* 1998, **29**:50-57.

76. Yamamah G: **Monitoring Iodine Deficiency of Female Children After Introducing Iodized Salt in El-Farafra Oasis**. 2003.
77. Zimmermann MB, Hess SY, Adou P, Toresanni T, Wegmuller R, Hurrell RF: **Thyroid size and goiter prevalence after introduction of iodized salt: a 5-y prospective study in schoolchildren in Cote d'Ivoire**. *Am J Clin Nutr* 2003, **77**(3):663-667.
78. Zimmermann MB, Jooste PL, Mabapa NS, Mbhenyane X, Schoeman S, Biebinger R, Chaouki N, Bozo M, Grimci L, Bridson J: **Treatment of iodine deficiency in school-age children increases insulin-like growth factor (IGF)-I and IGF binding protein-3 concentrations and improves somatic growth**. *Journal of Clinical Endocrinology & Metabolism* 2006, **92**(2):437.
79. Andersson M, Thankachan P, Muthayya S, Goud RB, Kurpad AV, Hurrell RF, Zimmermann MB: **Dual fortification of salt with iodine and iron: a randomized, double-blind, controlled trial of micronized ferric pyrophosphate and encapsulated ferrous fumarate in southern India**. *The American journal of clinical nutrition* 2008, **88**(5):1378.
80. Nugroho HW, Lestari ED, Salimo H, Dewi M: **Effects of iron and zinc fortified milk supplementation on working memory of underweight poor-urban school children: A randomized double blind controlled trial**. *Paediatrica Indonesiana* 2010, **50**(2):93.
81. Sivakumar B, Nair KM: **Double fortified salt at crossroads**. *Indian Journal of Pediatrics* 2002, **69**(7):617-623.
82. Vinodkumar M, Rajagopalan S, Bhagwat IP, Singh S, Parmar BS, Mishra OP, Upadhyay SS, Bhalia NB, Deshpande SR: **A multicenter community study on the efficacy of double-fortified salt**. *Food &# 38; Nutrition Bulletin* 2007, **28**(1):100-108.
83. Wegmüller R, Camara F, Zimmermann MB, Adou P, Hurrell RF: **Salt dual-fortified with iodine and micronized ground ferric pyrophosphate affects iron status but not hemoglobin in children in Cote d'Ivoire**. *The Journal of nutrition* 2006, **136**(7):1814.
84. Zimmermann MB, Zeder C, Chaouki N, Saad A, Torresani T, Hurrell RF: **Dual fortification of salt with iodine and microencapsulated iron: a randomized, double-blind, controlled trial in Moroccan schoolchildren**. *The American journal of clinical nutrition* 2003, **77**(2):425.
85. Zimmermann MB, Wegmueller R, Zeder C, Chaouki N, Rohner F, Saïssi M, Torresani T, Hurrell RF: **Dual fortification of salt with iodine and micronized ferric pyrophosphate: a randomized, double-blind, controlled trial**. *The American journal of clinical nutrition* 2004, **80**(4):952.
86. Zimmermann MB, Zeder C, Chaouki N, Torresani T, Saad A, Hurrell RF: **Addition of microencapsulated iron to iodized salt improves the efficacy of iodine in goitrous, iron-deficient children: a randomized, double-blind, controlled trial**. *European Journal of Endocrinology* 2002, **147**(6):747.
87. Zlotkin S, Arthur P, Antwi KY, Yeung G: **Treatment of anemia with microencapsulated ferrous fumarate plus ascorbic acid supplied as sprinkles to complementary (weaning) foods**. *The American journal of clinical nutrition* 2001, **74**(6):791.
88. Zlotkin S, Arthur P, Schauer C, Antwi KY, Yeung G, Piekarz A: **Home-fortification with iron and zinc sprinkles or iron sprinkles alone successfully treats anemia in infants and young children**. *The Journal of nutrition* 2003, **133**(4):1075.
89. Chen K, Zhang X, Li T, Chen L, Wei X, Qu P, Liu Y: **Effect of vitamin A, vitamin A plus iron and multiple micronutrient-fortified seasoning powder on infectious morbidity of preschool children**. *Nutrition* 2011.
90. Chen K, Li TY, Chen L, Qu P, Liu YX: **Effects of vitamin A, vitamin A plus iron and multiple micronutrient-fortified seasoning powder on preschool children in a suburb of Chongqing, China**. *Journal of nutritional science and vitaminology* 2008, **54**(6):440-447.
91. Faber M, Kvalsvig JD, Lombard CJ, Benade AJ: **Effect of a fortified maize-meal porridge on anemia, micronutrient status, and motor development of infants**. *The American journal of clinical nutrition* 2005, **82**(5):1032.

92. Gershoff SN, McGandy RB, Suttapreyasri D, Promkutkao C, Nondasuta A, Pisolyabutra U, Tantiwongse P, Viravaidhaya V: **Nutrition studied in Thailand. II. Effects of fortification of rice with lysine, threonine, thiamin, riboflavin, vitamin A, and iron on preschool children**. *The American journal of clinical nutrition* 1977, **30**(7):1185.
93. Gibson RS, Kafwembe E, Mwanza S, Gosset L, Bailey KB, Mullen A, Baisley K, Filteau S: **A Micronutrient-Fortified Food Enhances Iron and Selenium Status of Zambian Infants but Has Limited Efficacy on Zinc**. *The Journal of nutrition* 2011, **141**(5):935.
94. Goyle A, Prakash S: **Effect of supplementation of micronutrient fortified biscuits on haemoglobin and serum iron levels of adolescent girls from Jaipur city, India**. *Nutrition & Food Science* 2010, **40**(5):477-484.
95. Kumar MV, Rajagopalan S: **Multiple micronutrient fortification of salt and its effect on cognition in Chennai school children**. *Asia Pac J Clin Nutr* 2007, **16**(3):505-511.
96. Kuusipalo H, Maleta K, Briend A, Manary M, Ashorn P: **Growth and change in blood haemoglobin concentration among underweight Malawian infants receiving fortified spreads for 12 weeks: a preliminary trial**. *Journal of pediatric gastroenterology and nutrition* 2006, **43**(4):525.
97. Lartey A, Manu A, Brown KH, Peerson JM, Dewey KG: **A randomized, community-based trial of the effects of improved, centrally processed complementary foods on growth and micronutrient status of Ghanaian infants from 6 to 12 mo of age**. *The American journal of clinical nutrition* 1999, **70**(3):391.
98. Layrisse M, Chaves JF: **Early response to the effect of iron fortification in the Venezuelan population**. *The American journal of clinical nutrition* 1996, **64**(6):903.
99. Lein, Shigeru Y, te Biesebeke R: **Impact of milk consumption on performance and health of primary school children in rural Vietnam**. *Asia Pac J Clin Nutr* 2009, **18**(3):326-334.
100. Lin J, Lee I: **Plasma folate, vitamin B-6, vitamin B-12, and risk of breast cancer in women**. *The American journal of clinical nutrition* 2008, **87**(3):734.
101. Liu D, Bates CJ, Yin TA, Wang XB, Lu C: **Nutritional efficacy of a fortified weaning rusk in a rural area near Beijing [published erratum appears in Am J Clin Nutr 1993 Oct; 58 (4): 582]**. *The American journal of clinical nutrition* 1993, **57**(4):506.
102. Lopriore C, Guidoum Y, Briend A, Branca F: **Spread fortified with vitamins and minerals induces catch-up growth and eradicates severe anemia in stunted refugee children aged 3–6 y**. *The American journal of clinical nutrition* 2004, **80**(4):973.
103. Loui A, Raab A, Wagner M, Weigel H, Grüters-Kieslich A, Brätter P, Obladen M: **Nutrition of very low birth weight infants fed human milk with or without supplemental trace elements: a randomized controlled trial**. *Journal of pediatric gastroenterology and nutrition* 2004, **39**(4):346.
104. Lucas A, Fewtrell MS, Morley R, Lucas PJ, Baker BA, Lister G, Bishop NJ: **Randomized outcome trial of human milk fortification and developmental outcome in preterm infants**. *The American journal of clinical nutrition* 1996, **64**(2):142.
105. Lutter CK, Rodríguez A, Fuenmayor G, Avila L, Sempertegui F, Escobar J: **Growth and micronutrient status in children receiving a fortified complementary food**. *The Journal of nutrition* 2007, **138**(2):379.
106. Manger MS, McKenzie JE, Winichagoon P, Gray A, Chavasit V, Pongcharoen T, Gowachirapant S, Ryan B, Wasantwisut E, Gibson RS: **A micronutrient-fortified seasoning powder reduces morbidity and improves short-term cognitive function, but has no effect on**

anthropometric measures in primary school children in northeast Thailand: a randomized controlled trial. *The American journal of clinical nutrition* 2008, **87**(6):1715.

107. Manno D, Kowa PK, Bwalya HK, Siame J, Grantham-McGregor S, Baisley K, De Stavola BL, Jaffar S, Filteau S: **Rich micronutrient fortification of locally produced infant food does not improve mental and motor development of Zambian infants: a randomised controlled trial**. *British Journal of Nutrition* 2011, **1**(1):1-11.
108. Morgan EJ, Heath ALM, Szymlek-Gay EA, Gibson RS, Gray AR, Bailey KB, Ferguson EL: **Red Meat and a Fortified Manufactured Toddler Milk Drink Increase Dietary Zinc Intakes without Affecting Zinc Status of New Zealand Toddlers**. *The Journal of nutrition* 2010, **140**(12):2221.
109. Nesamvuni AE, Vorster HH, Margetts BM, Kruger A: **Fortification of maize meal improved the nutritional status of 1–3-year-old African children**. *Public health nutrition* 2005, **8**(05):461-467.
110. Nga TT, Winichagoon P, Dijkhuizen MA, Khan NC, Wasantwisut E, Furr H, Wieringa FT: **Multi-Micronutrient–Fortified Biscuits Decreased Prevalence of Anemia and Improved Micronutrient Status and Effectiveness of Deworming in Rural Vietnamese School Children**. *The Journal of nutrition* 2009, **139**(5):1013.
111. Osei AK, Rosenberg IH, Houser RF, Bulusu S, Mathews M, Hamer DH: **Community-level micronutrient fortification of school lunch meals improved vitamin A, folate, and iron status of schoolchildren in Himalayan villages of India**. *The Journal of nutrition* 2010, **140**(6):1146.
112. Ouédraogo HZ, Traoré T, Zèba AN, Dramaix-Wilmet M, Hennart P, Donnen P: **Effect of an improved local ingredient-based complementary food fortified or not with iron and selected multiple micronutrients on Hb concentration**. *Public health nutrition* 2006, **1**(-1):1-8.
113. Van Phu P, Van Hoan N, Salvignol B, Treche S, Wieringa FT, Khan NC, Tuong PD, Berger J: **Complementary Foods Fortified with Micronutrients Prevent Iron Deficiency and Anemia in Vietnamese infants**. *The Journal of nutrition* 2010, **140**(12):2241.
114. Rivera JA, Shamah T, Villalpando S, Monterrubio E: **Effectiveness of a large-scale iron-fortified milk distribution program on anemia and iron deficiency in low-income young children in Mexico**. *The American journal of clinical nutrition* 2010, **91**(2):431.
115. Sazawal S, Dhingra U, Dhingra P, Hiremath G, Sarkar A, Dutta A, Menon VP, Black RE: **Micronutrient fortified milk improves iron status, anemia and growth among children 1–4 years: a double masked, randomized, controlled trial**. *PloS one* 2010, **5**(8):e12167.
116. Seal A, Kafwembe E, Kassim IAR, Hong M, Wesley A, Wood J, Abdalla F, van den Briel T: **Maize meal fortification is associated with improved vitamin A and iron status in adolescents and reduced childhood anaemia in a food aid-dependent refugee population**. *Public health nutrition* 2007, **11**(07):720-728.
117. Stekel A, Olivares M, Cayazzo M, Chadud P, Llaguno S, Pizarro F: **Prevention of iron deficiency by milk fortification. II. A field trial with a full-fat acidified milk**. *The American journal of clinical nutrition* 1988, **47**(2):265.
118. Van Stuijvenberg ME, Dhansay MA, Smuts CM, Lombard CJ, Jogessar VB, Benadé AJ: **Long-term evaluation of a micronutrient-fortified biscuit used for addressing micronutrient deficiencies in primary school children**. *Public health nutrition* 2001, **4**(6):1201-1210.
119. Torrejón CS, Castillo-Durán C, Hertrampf ED, Ruz M: **Zinc and iron nutrition in Chilean children fed fortified milk provided by the complementary national food program* 1**. *Nutrition* 2004, **20**(2):177-180.

120. Varma JL, Das S, Sankar R, Mannar MGV, Levinson FJ, Hamer DH: **Community-level micronutrient fortification of a food supplement in India: a controlled trial in preschool children aged 36-66 mo**. *The American journal of clinical nutrition* 2007, **85**(4):1127.
121. Villalpando S, Shamah T, Rivera JA, Lara Y, Monterrubio E: **Fortifying milk with ferrous gluconate and zinc oxide in a public nutrition program reduced the prevalence of anemia in toddlers**. *The Journal of nutrition* 2006, **136**(10):2633.
122. Winichagoon P, McKenzie JE, Chavasit V, Pongcharoen T, Gowachirapant S, Boonpraderm A, Manger MS, Bailey KB, Wasantwisut E, Gibson RS: **A multimicronutrient-fortified seasoning powder enhances the hemoglobin, zinc, and iodine status of primary school children in North East Thailand: a randomized controlled trial of efficacy**. *The Journal of nutrition* 2006, **136**(6):1617.
123. Andersson M, Theis W, Zimmermann MB, Foman JT, Jakel M, Duchateau GS, Frenken LG, Hurrell RF: **Random serial sampling to evaluate efficacy of iron fortification: a randomized controlled trial of margarine fortification with ferric pyrophosphate or sodium iron edetate**. *Am J Clin Nutr* 2010, **92**(5):1094-1104.
124. Biebinger R, Zimmermann MB, Al-Hooti SN, Al-Hamed N, Al-Salem E, Zafar T, Kabir Y: **Efficacy of wheat-based biscuits fortified with microcapsules containing ferrous sulfate and potassium iodate or a new hydrogen-reduced elemental iron: a randomised, double-blind, controlled trial in Kuwaiti women**. *British Journal of Nutrition* 2009, **102**(09):1362-1369.
125. Blanco-Rojo R, Perez-Granados AM, Toxqui L, Zazo P, de la Piedra C, Vaquero MP: **Relationship between vitamin D deficiency, bone remodelling and iron status in iron-deficient young women consuming an iron-fortified food**. *European journal of nutrition* 2012.
126. Blanco-Rojo R, Pérez-Granados AM, Toxqui L, González-Vizcayno C, Delgado MA, Vaquero MP: **Efficacy of a microencapsulated iron pyrophosphate-fortified fruit juice: a randomised, double-blind, placebo-controlled study in Spanish iron-deficient women**. *British Journal of Nutrition* 2011, **105**(11):1652-1659.
127. Hoa PT, Khan NC, van Beusekom C, Gross R, Conde WL, Khoi HD: **Milk fortified with iron or iron supplementation to improve nutritional status of pregnant women: An intervention trial from rural Vietnam**. *Food & Nutrition Bulletin* 2005, **26**(1):32-38.
128. Hotz C, Porcayo M, Onofre G, GarcÃ-a-Guerra A, Elliott T, Jankowski S, Greiner T: **Efficacy of iron-fortified Ultra Rice in improving the iron status of women in Mexico**. *Food &# 38; Nutrition Bulletin* 2008, **29**(2):140-149.
129. Karl JP, Lieberman HR, Cable SJ, Williams KW, Young AJ, McClung JP: **Randomized, double-blind, placebo-controlled trial of an iron-fortified food product in female soldiers during military training: relations between iron status, serum hepcidin, and inflammation**. *American Journal of Clinical Nutrition* 2010, **92**(1):93.
130. Sadighi J, Sheikholeslam R, Mohammad K, Pouraram H, Abdollahi Z, Samadpour K, Kolahdooz F, Naghavi M: **Flour fortification with iron: a mid-term evaluation**. *Public health* 2008, **122**(3):313-321.
131. Sadighi J, Mohammad K, Sheikholeslam R, Amirkhani MA, Torabi P, Salehi F, Abdolahi Z: **Anaemia control: lessons from the flour fortification programme**. *Public health* 2009, **123**(12):794-799.
132. Thuy PV, Berger J, Davidsson L, Khan NC, Lam NT, Cook JD, Hurrell RF, Khoi HH: **Regular consumption of NaFeEDTA-fortified fish sauce improves iron status and reduces the prevalence of anemia in anemic Vietnamese women**. *American Journal of Clinical Nutrition* 2003, **78**(2):284-290.
133. Van Thuy P, Berger J, Nakanishi Y, Khan NC, Lynch S, Dixon P: **The use of NaFeEDTA-fortified fish sauce is an effective tool for controlling iron deficiency in women of childbearing age in rural Vietnam**. *Journal of Nutrition* 2005, **135**(11):2596.

134. Zimmermann MB, Winichagoon P, Gowachirapant S, Hess SY, Harrington M, Chavasit V, Lynch SR, Hurrell RF: **Comparison of the efficacy of wheat-based snacks fortified with ferrous sulfate, electrolytic iron, or hydrogen-reduced elemental iron: randomized, double-blind, controlled trial in Thai women**. *Am J Clin Nutr* 2005, **82**(6):1276-1282.
135. Badii A, Nekouei N, Fazilati M, Shahedi M, Badiei S: **Effect of Consuming Zinc-fortified Bread on Serum Zinc and Iron Status of Zinc-deficient Women: A Double Blind, Randomized Clinical Trial**. *International journal of preventive medicine* 2012, **3**(Suppl 1):S124-130.
136. Alasfoor D, Elsayed DRMK, Mohammed ALIJ: **SPINA BIFIDA AND BIRTH OUTCOME BEFORE AND AFTER FORTIFICATION OF WHITE FLOUR WITH IRON AND FOLIC ACID IN OMAN**. 2010.
137. Amarin ZO, Obeidat AZ: **Effect of folic acid fortification on the incidence of neural tube defects**. *Paediatric and perinatal epidemiology* 2010, **24**(4):349-351.
138. Bar-Oz B, Koren G, Nguyen P, Kapur BM: **Folate fortification and supplementation--Are we there yet?** *Reproductive Toxicology* 2008, **25**(4):408-412.
139. Bol KA, Collins JS, Kirby RS: **Survival of infants with neural tube defects in the presence of folic acid fortification**. *Pediatrics* 2006, **117**(3):803.
140. Boulet SL, Yang Q, Mai C, Kirby RS, Collins JS, Robbins JM, Meyer R, Canfield MA, Mulinare J: **Trends in the postfortification prevalence of spina bifida and anencephaly in the United States**. *Birth Defects Research Part A: Clinical and Molecular Teratology* 2008, **82**(7):527-532.
141. Canfield MA, Mai CT, Kirby RS, Pearson K, Devine O, Mulinare J: **Changes in the birth prevalence of selected birth defects after grain fortification with folic acid in the United States: findings from a multi-state population-based study**. *Birth Defects Research (Part A): Clinical and Molecular Teratology* 2005, **73**:679-689.
142. Chen LT, Rivera MA: **The Costa Rican experience: reduction of neural tube defects following food fortification programs**. *Nutrition Reviews* 2004, **62**:S40-S43.
143. Chen BH, Carmichael SL, Selvin S, Abrams B, Shaw GM: **NTD prevalences in central California before and after folic acid fortification**. *Birth Defects Research Part A: Clinical and Molecular Teratology* 2008, **82**(8):547-552.
144. Hertrampf E, Cortés F, Erickson JD, Cayazzo M, Freire W, Bailey LB, Howson C, Kauwell G, Pfeiffer C: **Consumption of folic acid–fortified bread improves folate status in women of reproductive age in Chile**. *The Journal of nutrition* 2003, **133**(10):3166.
145. Honein MA, Paulozzi LJ, Mathews TJ, Erickson JD, Wong LYC: **Impact of folic acid fortification of the US food supply on the occurrence of neural tube defects**. *JAMA: The Journal of the American Medical Association* 2001, **285**(23):2981.
146. Ionescu-Ittu R, Marelli AJ, Mackie AS, Pilote L: **Prevalence of severe congenital heart disease after folic acid fortification of grain products: time trend analysis in Quebec, Canada**. *BMJ: British Medical Journal* 2009, **338**.
147. Kucik J, Correa A: **Trends in twinning rates in metropolitan Atlanta before and after folic acid fortification**. *Journal of reproductive medicine* 2004, **49**(9):707-712.
148. Lawrence JM, Watkins ML, Chiu V, Erickson JD, Petitti DB: **Food fortification with folic acid and rate of multiple births, 1994–2000**. *Birth Defects Research Part A: Clinical and Molecular Teratology* 2004, **70**(12):948-952.

149. Liu S, West R, Randell E, Longerich L, O'Connor K, Scott H, Crowley M, Lam A, Prabhakaran V, McCourt C: **A comprehensive evaluation of food fortification with folic acid for the primary prevention of neural tube defects**. *BMC pregnancy and childbirth* 2004, **4**(1):20.
150. López Camelo JS, Orioli IM, Dutra MG, Nazer Herrera J, Rivera N, Ojeda ME, Canessa A, Wettig E, Fontannaz AM, Mellado C: **Reduction of birth prevalence rates of neural tube defects after folic acid fortification in Chile**. *American Journal of Medical Genetics Part A* 2005, **135**(2):120-125.
151. Modjadji SE, Alberts M, Mamabolo RL: **Folate and iron status of South African non-pregnant rural women of childbearing age, before and after fortification of foods**. *South African Journal of Clinical Nutrition* 2008, **20**(3):89-93.
152. Murphy M, Whiteman D, Stone D, Botting B, Schorah C, Wild J: **Dietary folate and the prevalence of neural tube defects in the British Isles: the past two decades**. *BJOG: An International Journal of Obstetrics & Gynaecology* 2000, **107**(7):885-889.
153. Orioli IM, Lima do Nascimento R, López Camelo JS, Castilla EE: **Effects of folic acid fortification on spina bifida prevalence in Brazil**. *Birth Defects Research Part A: Clinical and Molecular Teratology* 2011.
154. Pacheco SS, Braga C, Souza AI, Figueiroa JN: **Effects of folic acid fortification on the prevalence of neural tube defects**. *Revista de Saúde Pública* 2009, **43**(4):565-571.
155. Persad VL, Van den Hof MC, Dubé JM, Zimmer P: **Incidence of open neural tube defects in Nova Scotia after folic acid fortification**. *Canadian Medical Association Journal* 2002, **167**(3):241.
156. Ray JG: **Folic acid food fortification in Canada**. *Nutrition Reviews* 2004, **62**:S35-S39.
157. Ray JG, Meier C, Vermeulen MJ, Cole DEC, Wyatt PR: **Prevalence of trisomy 21 following folic acid food fortification**. *American Journal of Medical Genetics Part A* 2003, **120**(3):309-313.
158. Robbins JM, Tilford JM, Bird TM, Cleves MA, Reading JA, Hobbs CA: **Hospitalizations of newborns with folate-sensitive birth defects before and after fortification of foods with folic acid**. *Pediatrics* 2006, **118**(3):906.
159. Safdar OY, Al-Dabbagh AA, AbuElieneen WA, Kari JA: **Decline in the incidence of neural tube defects after the national fortification of flour (1997-2005)**. *Saudi medical journal* 2007, **28**(8):1227.
160. Sayed AR, Bourne D, Pattinson R, Nixon J, Henderson B: **Decline in the prevalence of neural tube defects following folic acid fortification and its cost benefit in South Africa**. *Birth Defects Research Part A: Clinical and Molecular Teratology* 2008, **82**(4):211-216.
161. Shaw GM, Carmichael SL, Nelson V, Selvin S, Schaffer DM: **Food fortification with folic acid and twinning among California infants**. *American Journal of Medical Genetics Part A* 2003, **119**(2):137-140.
162. Signore C, Mills JL, Cox C, Trumble AC: **Effects of folic acid fortification on twin gestation rates**. *Obstetrics & Gynecology* 2005, **105**(4):757.
163. Simmons CJ, Mosley BS, Fulton Bond CA, Hobbs CA: **Birth defects in Arkansas: is folic acid fortification making a difference?** *Birth Defects Research Part A: Clinical and Molecular Teratology* 2004, **70**(9):559-564.
164. Waller DK, Tita ATN, Annegers JF: **Rates of twinning before and after fortification of foods in the US with folic acid, Texas, 1996 to 1998**. *Paediatric and perinatal epidemiology* 2003, **17**(4):378-383.
165. Williams LJ, Mai CT, Edmonds LD, Shaw GM, Kirby RS, Hobbs CA, Sever LE, Miller LA, Meaney FJ, Levitt M: **Prevalence of spina bifida and anencephaly during the transition to mandatory folic acid fortification in the United States**. *Teratology* 2002, **66**(1):33-39.

166. Adolphi B, Scholz-Ahrens KE, de Vrese M, Açil Y, Laue C, Schrezenmeir J: **Short-term effect of bedtime consumption of fermented milk supplemented with calcium, inulin-type fructans and caseinphosphopeptides on bone metabolism in healthy, postmenopausal women**. *European journal of nutrition* 2009, **48**(1):45-53.
167. Bonjour JP, Benoit V, Pourchaire O, Ferry M, Rousseau B, Souberbielle JC: **Inhibition of markers of bone resorption by consumption of vitamin D and calcium-fortified soft plain cheese by institutionalised elderly women**. *British Journal of Nutrition* 2009, **102**(07):962-966.
168. Chee WSS, Suriah AR, Chan SP, Zaitun Y, Chan YM: **The effect of milk supplementation on bone mineral density in postmenopausal Chinese women in Malaysia**. *Osteoporosis International* 2003, **14**(10):828-834.
169. Ferrar L, van der Hee RM, Berry M, Watson C, Miret S, Wilkinson J, Bradburn M, Eastell R: **Effects of calcium-fortified ice cream on markers of bone health**. *Osteoporosis International* 2010:1-11.
170. Green TJ, Skeaff CM, Rockell JE: **Milk fortified with the current adequate intake for vitamin D (5 g) increases serum 25-hydroxyvitamin D compared to control milk but is not sufficient to prevent a seasonal decline in young women**. *Asia Pacific journal of clinical nutrition* 2010, **19**(2):195-199.
171. Kruger MC, Booth CL, Coad J, Schollum LM, Kuhn-Sherlock B, Shearer MJ: **Effect of calcium fortified milk supplementation with or without vitamin K on biochemical markers of bone turnover in premenopausal women**. *Nutrition* 2006, **22**(11-12):1120-1128.
172. Kruger MC, Schollum LM, Kuhn-Sherlock B, Hestiantoro A, Wijanto P, Li-Yu J, Agdeppa I, Todd JM, Eastell R: **The effect of a fortified milk drink on vitamin D status and bone turnover in post-menopausal women from South East Asia**. *Bone* 2010, **46**(3):759-767.
173. Lau EM, Woo J, Lam V, Hong A: **Milk supplementation of the diet of postmenopausal Chinese women on a low calcium intake retards bone loss**. *Journal of bone and mineral research : the official journal of the American Society for Bone and Mineral Research* 2001, **16**(9):1704-1709.
174. Manios Y, Moschonis G, Panagiotakos DB, Farajian P, Trovas G, Lyritis GP: **Changes in biochemical indices of bone metabolism in post menopausal women following a dietary intervention with fortified dairy products**. *Journal of Human Nutrition and Dietetics* 2009, **22**(2):156-165.
175. Moschonis G, Manios Y: **Skeletal site-dependent response of bone mineral density and quantitative ultrasound parameters following a 12-month dietary intervention using dairy products fortified with calcium and vitamin D: the Postmenopausal Health Study**. *British Journal of Nutrition* 2006, **96**(06):1140-1148.
176. Natri AM, Salo P, Vikstedt T, Palssa A, Huttunen M, Kärkkäinen MUM, Salovaara H, Piironen V, Jakobsen J, Lamberg-Allardt CJ: **Bread fortified with cholecalciferol increases the serum 25-hydroxyvitamin D concentration in women as effectively as a cholecalciferol supplement**. *The Journal of nutrition* 2006, **136**(1):123.
177. Recker RR, Heaney RP: **The effect of milk supplements on calcium metabolism, bone metabolism and calcium balance**. *The American journal of clinical nutrition* 1985, **41**(2):254.
178. Storm D, Porter REES, Musgrave K, Vereault D, Patton C, Kessenich C, Mohan S, Chen T, Holick MF, Rosen CJ: **Calcium supplementation prevents seasonal bone loss and changes in biochemical markers of bone turnover in elderly New England women: a randomized placebo-controlled trial**. *Journal of Clinical Endocrinology & Metabolism* 1998, **83**(11):3817.

179. Tenta R, Moschonis G, Koutsilieris M, Manios Y: **Calcium and vitamin D supplementation through fortified dairy products counterbalances seasonal variations of bone metabolism indices: the Postmenopausal Health Study**. *European journal of nutrition* 2010:1-9.
180. Burgess JR, Seal JA, Stilwell GM, Reynolds PJ, Taylor ER, Parameswaran V: **A case for universal salt iodisation to correct iodine deficiency in pregnancy: another salutary lesson from Tasmania**. *Medical journal of Australia* 2007, **186**(11):574.
181. Camargo RYA, Tomimori EK, Neves SC, Gs R: **Thyroid and the environment: exposure to excessive nutritional iodine increases the prevalence of thyroid disorders in Sao Paulo, Brazil**. *European Journal of Endocrinology* 2008, **159**(3):293.
182. Carle A, Laurberg P, Pedersen IB, Knudsen N, Perrild H, Ovesen L, Rasmussen LB, Jorgensen T: **Epidemiology of subtypes of hypothyroidism in Denmark**. *European Journal of Endocrinology* 2006, **154**(1):21.
183. Cerqueira C, Knudsen N, Ovesen L, Perrild H, Rasmussen LB, Laurberg P, Jorgensen T: **Association of Iodine Fortification with Incident Use of Antithyroid Medication--A Danish Nationwide Study**. *Journal of Clinical Endocrinology & Metabolism* 2009, **94**(7):2400.
184. Cerqueira C, Knudsen N, Ovesen L, Laurberg P, Perrild H, Rasmussen LB, Jørgensen T: **Doubling in the use of thyroid hormone replacement therapy in Denmark: association to iodization of salt?** *European Journal of Epidemiology* 2011:1-7.
185. Laurberg P, Jorgensen T, Perrild H, Ovesen L, Knudsen N, Pedersen IB, Rasmussen LB, Carle A, Vejbjerg P: **The Danish investigation on iodine intake and thyroid disease, DanThyr: status and perspectives**. *European Journal of Endocrinology* 2006, **155**(2):219.
186. Moleti M, Lo Presti VP, Campolo MC, Mattina F, Galletti M, Mandolfino M, Violi MA, Giorgianni G, De Domenico D, Trimarchi F: **Iodine prophylaxis using iodized salt and risk of maternal thyroid failure in conditions of mild iodine deficiency**. *Journal of Clinical Endocrinology & Metabolism* 2008, **93**(7):2616.
187. Moleti M, Di Bella B, Giorgianni G, Mancuso A, De Vivo A, Alibrandi A, Trimarchi F, Vermiglio F: **MATERNAL THYROID FUNCTION IN DIFFERENT CONDITIONS OF IODINE NUTRITION IN PREGNANT WOMEN EXPOSED TO MILD MODERATE IODINE DEFICIENCY: AN OBSERVATIONAL STUDY**. *Clinical endocrinology* 2011.
188. Bülow Pedersen I, Laurberg P, Knudsen N, Jørgensen T, Perrild H, Ovesen L, Rasmussen LB: **An increased incidence of overt hypothyroidism after iodine fortification of salt in Denmark: a prospective population study**. *Journal of Clinical Endocrinology & Metabolism* 2007, **92**(8):3122.
189. Bülow PI, Knudsen N, Carlé A, Vejbjerg P, Jørgensen T, Perrild H, Ovesen L, Banke RL, Laurberg P: **A cautious iodization program bringing iodine intake to a low recommended level is associated with an increase in the prevalence of thyroid autoantibodies in the population**. *Clinical endocrinology*.
190. Rasmussen LB, Carlé A, Jørgensen T, Knudsen N, Laurberg P, Pedersen IB, Perrild H, Vejbjerg P, Ovesen L: **Iodine intake before and after mandatory iodization in Denmark: results from the Danish Investigation of Iodine Intake and Thyroid Diseases (DanThyr) study**. *British Journal of Nutrition* 2008, **100**(01):166-173.
191. Seal JA, Doyle Z, Burgess JR, Taylor R, Cameron AR: **Iodine status of Tasmanians following voluntary fortification of bread with iodine**. *Medical journal of Australia* 2007, **186**(2):69.
192. Sebotsa MLD, Dannhauser A, Jooste PL, Joubert G: **Iodine status as determined by urinary iodine excretion in Lesotho two years after introducing legislation on universal salt iodization**. *Nutrition* 2005, **21**(1):20-24.

193. Sultanalieva RB, Mamutova S, van der Haar F: **The current salt iodization strategy in Kyrgyzstan ensures sufficient iodine nutrition among school-age children but not pregnant women**. *Public health nutrition* 2010, **13**(05):623-630.
194. Vejbjerg P, Knudsen N, Perrild H, Carle A, Laurberg P, Pedersen IB, Rasmussen LB, Ovesen L, Jorgensen T: **Effect of a mandatory iodization program on thyroid gland volume based on individuals' age, gender, and preceding severity of dietary iodine deficiency: a prospective, population-based study**. *Journal of Clinical Endocrinology & Metabolism* 2007, **92**(4):1397.
195. Vejbjerg P, Knudsen N, Perrild H, Laurberg P, Carlé A, Pedersen IB, Rasmussen LB, Ovesen L, Jørgensen T: **Lower prevalence of mild hyperthyroidism related to a higher iodine intake in the population: prospective study of a mandatory iodization programme**. *Clinical endocrinology* 2009, **71**(3):440-445.
196. Viteri FE, Alvarez E, Batres R, Torun B, Pineda O, Mejia LA, Sylvi J: **Fortification of sugar with iron sodium ethylenediaminotetraacetate (FeNaEDTA) improves iron status in semirural Guatemalan populations**. *Am J Clin Nutr* 1995, **61**(5):1153-1163.
197. Adu-Afarwuah S, Lartey A, Brown KH, Zlotkin S, Briend A, Dewey KG: **Randomized comparison of 3 types of micronutrient supplements for home fortification of complementary foods in Ghana: effects on growth and motor development**. *The American journal of clinical nutrition* 2007, **86**(2):412.
198. Huo J, Sun J, Huang J, Li W, Wang L, Selenje L, Gleason GR, Yu X: **The effectiveness of fortified flour on micro-nutrient status in rural female adults in China**. *Asia Pacific journal of clinical nutrition* 2011, **20**(1):118.
199. Mardones F, Urrutia MT, Villarroel L, Rioseco A, Castillo O, Rozowski J, Tapia JL, Bastias G, Bacallao J, Rojas I: **Effects of a dairy product fortified with multiple micronutrients and omega-3 fatty acids on birth weight and gestation duration in pregnant Chilean women**. *Public health nutrition* 2008, **11**(1):30-40.
200. Tapola NS, Karvonen HM, Niskanen LK, Sarkkinen ES: **Mineral water fortified with folic acid, vitamins B6, B12, D and calcium improves folate status and decreases plasma homocysteine concentration in men and women**. *European Journal of Clinical Nutrition* 2004, **58**(2):376-385.
201. Tucker KL, Olson B, Bakun P, Dallal GE, Selhub J, Rosenberg IH: **Breakfast cereal fortified with folic acid, vitamin B-6, and vitamin B-12 increases vitamin concentrations and reduces homocysteine concentrations: a randomized trial**. *The American journal of clinical nutrition* 2004, **79**(5):805.
