# Supplementary material for: Micronutrient fortification of food and its impact on woman and child health: a systematic review
Source: Syst Rev. 2013 Aug 23;2:67. doi: 10.1186/2046-4053-2-67 (PMC3765883; doi:10.1186/2046-4053-2-67)

Figure S1 Forest plot: Effect of Iron fortification on serum hemoglobin levels in children

Study or Subgroup	Fortification Mean	Fortification SD	Fortification Total	Control Mean	Control SD	Control Total	Weight	Std. Mean Difference IV, Random, 95% CI
**1.16.1 Healthy**								
Abizari 2012	120	11	109	117	11	115	3.1%	0.27 [0.01, 0.54]
Andang'o Electrolyte Fe	112.2	9.9	127	115.7	9.7	64	3.0%	-0.35 [-0.66, -0.05]
Andang'o NaFeEDTA	117.2	8.5	121	115.7	9.7	64	3.0%	0.17 [-0.14, 0.47]
Arcanjo 2010	11.4	1	77	11	0.7	77	3.0%	0.46 [0.14, 0.78]
Bagni 2009	11.75	1.16	180	11.51	1.16	174	3.1%	0.21 [-0.00, 0.42]
Barbosa 2012	126	11	88	123	11	85	3.0%	0.27 [-0.03, 0.57]
Chen 2005	118.2	9.2	202	114	8.9	126	3.1%	0.46 [0.24, 0.69]
Daly 1996	123	7	41	115	13	43	2.8%	0.75 [0.31, 1.20]
de Almeida 2005	11.54	1.35	74	11.95	1.22	76	3.0%	-0.32 [-0.64, 0.00]
Gill 1997	121.5	13.26	192	117.7	11.22	60	3.0%	0.30 [0.00, 0.59]
Haschke 1988	125	7	43	122	7	45	2.8%	0.42 [0.00, 0.85]
Levi Fe Gluconate 2008	120.4	9.6	135	123.6	10.8	67	3.0%	-0.32 [-0.61, -0.02]
Levi Fe Sulphate 2008	120.2	9.9	136	123.6	10	67	3.0%	-0.34 [-0.64, -0.05]
Longfils 2008	109.8	8.7	93	104.9	11.3	44	2.9%	0.51 [0.14, 0.87]
Marsh 1959	12.69	0.95	46	10.46	1.43	39	2.7%	1.85 [1.34, 2.36]
Moffatt 1994	118.6	5.7	78	115.1	5.7	76	3.0%	0.61 [0.29, 0.93]
Morley 1999	126	11	40	120	11	32	2.8%	0.54 [0.07, 1.01]
Nogueira 2012 (2)	11.5	0.8	60	11.2	0.73	60	2.9%	0.39 [0.03, 0.75]
Nogueria 2012 (1)	116.7	0.96	96	113.6	2.1	92	3.0%	1.90 [1.56, 2.25]
Rim 2008	118	11.7	107	110	11.1	104	3.0%	0.70 [0.42, 0.98]
Sari 2001	120.8	7.8	57	115.3	7.9	60	2.9%	0.70 [0.32, 1.07]
Stevens 1995	122	6.1	24	119.7	10.1	26	2.6%	0.27 [-0.29, 0.83]
Szymlek-Gay 2009	121.6	3.1	41	120.2	2.6	81	2.9%	0.50 [0.12, 0.88]
Virtanen 2001	121	7.5	20	121	6.9	16	2.4%	0.00 [-0.66, 0.66]
Walter (2) 1993	126	1	73	121	1.6	64	2.6%	3.78 [3.22, 4.35]
Walter 1993 (1) Boys	142.8	8.5	245	139	8.4	248	3.1%	0.45 [0.27, 0.63]
Walter 1993 (1) Girls	140.4	8.3	209	138.2	8.5	225	3.1%	0.26 [0.07, 0.45]
Zimmerman 2010	107.1	9.4	69	106.6	9.7	70	3.0%	0.05 [-0.28, 0.38]
**Subtotal (95% CI)**			**2783**			**2300**	**82.0%**	**0.50 [0.28, 0.71]**
Heterogeneity: Tau² = 0.30; Chi² = 358.60, df = 27 (P < 0.00001); I² = 92%								
Test for overall effect: Z = 4.52 (P < 0.00001)								
**1.16.2 Deficient**								
Huo 2002	137.8	9	159	118.5	4.7	81	3.0%	2.46 [2.11, 2.81]
Huong 2006	17.8	7.6	72	14.5	8.5	73	3.0%	0.41 [0.08, 0.74]
Moretti 2006	119	9	80	116	11	90	3.0%	0.30 [-0.01, 0.60]
Muthayya 2012	129	11	185	123	11	193	3.1%	0.54 [0.34, 0.75]
Schu¨mann 2005	120.8	7.8	57	115.3	7.9	60	2.9%	0.70 [0.32, 1.07]
Zimmerman 2006	117	8	66	114	10	68	3.0%	0.33 [-0.01, 0.67]
**Subtotal (95% CI)**			**619**			**565**	**18.0%**	**0.79 [0.19, 1.38]**
Heterogeneity: Tau² = 0.53; Chi² = 115.07, df = 5 (P < 0.00001); I² = 96%								
Test for overall effect: Z = 2.57 (P = 0.01)								
**Total (95% CI)**			**3402**			**2865**	**100.0%**	**0.55 [0.34, 0.76]**
Heterogeneity: Tau² = 0.34; Chi² = 497.39, df = 33 (P < 0.00001); I² = 93%								
Test for overall effect: Z = 5.23 (P < 0.00001)								
Test for subgroup differences: Chi² = 0.79, df = 1 (P = 0.37), I² = 0%								

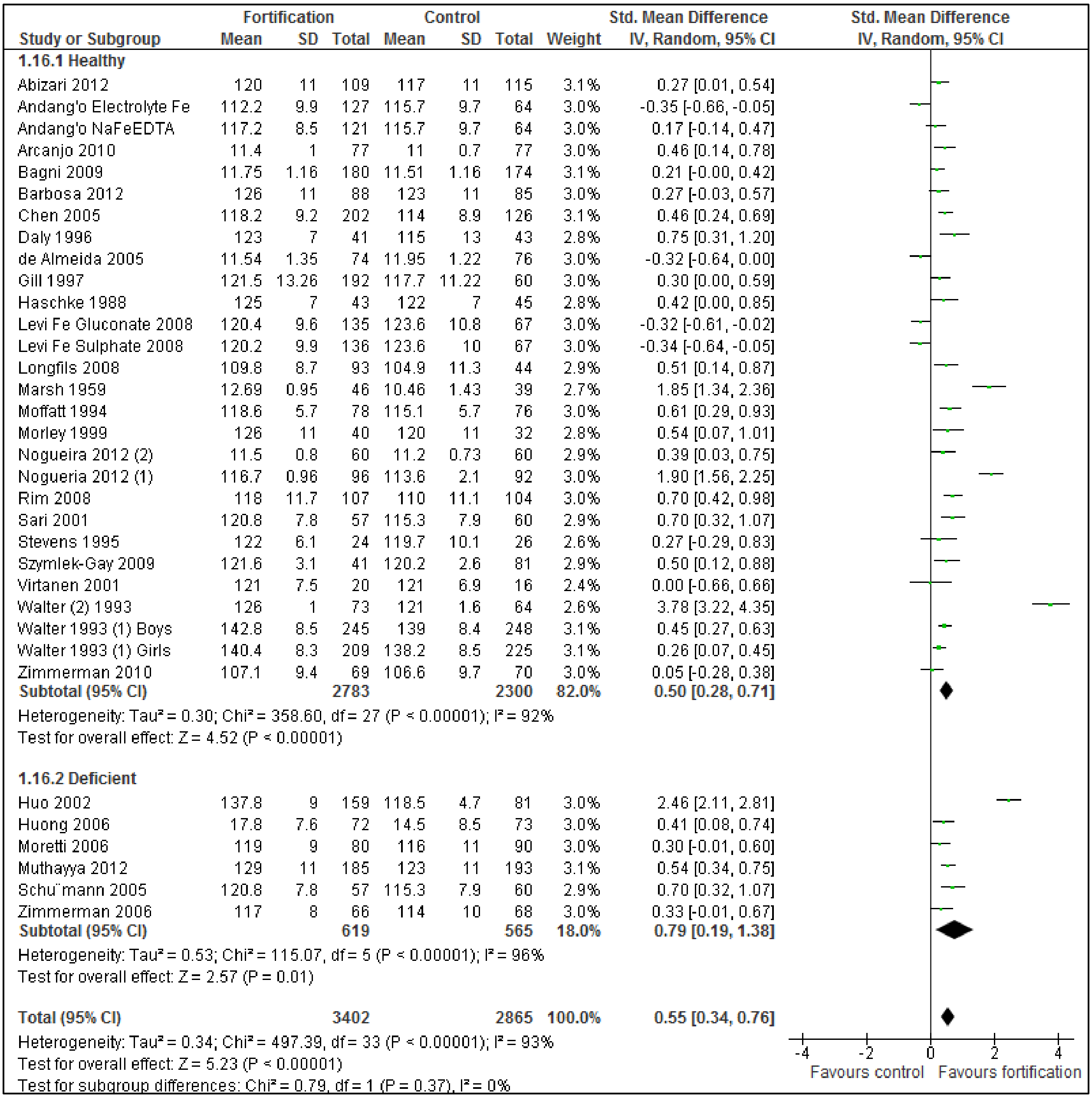

Figure S2 Effect of Iron fortifications on anemia prevalence in children

Study or Subgroup	log[Risk Ratio]	SE	Fortification Total	Control Total	Weight	Risk Ratio IV, Random, 95% CI
**1.17.1 Healthy**						
Abizari 2012	-0.0244	0.1848	109	115	7.3%	0.98 [0.68, 1.40]
Andang'o Electrolyte Fe	-0.1774	0.1763	121	64	7.4%	0.84 [0.59, 1.18]
Andang'o NaFeEDTA	0.311	0.1433	127	64	7.7%	1.36 [1.03, 1.81]
Arcanjo 2010	-0.5232	0.2712	77	77	6.3%	0.59 [0.35, 1.01]
Bagni 2009	-0.3567	0.1725	180	174	7.4%	0.70 [0.50, 0.98]
Chen 2005	-0.6811	0.171	202	126	7.4%	0.51 [0.36, 0.71]
Daly 1996	-2.5914	1.0118	41	43	1.5%	0.07 [0.01, 0.54]
Gill 1997	-0.1981	0.3883	192	60	5.0%	0.82 [0.38, 1.76]
Levi Fe Gluconate 2008	-0.8518	0.5794	135	144	3.4%	0.43 [0.14, 1.33]
Levi Fe Sulphate 2008	-0.8591	0.5794	136	72	3.4%	0.42 [0.14, 1.32]
Nogueira 2012 (2)	-0.8109	0.295	60	60	6.1%	0.44 [0.25, 0.79]
Nogueria 2012 (1)	-0.6779	0.256	96	92	6.5%	0.51 [0.31, 0.84]
Rim 2008	-1.9506	0.6016	96	91	3.2%	0.14 [0.04, 0.46]
Sari 2001	-1.1119	0.4778	57	60	4.2%	0.33 [0.13, 0.84]
Stevens 1995	-2.1203	1.4646	24	26	0.8%	0.12 [0.01, 2.12]
Virtanen 2001	0.8873	1.6001	20	16	0.7%	2.43 [0.11, 55.89]
Zimmerman 2010	0.0144	0.0633	69	70	8.3%	1.01 [0.90, 1.15]
**Subtotal (95% CI)**			**1742**	**1354**	**86.5%**	**0.64 [0.50, 0.82]**
Heterogeneity: Tau² = 0.14; Chi² = 63.84, df = 16 (P < 0.00001); I² = 75%						
Test for overall effect: Z = 3.57 (P = 0.0004)						
**1.17.2 Deficient**						
Huo 2002	-3.6012	0.5767	159	81	3.4%	0.03 [0.01, 0.08]
Huong 2006	-0.4382	0.4541	72	73	4.4%	0.65 [0.26, 1.57]
Moretti 2006	-0.5754	0.3184	80	90	5.8%	0.56 [0.30, 1.05]
**Subtotal (95% CI)**			**311**	**244**	**13.5%**	**0.23 [0.04, 1.25]**
Heterogeneity: Tau² = 2.08; Chi² = 23.61, df = 2 (P < 0.00001); I² = 92%						
Test for overall effect: Z = 1.70 (P = 0.09)						
**Total (95% CI)**			**2053**	**1598**	**100.0%**	**0.55 [0.42, 0.72]**
Heterogeneity: Tau² = 0.22; Chi² = 101.58, df = 19 (P < 0.00001); I² = 81%						
Test for overall effect: Z = 4.33 (P < 0.0001)						
Test for subgroup differences: Chi² = 1.40, df = 1 (P = 0.24), I² = 28.8%						

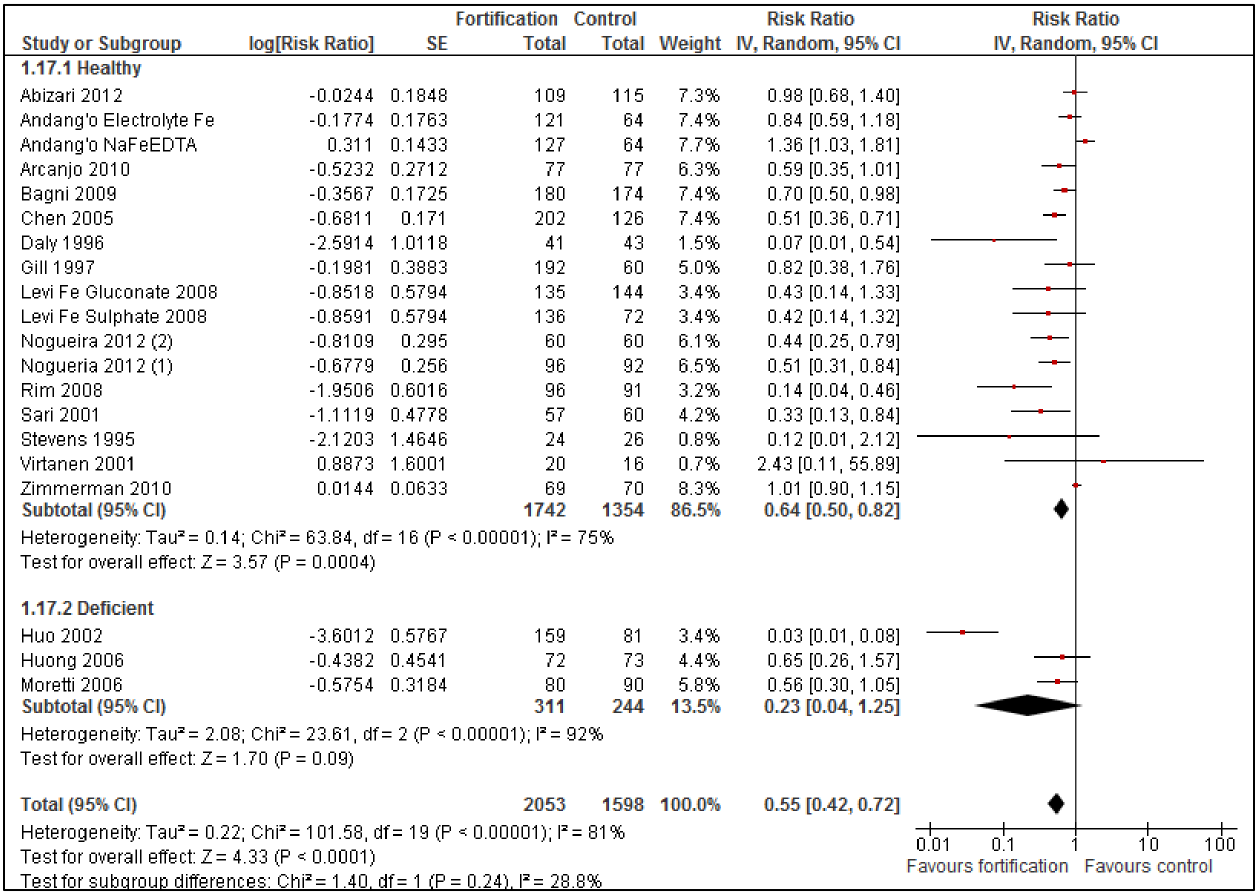

Figure S3 Effect of Iron fortification on serum ferritin levels in children

Study or Subgroup	Fortification Mean	Fortification SD	Fortification Total	Control Mean	Control SD	Control Total	Weight	Std. Mean Difference IV, Random, 95% CI
**1.18.1 Healthy**								
Daly 1996	30.5	17	41	15.9	12.2	43	12.0%	0.98 [0.53, 1.44]
Gill 1997	25.1	16.52	192	15.3	11.46	60	12.6%	0.63 [0.34, 0.93]
Levi Fe Gluconate 2008	20.1	2.5	135	20.1	2.1	67	12.6%	0.00 [-0.29, 0.29]
Levi Fe Sulphate 2008	22.3	2.3	136	20.1	2.1	67	12.6%	0.98 [0.67, 1.29]
Rim 2008	40.7	24	107	26.8	21.4	104	12.7%	0.61 [0.33, 0.88]
**Subtotal (95% CI)**			**611**			**341**	**62.6%**	**0.63 [0.28, 0.98]**
Heterogeneity: Tau² = 0.13; Chi² = 24.70, df = 4 (P < 0.0001); I² = 84%								
Test for overall effect: Z = 3.50 (P = 0.0005)								
**1.18.2 Deficient**								
Huo 2002	59.5	20	159	7.13	15.65	81	12.4%	2.80 [2.43, 3.17]
Huong 2006	18.5	30.9	72	-6.5	27.1	73	12.5%	0.86 [0.52, 1.20]
Moretti 2006	26.3	19	80	17.7	17	90	12.6%	0.48 [0.17, 0.78]
**Subtotal (95% CI)**			**311**			**244**	**37.4%**	**1.37 [0.01, 2.74]**
Heterogeneity: Tau² = 1.42; Chi² = 97.87, df = 2 (P < 0.00001); I² = 98%								
Test for overall effect: Z = 1.98 (P = 0.05)								
**Total (95% CI)**			**922**			**585**	**100.0%**	**0.91 [0.38, 1.44]**
Heterogeneity: Tau² = 0.55; Chi² = 151.49, df = 7 (P < 0.00001); I² = 95%								
Test for overall effect: Z = 3.38 (P = 0.0007)								
Test for subgroup differences: Chi² = 1.08, df = 1 (P = 0.30), I² = 7.8%								

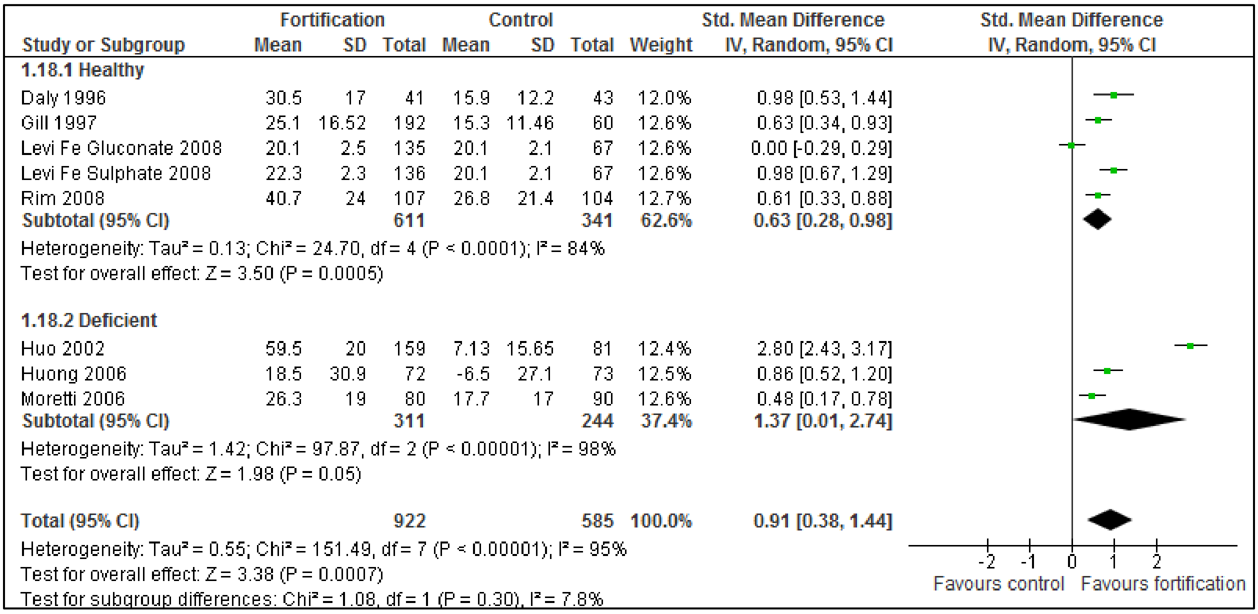

Figure S4 Effect of zinc fortification on serum zinc levels in children

	Fortification			Control				Std. Mean Difference
Study or Subgroup	Mean	SD	Total	Mean	SD	Total	Weight	IV, Random, 95% CI
**1.4.1 Full term healthy infants**								
Matsuda 1984	102	17	20	78	12	19	11.4%	1.59 [0.86, 2.32]
Salmenpera 1999	85	3	16	65	4	16	7.9%	5.51 [3.91, 7.11]
**Subtotal (95% CI)**			**36**			**35**	**19.3%**	**3.49 [-0.36, 7.33]**
Heterogeneity: Tau² = 7.29; Chi² = 19.11, df = 1 (P < 0.0001); I² = 95%								
Test for overall effect: Z = 1.78 (P = 0.08)								
**1.4.2 Newborns with VLBW**								
Diaz Gomez 2003	119	37	18	87	30	17	11.5%	0.93 [0.22, 1.63]
Friel 1993	95	17	15	93	30	14	11.4%	0.08 [-0.65, 0.81]
**Subtotal (95% CI)**			**33**			**31**	**22.9%**	**0.51 [-0.32, 1.34]**
Heterogeneity: Tau² = 0.22; Chi² = 2.68, df = 1 (P = 0.10); I² = 63%								
Test for overall effect: Z = 1.20 (P = 0.23)								
**1.4.3 Infants at risk of stunting**								
Brown 2007	78	12	70	77	11	77	12.5%	0.09 [-0.24, 0.41]
**Subtotal (95% CI)**			**70**			**77**	**12.5%**	**0.09 [-0.24, 0.41]**
Heterogeneity: Not applicable								
Test for overall effect: Z = 0.52 (P = 0.60)								
**1.4.4 Malnourished infants**								
Schlesinger 1992	122	28	18	118	38	17	11.6%	0.12 [-0.55, 0.78]
**Subtotal (95% CI)**			**18**			**17**	**11.6%**	**0.12 [-0.55, 0.78]**
Heterogeneity: Not applicable								
Test for overall effect: Z = 0.35 (P = 0.73)								
**1.4.5 School going children**								
Hambidge 1979	77	14	31	71	14	28	12.1%	0.42 [-0.09, 0.94]
Ohiokpehai 2009	66.6	7.18	134	54.92	5.887	154	12.6%	1.79 [1.51, 2.06]
**Subtotal (95% CI)**			**165**			**182**	**24.7%**	**1.12 [-0.21, 2.46]**
Heterogeneity: Tau² = 0.89; Chi² = 20.86, df = 1 (P < 0.00001); I² = 95%								
Test for overall effect: Z = 1.65 (P = 0.10)								
**1.4.6 School going children with asymptomatic zinc deficiency**								
Kilic 1998	82	9	9	63	3	10	9.0%	2.77 [1.43, 4.11]
**Subtotal (95% CI)**			**9**			**10**	**9.0%**	**2.77 [1.43, 4.11]**
Heterogeneity: Not applicable								
Test for overall effect: Z = 4.06 (P < 0.0001)								
**Total (95% CI)**			**331**			**352**	**100.0%**	**1.28 [0.56, 2.01]**
Heterogeneity: Tau² = 1.07; Chi² = 119.70, df = 8 (P < 0.00001); I² = 93%								
Test for overall effect: Z = 3.46 (P = 0.0005)								
Test for subgroup differences: Chi² = 19.40, df = 5 (P = 0.002), I² = 74.2%								

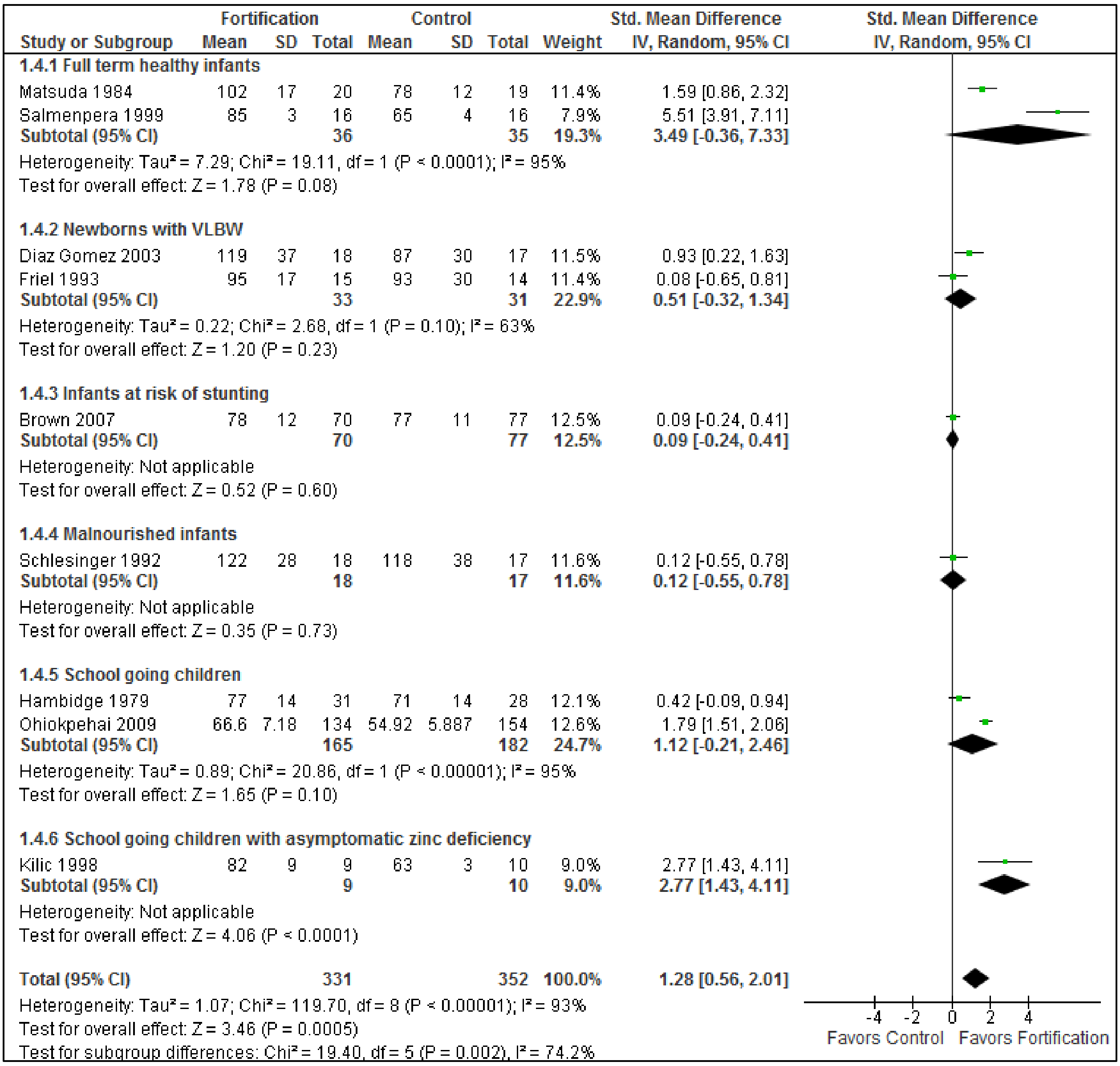

Figure S5 Effect of zinc fortification on height gain in children

Study or Subgroup	Experimental Mean	Experimental SD	Experimental Total	Control Mean	Control SD	Control Total	Weight	Std. Mean Difference IV, Random, 95% CI
**4.2.1 Healthy newborns**								
Matsuda 1984	1.01	0.42	20	1.01	0.41	18	13.5%	0.00 [-0.64, 0.64]
Salmenpera 1999 (Boys)	0.71	0.04	10	0.84	0.03	7	7.5%	-3.40 [-5.02, -1.77]
Salmenpera 1999 (Girls)	0.65	0.05	6	0.7	0.03	9	10.1%	-1.21 [-2.36, -0.06]
Walravens1976	1.01	0.03	24	0.94	0.03	18	12.4%	2.29 [1.49, 3.09]
**Subtotal (95% CI)**			**60**			**52**	**43.4%**	**-0.48 [-2.45, 1.48]**
Heterogeneity: Tau² = 3.71; Chi² = 51.24, df = 3 (P < 0.00001); I² = 94%								
Test for overall effect: Z = 0.48 (P = 0.63)								
**4.2.2 Newborns with VLBW**								
Diaz Gomez 2003	1.04	0.07	18	0.99	0.07	18	13.2%	0.70 [0.02, 1.37]
**Subtotal (95% CI)**			**18**			**18**	**13.2%**	**0.70 [0.02, 1.37]**
Heterogeneity: Not applicable								
Test for overall effect: Z = 2.03 (P = 0.04)								
**4.2.3 Infants at risk of stunting**								
Brown 2007	0.382	0.061	84	0.379	0.06	92	15.2%	0.05 [-0.25, 0.35]
**Subtotal (95% CI)**			**84**			**92**	**15.2%**	**0.05 [-0.25, 0.35]**
Heterogeneity: Not applicable								
Test for overall effect: Z = 0.33 (P = 0.74)								
**4.2.4 Malnourished infants**								
Schlesinger 1992	0.62	0.23	19	0.58	0.26	20	13.5%	0.16 [-0.47, 0.79]
**Subtotal (95% CI)**			**19**			**20**	**13.5%**	**0.16 [-0.47, 0.79]**
Heterogeneity: Not applicable								
Test for overall effect: Z = 0.50 (P = 0.62)								
**4.2.5 School going children**								
Hambidge 1979	0.18	0.031	46	0.17	0.032	42	14.7%	0.31 [-0.11, 0.74]
**Subtotal (95% CI)**			**46**			**42**	**14.7%**	**0.31 [-0.11, 0.74]**
Heterogeneity: Not applicable								
Test for overall effect: Z = 1.47 (P = 0.14)								
**Total (95% CI)**			**227**			**224**	**100.0%**	**0.08 [-0.53, 0.69]**
Heterogeneity: Tau² = 0.62; Chi² = 54.71, df = 7 (P < 0.00001); I² = 87%								
Test for overall effect: Z = 0.24 (P = 0.81)								
Test for subgroup differences: Chi² = 3.84, df = 4 (P = 0.43), I² = 0%								

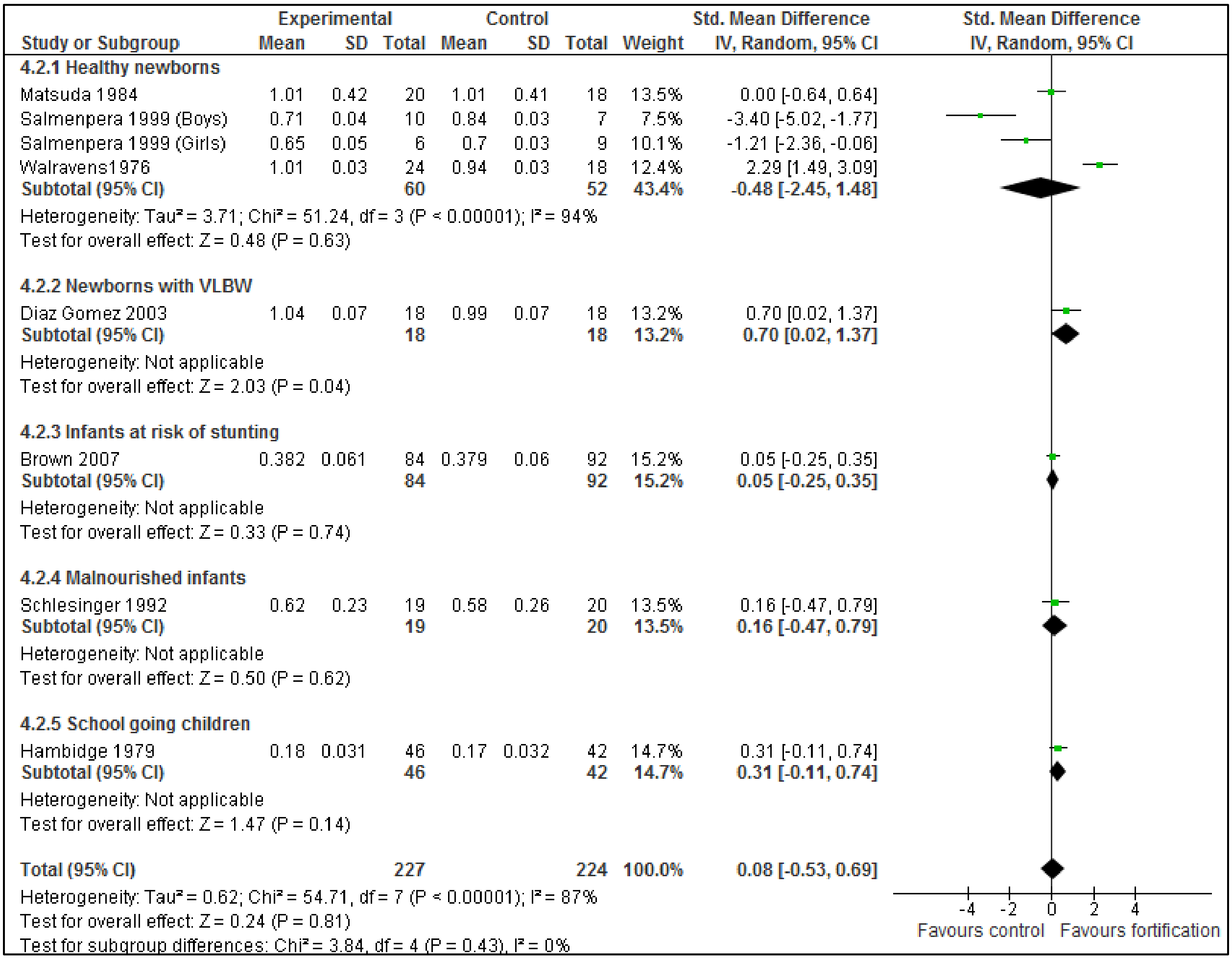

Figure S6 Effect of zinc fortification on weight gain in children

Study or Subgroup	Fortification Mean	Fortification SD	Fortification Total	Control Mean	Control SD	Control Total	Weight	Std. Mean Difference IV, Random, 95% CI
**3.2.1 Healthy newborns**								
Matsuda 1984	26.4	8.3	20	26.5	7	18	16.4%	-0.01 [-0.65, 0.62]
Walravens1976	23.8	0.58	24	21.48	0.94	18	13.8%	3.02 [2.10, 3.93]
**Subtotal (95% CI)**			**44**			**36**	**30.2%**	**1.48 [-1.49, 4.45]**
Heterogeneity: Tau² = 4.43; Chi² = 28.48, df = 1 (P < 0.00001); I² = 96%								
Test for overall effect: Z = 0.98 (P = 0.33)								
**3.2.2 Newborns with VLBW**								
Diaz Gomez 2003	25.7	3.43	18	24.86	0.9	18	16.2%	0.33 [-0.33, 0.99]
**Subtotal (95% CI)**			**18**			**18**	**16.2%**	**0.33 [-0.33, 0.99]**
Heterogeneity: Not applicable								
Test for overall effect: Z = 0.98 (P = 0.33)								
**3.2.3 Infants at risk of stunting**								
Brown 2007	7.22	2.78	84	7.22	2.78	92	19.0%	0.00 [-0.30, 0.30]
**Subtotal (95% CI)**			**84**			**92**	**19.0%**	**0.00 [-0.30, 0.30]**
Heterogeneity: Not applicable								
Test for overall effect: Z = 0.00 (P = 1.00)								
**3.2.4 Malnourished infants**								
Schlesinger 1992	24.9	6.3	19	25.8	10.2	20	16.4%	-0.10 [-0.73, 0.52]
**Subtotal (95% CI)**			**19**			**20**	**16.4%**	**-0.10 [-0.73, 0.52]**
Heterogeneity: Not applicable								
Test for overall effect: Z = 0.32 (P = 0.75)								
**3.2.5 School going children**								
Hambidge 1979	7.97	2.3	46	7.45	1.67	42	18.2%	0.25 [-0.17, 0.67]
**Subtotal (95% CI)**			**46**			**42**	**18.2%**	**0.25 [-0.17, 0.67]**
Heterogeneity: Not applicable								
Test for overall effect: Z = 1.19 (P = 0.23)								
**Total (95% CI)**			**211**			**208**	**100.0%**	**0.50 [-0.12, 1.11]**
Heterogeneity: Tau² = 0.49; Chi² = 39.88, df = 5 (P < 0.00001); I² = 87%								
Test for overall effect: Z = 1.59 (P = 0.11)								
Test for subgroup differences: Chi² = 2.65, df = 4 (P = 0.62), I² = 0%								

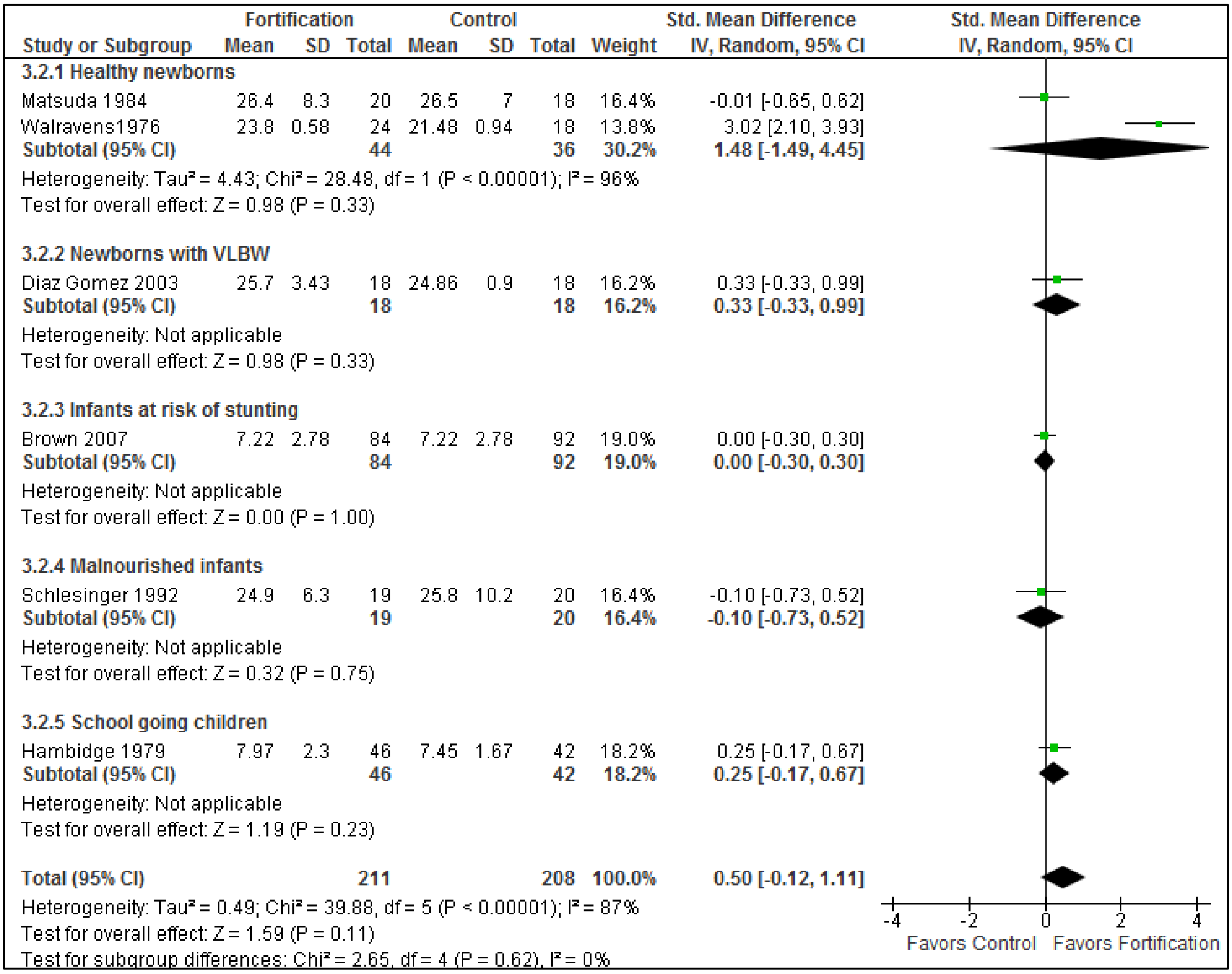

Figure S7 Effect of zinc fortification on serum hemoglobin levels in children

Study or Subgroup	Fortification Mean	Fortification SD	Fortification Total	Control Mean	Control SD	Control Total	Weight	Std. Mean Difference IV, Fixed, 95% CI
**2.2.1 Zinc Sulphate**								
Diaz Gomez 2003	12.2	1.1	16	12.2	0.9	18	37.3%	0.00 [-0.67, 0.67]
**Subtotal (95% CI)**			**16**			**18**	**37.3%**	**0.00 [-0.67, 0.67]**
Heterogeneity: Not applicable								
Test for overall effect: Z = 0.00 (P = 1.00)								
**2.2.2 Zinc Chloride**								
Schlesinger 1992	11.4	1	19	11.8	1	20	42.0%	-0.39 [-1.03, 0.24]
**Subtotal (95% CI)**			**19**			**20**	**42.0%**	**-0.39 [-1.03, 0.24]**
Heterogeneity: Not applicable								
Test for overall effect: Z = 1.21 (P = 0.23)								
**2.2.3 Zinc acetate**								
Kilic 1998	12.77	1.1	9	12.39	1.42	10	20.6%	0.28 [-0.62, 1.19]
**Subtotal (95% CI)**			**9**			**10**	**20.6%**	**0.28 [-0.62, 1.19]**
Heterogeneity: Not applicable								
Test for overall effect: Z = 0.61 (P = 0.54)								
**Total (95% CI)**			**44**			**48**	**100.0%**	**-0.11 [-0.52, 0.31]**

Heterogeneity: Chi² = 1.59, df = 2 (P = 0.45); I² = 0%
Test for overall effect: Z = 0.51 (P = 0.61)
Test for subgroup differences: Chi² = 1.59, df = 2 (P = 0.45), I² = 0%

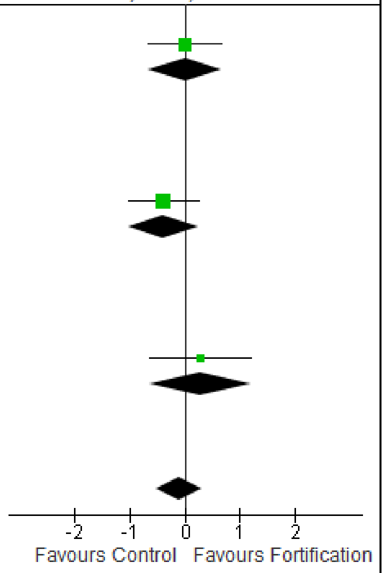

Figure S8 Effect of zinc fortification on serum copper levels in children

Study or Subgroup	Fortification Mean	Fortification SD	Fortification Total	Control Mean	Control SD	Control Total	Weight	Std. Mean Difference IV, Random, 95% CI
Kilic 1998	80.832	27.07	9	98.75	28.74	10	23.7%	-0.61 [-1.54, 0.31]
Matsuda 1984	111	31	20	124	21	19	25.4%	-0.48 [-1.12, 0.16]
Schlesinger 1992	155	28	18	149	29	17	25.3%	0.21 [-0.46, 0.87]
Walravens1976	124.2	5.8	34	112.3	6.1	34	25.7%	1.98 [1.39, 2.56]
**Total (95% CI)**			**81**			**80**	**100.0%**	**0.29 [-0.96, 1.54]**

Heterogeneity: Tau² = 1.49; Chi² = 39.74, df = 3 (P < 0.00001); I² = 92%
Test for overall effect: Z = 0.46 (P = 0.65)

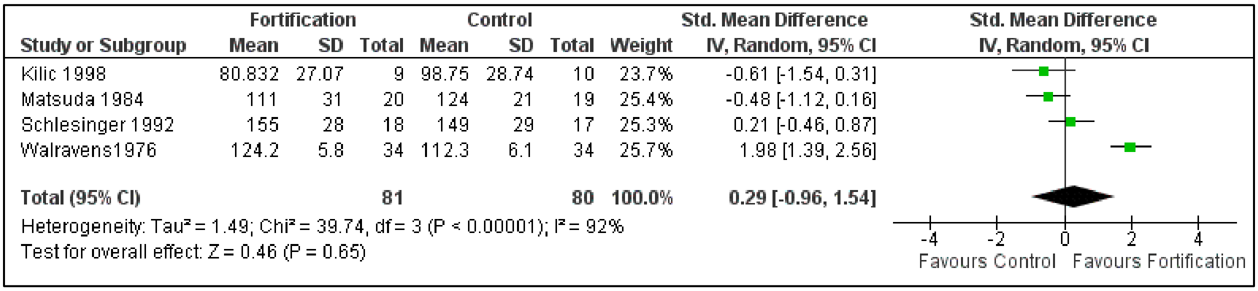

Figure S9 Effect of vitamin D and calcium fortification on serum vitamin D levels in children

Study or Subgroup	Fortification Mean	Fortification SD	Fortification Total	Control Mean	Control SD	Control Total	Weight	Std. Mean Difference IV, Random, 95% CI
**4.7.1 Calcium Only**								
Du 2004 (Calcium)	17.9	9	111	19.4	10.2	122	20.1%	-0.15 [-0.41, 0.10]
**Subtotal (95% CI)**			**111**			**122**	**20.1%**	**-0.15 [-0.41, 0.10]**
Heterogeneity: Not applicable								
Test for overall effect: Z = 1.18 (P = 0.24)								
**4.7.2 Vitamin D Only**								
Graham 2008	50	16	89	44	14	83	20.0%	0.40 [0.09, 0.70]
Rich-Edwards 2011 (UHT)	72.5	25	137	20	10	101	19.9%	2.61 [2.26, 2.96]
Rich-Edwards 2011 (Mangolia	50	15	140	20	10	101	20.0%	2.28 [1.95, 2.60]
**Subtotal (95% CI)**			**366**			**285**	**59.9%**	**1.76 [0.37, 3.15]**
Heterogeneity: Tau² = 1.48; Chi² = 109.10, df = 2 (P < 0.00001); I² = 98%								
Test for overall effect: Z = 2.48 (P = 0.01)								
**4.7.3 Calcium and Vitamin D Only**								
Du 2004 (Calcium & Vit D)	47.6	23.4	113	19.4	10.2	122	20.0%	1.58 [1.28, 1.87]
**Subtotal (95% CI)**			**113**			**122**	**20.0%**	**1.58 [1.28, 1.87]**
Heterogeneity: Not applicable								
Test for overall effect: Z = 10.54 (P < 0.00001)								
**Total (95% CI)**			**590**			**529**	**100.0%**	**1.34 [0.28, 2.39]**
Heterogeneity: Tau² = 1.43; Chi² = 243.07, df = 4 (P < 0.00001); I² = 98%								
Test for overall effect: Z = 2.48 (P = 0.01)								
Test for subgroup differences: Chi² = 78.30, df = 2 (P < 0.00001), I² = 97.4%								

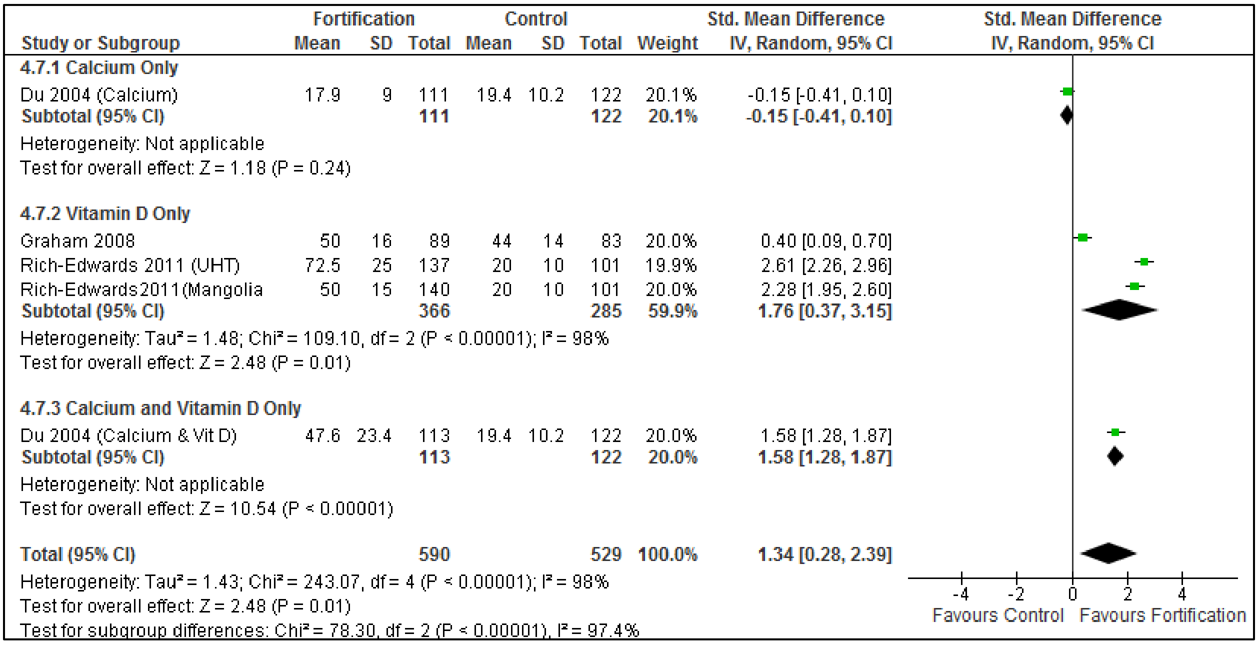

Figure S10 Effect of vitamin D and calcium fortification on serum PTH levels in children

Study or Subgroup	Favours Intervention Mean	Favours Intervention SD	Favours Intervention Total	Favours Control Mean	Favours Control SD	Favours Control Total	Weight	Std. Mean Difference IV, Random, 95% CI
**4.6.1 Calcium Only**								
Du 2004 (Calcium)	6.68	3.03	111	8.19	6.3	122	36.9%	-0.30 [-0.56, -0.04]
Zhu 2005 (Calcium)	6.67	3.03	43	7.6	4.83	41	13.4%	-0.23 [-0.66, 0.20]
**Subtotal (95% CI)**			**154**			**163**	**50.3%**	**-0.28 [-0.50, -0.06]**
Heterogeneity: Tau² = 0.00; Chi² = 0.08, df = 1 (P = 0.78); I² = 0%								
Test for overall effect: Z = 2.49 (P = 0.01)								
**4.6.3 Calcium and Vitamin D only**								
Du 2004 (Calcium & Vit D)	5.64	3.34	113	8.19	6.3	122	36.6%	-0.50 [-0.76, -0.24]
Zhu 2005(Calcium & Vit D)	5.36	2.78	44	7.6	4.83	41	13.1%	-0.57 [-1.00, -0.13]
**Subtotal (95% CI)**			**157**			**163**	**49.7%**	**-0.52 [-0.74, -0.29]**
Heterogeneity: Tau² = 0.00; Chi² = 0.07, df = 1 (P = 0.79); I² = 0%								
Test for overall effect: Z = 4.54 (P < 0.00001)								
**Total (95% CI)**			**311**			**326**	**100.0%**	**-0.40 [-0.56, -0.24]**
Heterogeneity: Tau² = 0.00; Chi² = 2.31, df = 3 (P = 0.51); I² = 0%								
Test for overall effect: Z = 4.97 (P < 0.00001)								
Test for subgroup differences: Chi² = 2.16, df = 1 (P = 0.14), I² = 53.7%								

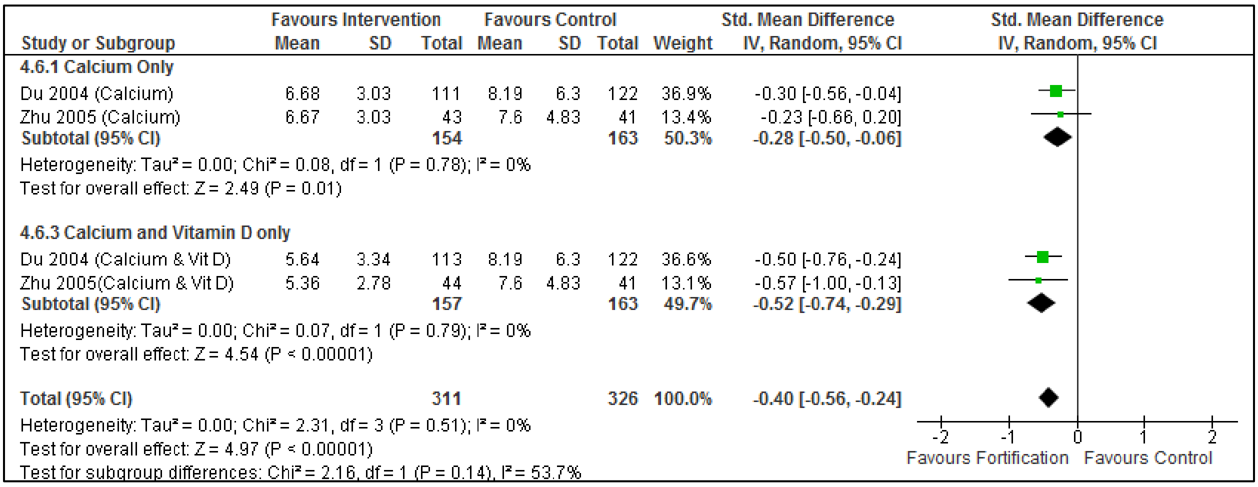

Figure S11 Effect of vitamin D and calcium fortification on serum calcium levels in children

Study or Subgroup	Experimental Mean	Experimental SD	Experimental Total	Control Mean	Control SD	Control Total	Weight	Std. Mean Difference IV, Random, 95% CI
**4.8.1 Calcium only**								
Du 2004 (Calcium)	6.68	3.03	111	8.19	6.3	122	50.2%	-0.30 [-0.56, -0.04]
**Subtotal (95% CI)**			**111**			**122**	**50.2%**	**-0.30 [-0.56, -0.04]**
Heterogeneity: Not applicable								
Test for overall effect: Z = 2.27 (P = 0.02)								
**4.8.3 Calcium and Vitamin D Only**								
Du 2004 (Calcium & Vit D)	5.64	3.34	113	8.19	6.3	122	49.8%	-0.50 [-0.76, -0.24]
**Subtotal (95% CI)**			**113**			**122**	**49.8%**	**-0.50 [-0.76, -0.24]**
Heterogeneity: Not applicable								
Test for overall effect: Z = 3.76 (P = 0.0002)								
**Total (95% CI)**			**224**			**244**	**100.0%**	**-0.40 [-0.59, -0.20]**
Heterogeneity: Tau² = 0.00; Chi² = 1.13, df = 1 (P = 0.29); I² = 11%								
Test for overall effect: Z = 4.02 (P < 0.0001)								
Test for subgroup differences: Chi² = 1.13, df = 1 (P = 0.29), I² = 11.4%								

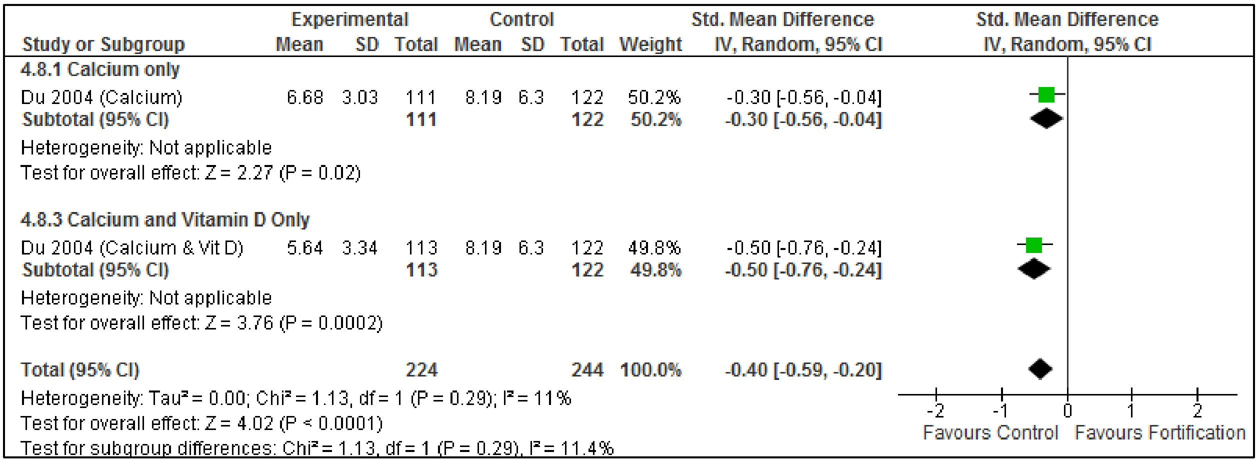

Figure S12 Effect of Vitamin A fortification on serum retinol concentration in children

Study or Subgroup	Fortification Mean	Fortification SD	Fortification Total	Control Mean	Control SD	Control Total	Weight	Std. Mean Difference IV, Random, 95% CI
Solon 1979	28.5	14.8	387	16.4	11.9	391	22.1%	0.90 [0.75, 1.05]
Solon 2000	1.32	0.37	396	1.17	0.33	396	22.3%	0.43 [0.29, 0.57]
Zhang 2010 (a)	1.15	0.27	87	1.02	0.23	87	16.7%	0.52 [0.21, 0.82]
Zhang 2010 (b)	1.27	0.32	144	1.05	0.28	144	19.0%	0.73 [0.49, 0.97]
Zhang 2010 (c)	1.18	0.28	165	1.06	0.26	165	19.8%	0.44 [0.22, 0.66]
**Total (95% CI)**			**1179**			**1183**	**100.0%**	**0.61 [0.39, 0.83]**
Heterogeneity: Tau² = 0.05; Chi² = 24.87, df = 4 (P < 0.0001); I² = 84%								
Test for overall effect: Z = 5.46 (P < 0.00001)								

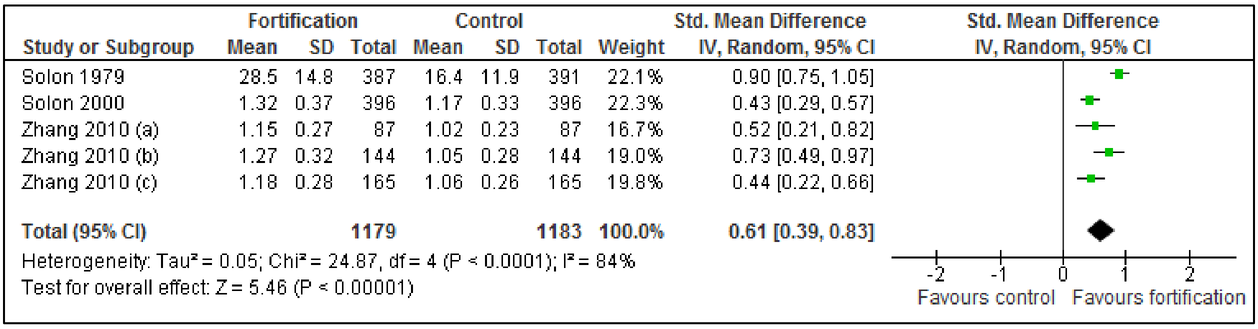

Figure S13 Effect of Vitamin A fortification on Vitamin A deficiency in children

Study or Subgroup	log[Risk Ratio]	SE	Fortification Total	Control Total	Weight	Risk Ratio IV, Random, 95% CI
Zhang 2010 (a)	-1.5002	0.7603	141	173	22.9%	0.22 [0.05, 0.99]
Zhang 2010 (b)	-1.9608	0.738	165	209	23.2%	0.14 [0.03, 0.60]
Zhang 2010 (c)	-1.3406	0.6193	139	194	24.8%	0.26 [0.08, 0.88]
Solon 1979	0.6225	0.1912	202	242	29.0%	1.86 [1.28, 2.71]
**Total (95% CI)**			**647**	**818**	**100.0%**	**0.39 [0.09, 1.74]**
Heterogeneity: Tau² = 2.00; Chi² = 24.63, df = 3 (P < 0.0001); I² = 88%						
Test for overall effect: Z = 1.24 (P = 0.22)						

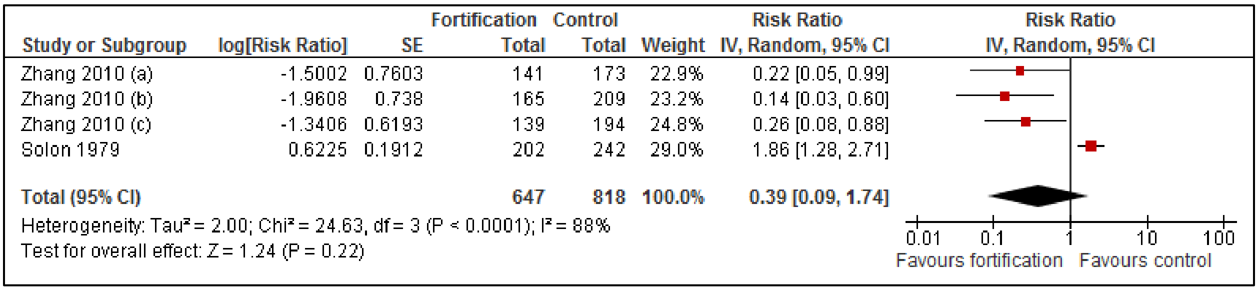

Figure 14 Effect of Vitamin A fortification on serum hemoglobin levels in children

Study or Subgroup	Fortification Mean	Fortification SD	Fortification Total	Control Mean	Control SD	Control Total	Weight	Std. Mean Difference IV, Random, 95% CI
Solon 2000	130	123	382	131	113	426	26.5%	-0.01 [-0.15, 0.13]
Zhang 2010 (a)	120.32	8.84	87	113.34	10.13	87	23.6%	0.73 [0.42, 1.04]
Zhang 2010 (b)	119.82	9.61	144	114.12	8.84	144	25.0%	0.62 [0.38, 0.85]
Zhang 2010 (c)	119.48	8.86	134	114.26	7.61	134	24.9%	0.63 [0.38, 0.88]
**Total (95% CI)**			**747**			**791**	**100.0%**	**0.48 [0.07, 0.89]**

Heterogeneity: Tau² = 0.16; Chi² = 40.53, df = 3 (P < 0.00001); I² = 93%
Test for overall effect: Z = 2.32 (P = 0.02)

Std. Mean Difference IV, Random, 95% CI

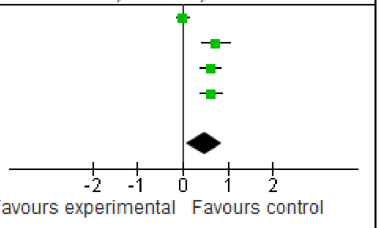

Figure S15 Effect of iodine fortification on urinary iodine concentration in children

Study or Subgroup	Fortification Mean	Fortification SD	Fortification Total	Control Mean	Control SD	Control Total	Weight	Std. Mean Difference IV, Random, 95% CI
Zimmermann 2003	104	11.38	526	28	5.34	419	50.6%	8.25 [7.86, 8.64]
Zimmermann 2006	74	6.75	47	19	18.75	24	49.4%	4.48 [3.57, 5.38]
**Total (95% CI)**			**573**			**443**	**100.0%**	**6.39 [2.69, 10.08]**

Heterogeneity: Tau² = 6.98; Chi² = 56.23, df = 1 (P < 0.00001); I² = 98%
Test for overall effect: Z = 3.39 (P = 0.0007)

Std. Mean Difference IV, Random, 95% CI

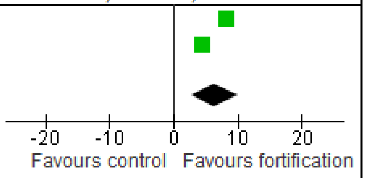

Figure S16 Effect of iodine fortification on serum thyroxin levels in children

Study or Subgroup	Fortification Mean	Fortification SD	Fortification Total	Control Mean	Control SD	Control Total	Weight	Std. Mean Difference IV, Random, 95% CI
Zimmermann 2003	126	29	641	137	36	419	51.3%	-0.34 [-0.47, -0.22]
Zimmermann 2006	118	24	47	87	23	24	48.7%	1.30 [0.76, 1.83]
**Total (95% CI)**			**688**			**443**	**100.0%**	**0.45 [-1.15, 2.06]**

Heterogeneity: Tau² = 1.30; Chi² = 33.82, df = 1 (P < 0.00001); I² = 97%
Test for overall effect: Z = 0.55 (P = 0.58)

Std. Mean Difference IV, Random, 95% CI

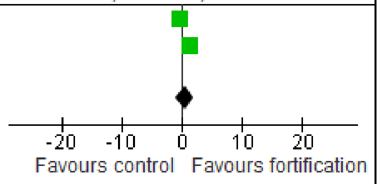

Figure S17 Effect of MMN fortification on serum hemoglobin levels in children

	Fortification			Control				Std. Mean Difference
**Study or Subgroup**	**Mean**	**SD**	**Total**	**Mean**	**SD**	**Total**	**Weight**	**IV, Random, 95% CI**
**3.3.1 LMIE**								
Kumar 2007	102	0.77	63	101	0.75	66	6.7%	1.31 [0.93, 1.69]
Lartey 1999	103	13	52	100	17	53	6.7%	0.20 [-0.19, 0.58]
Lein 2009	126.1	7.2	150	123.7	10.5	151	7.1%	0.27 [0.04, 0.49]
Nesamvuni 2005	11.4	2.2	16	10.8	2.4	20	5.7%	0.25 [-0.41, 0.91]
Nga 2009	121.2	7.3	114	120.1	8	118	7.0%	0.14 [-0.11, 0.40]
Phu 2010 (a)	114	7	106	109	8	61	6.8%	0.67 [0.35, 1.00]
Phu 2010 (b)	112.5	8	120	109	8	62	6.9%	0.44 [0.13, 0.75]
Sazawal 2010	109	11.4	233	95.4	15.4	232	7.1%	1.00 [0.81, 1.20]
Thankachan 2012	114	8.7	76	113	7.7	76	6.9%	0.12 [-0.20, 0.44]
**Subtotal (95% CI)**			**930**			**839**	**60.9%**	**0.50 [0.21, 0.78]**
Heterogeneity: Tau² = 0.16; Chi² = 63.93, df = 8 (P < 0.00001); I² = 87%								
Test for overall effect: Z = 3.41 (P = 0.0007)								
**3.3.2 UMIE**								
Faber 2005 (a)	119	11	144	110	14	142	7.0%	0.71 [0.47, 0.95]
Lopriore 2004	127	14	82	109	19	93	6.9%	1.06 [0.75, 1.38]
Lutter 2007	114.6	8.8	49	109.9	10.3	61	6.7%	0.48 [0.10, 0.86]
Stekel 1988	128	7	39	129	8	39	6.5%	-0.13 [-0.58, 0.31]
Villalpando 2006	127.4	0.5	58	124.1	0.59	57	4.9%	6.00 [5.13, 6.87]
Winichagoon 2006	121.3	9.54	261	120.6	9.63	257	7.2%	0.07 [-0.10, 0.25]
**Subtotal (95% CI)**			**633**			**649**	**39.1%**	**1.25 [0.45, 2.06]**
Heterogeneity: Tau² = 0.97; Chi² = 200.13, df = 5 (P < 0.00001); I² = 98%								
Test for overall effect: Z = 3.04 (P = 0.002)								
**Total (95% CI)**			**1563**			**1488**	**100.0%**	**0.75 [0.41, 1.08]**
Heterogeneity: Tau² = 0.40; Chi² = 264.48, df = 14 (P < 0.00001); I² = 95%								
Test for overall effect: Z = 4.36 (P < 0.0001)								
Test for subgroup differences: Chi² = 3.01, df = 1 (P = 0.08), I² = 66.7%								

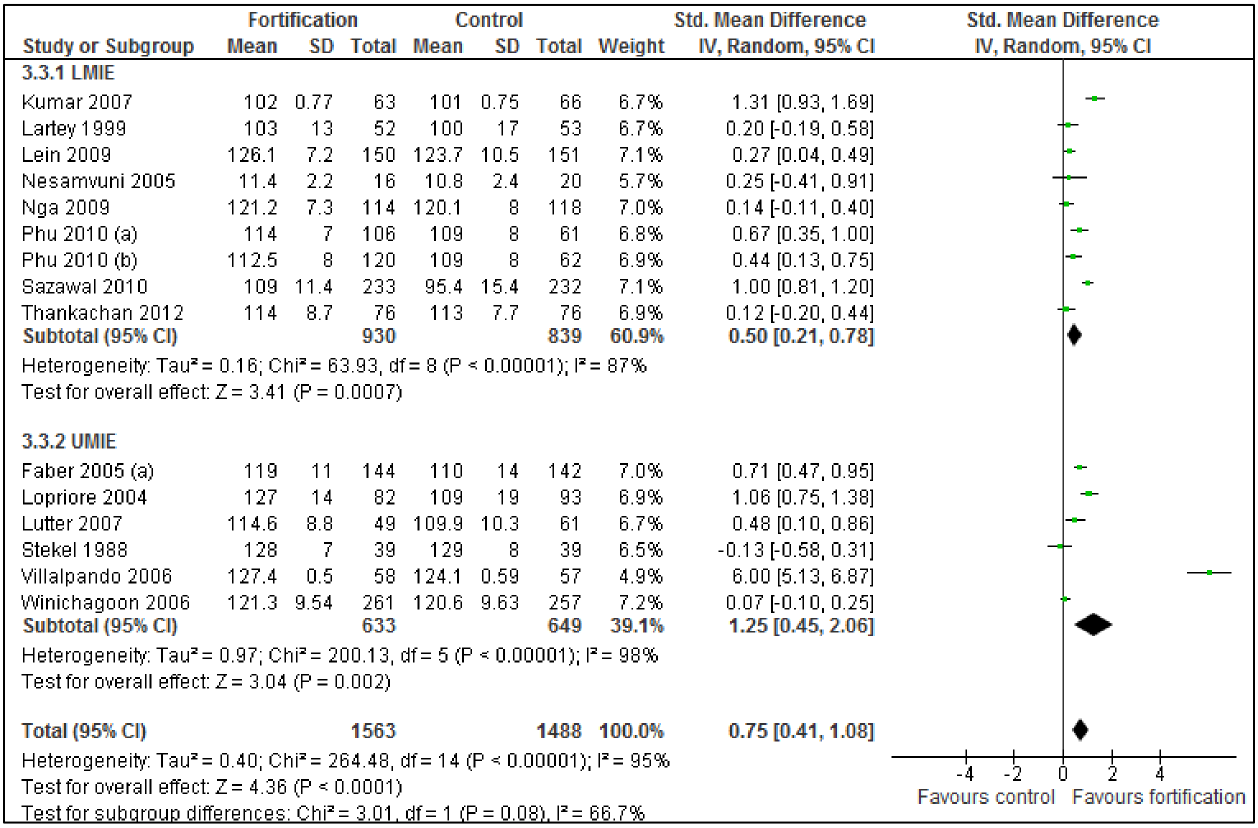

Figure S18 Effect of MMN fortification on serum ferritin levels in children

	Fortification			Control				Std. Mean Difference
**Study or Subgroup**	**Mean**	**SD**	**Total**	**Mean**	**SD**	**Total**	**Weight**	**IV, Random, 95% CI**
**3.8.1 LIE/LMIE**								
Lartey 1999	14.9	44.57	52	14.6	38.41	53	11.2%	0.01 [-0.38, 0.39]
Lein 2009	108.3	69.4	150	104.2	69.1	151	13.6%	0.06 [-0.17, 0.29]
Phu 2010 (a)	19.8	13.4	120	11.1	7.6	122	13.1%	0.80 [0.54, 1.06]
Phu 2010 (b)	20.8	13.85	105	11.1	7.61	122	12.9%	0.88 [0.61, 1.16]
Sazawal 2010	15.3	11.2	233	9.8	8.6	272	14.2%	0.56 [0.38, 0.73]
**Subtotal (95% CI)**			**660**			**720**	**65.1%**	**0.47 [0.14, 0.80]**
Heterogeneity: Tau² = 0.12; Chi² = 33.85, df = 4 (P < 0.00001); I² = 88%								
Test for overall effect: Z = 2.82 (P = 0.005)								
**3.8.2 UMIE/HIE**								
Faber 2005 (a)	15.8	57.5	141	6.5	35.3	138	13.5%	0.19 [-0.04, 0.43]
Lutter 2007	17.4	59.5	49	19.3	66	61	11.3%	-0.03 [-0.41, 0.35]
Stekel 1988	15.4	5.6	38	13	5.1	41	10.2%	0.44 [-0.00, 0.89]
**Subtotal (95% CI)**			**228**			**240**	**34.9%**	**0.18 [-0.03, 0.40]**
Heterogeneity: Tau² = 0.01; Chi² = 2.55, df = 2 (P = 0.28); I² = 22%								
Test for overall effect: Z = 1.67 (P = 0.10)								
**Total (95% CI)**			**888**			**960**	**100.0%**	**0.37 [0.13, 0.62]**
Heterogeneity: Tau² = 0.10; Chi² = 44.53, df = 7 (P < 0.00001); I² = 84%								
Test for overall effect: Z = 3.00 (P = 0.003)								
Test for subgroup differences: Chi² = 2.04, df = 1 (P = 0.15), I² = 51.1%								

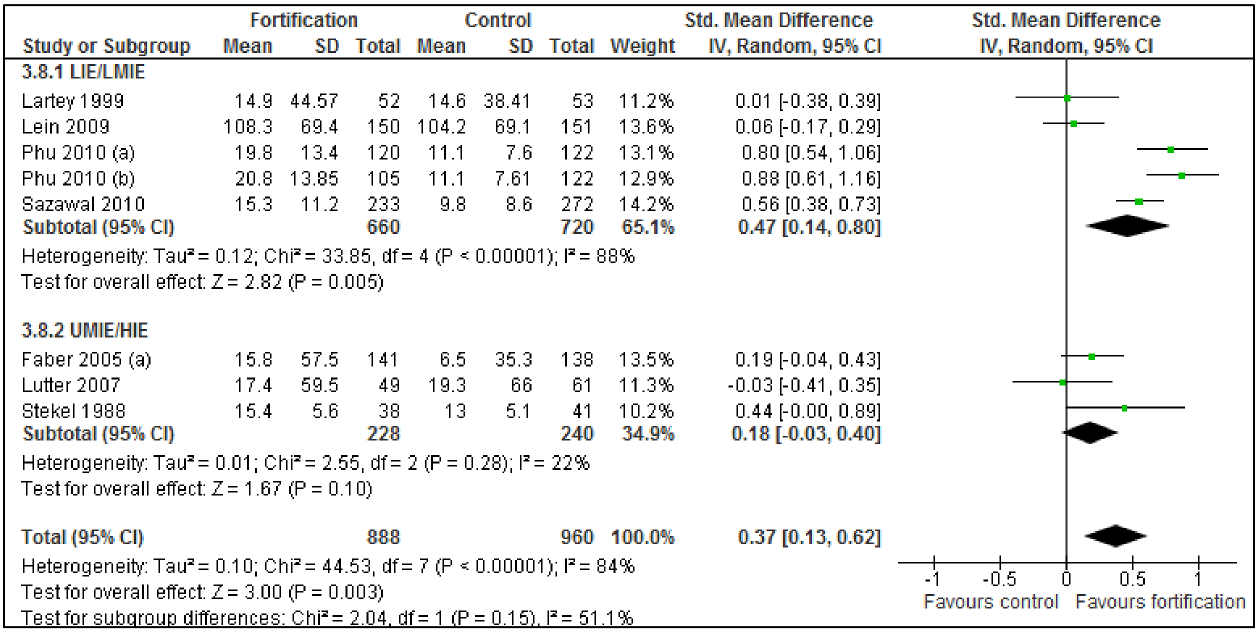

Figure S19 Effect of MMN fortification on serum zinc levels in children

Study or Subgroup	Fortification Mean	Fortification SD	Fortification Total	Control Mean	Control SD	Control Total	Weight	Std. Mean Difference IV, Random, 95% CI
**3.13.1 LMIE**								
Lartey 1999	15.9	2.8	52	15.5	4.3	53	5.2%	0.11 [-0.27, 0.49]
Lein 2009	12.6	2.6	150	12.2	2	151	9.8%	0.17 [-0.05, 0.40]
Lin 2007	10.7	2.6	125	10.4	2.4	115	8.7%	0.12 [-0.13, 0.37]
Nga 2009	9.4	1.9	114	8.8	1.9	118	8.5%	0.31 [0.06, 0.57]
Phu 2010 (a)	11.3	3.3	119	10.3	3.3	121	8.7%	0.30 [0.05, 0.56]
Phu 2010 (b)	10.7	4.1	105	10.3	3.3	121	8.4%	0.11 [-0.15, 0.37]
Sazawal 2010	61.4	26.8	233	63.4	29.4	232	11.8%	-0.07 [-0.25, 0.11]
Thankachan 2012	12.8	3.5	75	13.1	3	76	6.6%	-0.09 [-0.41, 0.23]
**Subtotal (95% CI)**			**973**			**987**	**67.7%**	**0.12 [0.01, 0.23]**
Heterogeneity: Tau² = 0.01; Chi² = 10.22, df = 7 (P = 0.18); I² = 32%								
Test for overall effect: Z = 2.09 (P = 0.04)								
**3.13.2 UMIE**								
Faber 2005 (a)	10.6	2.1	141	10.6	1.8	138	9.4%	0.00 [-0.23, 0.23]
Lutter 2007	13.6	4.1	49	13.2	3.4	61	5.3%	0.11 [-0.27, 0.48]
Villalpando 2006	12.61	0.96	58	13.07	1	57	5.4%	-0.47 [-0.84, -0.10]
Winichagoon 2006	11.2	1.63	247	10.9	1.67	261	12.1%	0.18 [0.01, 0.36]
**Subtotal (95% CI)**			**495**			**517**	**32.3%**	**-0.02 [-0.26, 0.23]**
Heterogeneity: Tau² = 0.04; Chi² = 9.90, df = 3 (P = 0.02); I² = 70%								
Test for overall effect: Z = 0.13 (P = 0.90)								
**Total (95% CI)**			**1468**			**1504**	**100.0%**	**0.08 [-0.02, 0.19]**
Heterogeneity: Tau² = 0.01; Chi² = 20.68, df = 11 (P = 0.04); I² = 47%								
Test for overall effect: Z = 1.59 (P = 0.11)								
Test for subgroup differences: Chi² = 0.93, df = 1 (P = 0.34), I² = 0%								

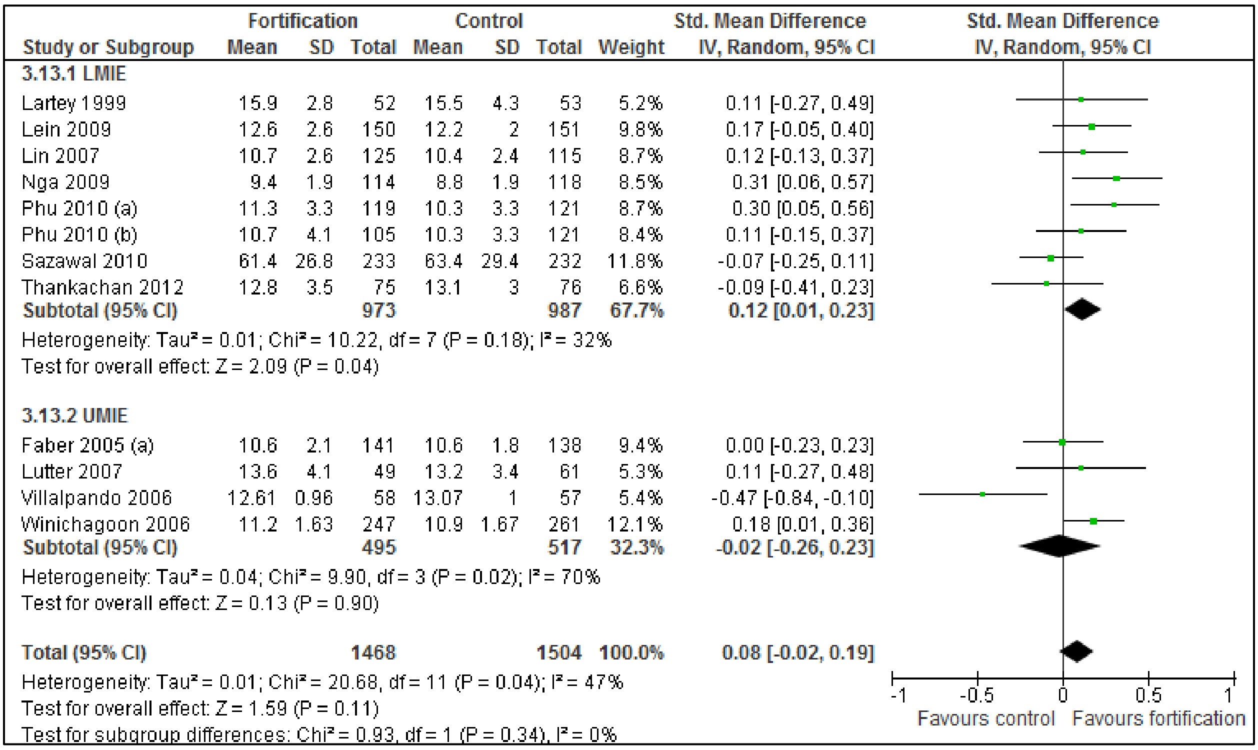

Figure S20 Effect of MMN fortification on serum retinol levels in children

Study or Subgroup	MMN Mean	MMN SD	MMN Total	Control Mean	Control SD	Control Total	Weight	Std. Mean Difference IV, Random, 95% CI
**3.33.1 LIE and LMIE**								
Faber 2005 (a)	1.21	0.35	68	1.1	0.3	69	9.8%	0.34 [-0.00, 0.67]
Faber 2005 (b)	0.8	0.28	71	0.95	0.26	69	9.8%	-0.55 [-0.89, -0.21]
Kumar 2007	41.4	14.8	63	46.2	18.6	66	9.6%	-0.28 [-0.63, 0.06]
Lartey 1999	0.84	0.4	52	0.93	0.4	53	8.9%	-0.22 [-0.61, 0.16]
Lein 2009	0.99	0.21	150	1.01	0.59	151	12.1%	-0.04 [-0.27, 0.18]
Nesamvuni 2005	1.3	0.35	16	1.35	0.49	20	5.0%	-0.11 [-0.77, 0.55]
Phu 2010 (a)	0.74	0.196	120	0.72	0.199	123	11.5%	0.10 [-0.15, 0.35]
Phu 2010 (b)	0.76	0.21	106	0.72	0.198	123	11.4%	0.20 [-0.06, 0.46]
**Subtotal (95% CI)**			**646**			**674**	**78.0%**	**-0.06 [-0.25, 0.14]**
Heterogeneity: Tau² = 0.05; Chi² = 20.71, df = 7 (P = 0.004); I² = 66%								
Test for overall effect: Z = 0.57 (P = 0.57)								
**3.33.2 UMIE and HIE**								
Lutter 2007	1.08	0.23	49	0.99	0.27	61	9.0%	0.35 [-0.03, 0.73]
Winichagoon 2006	1.3	0.31	241	1.4	0.36	256	13.0%	-0.30 [-0.47, -0.12]
**Subtotal (95% CI)**			**290**			**317**	**22.0%**	**0.01 [-0.63, 0.64]**
Heterogeneity: Tau² = 0.19; Chi² = 9.27, df = 1 (P = 0.002); I² = 89%								
Test for overall effect: Z = 0.02 (P = 0.99)								
**Total (95% CI)**			**936**			**991**	**100.0%**	**-0.05 [-0.23, 0.13]**
Heterogeneity: Tau² = 0.06; Chi² = 32.35, df = 9 (P = 0.0002); I² = 72%								
Test for overall effect: Z = 0.57 (P = 0.57)								
Test for subgroup differences: Chi² = 0.03, df = 1 (P = 0.85), I² = 0%								

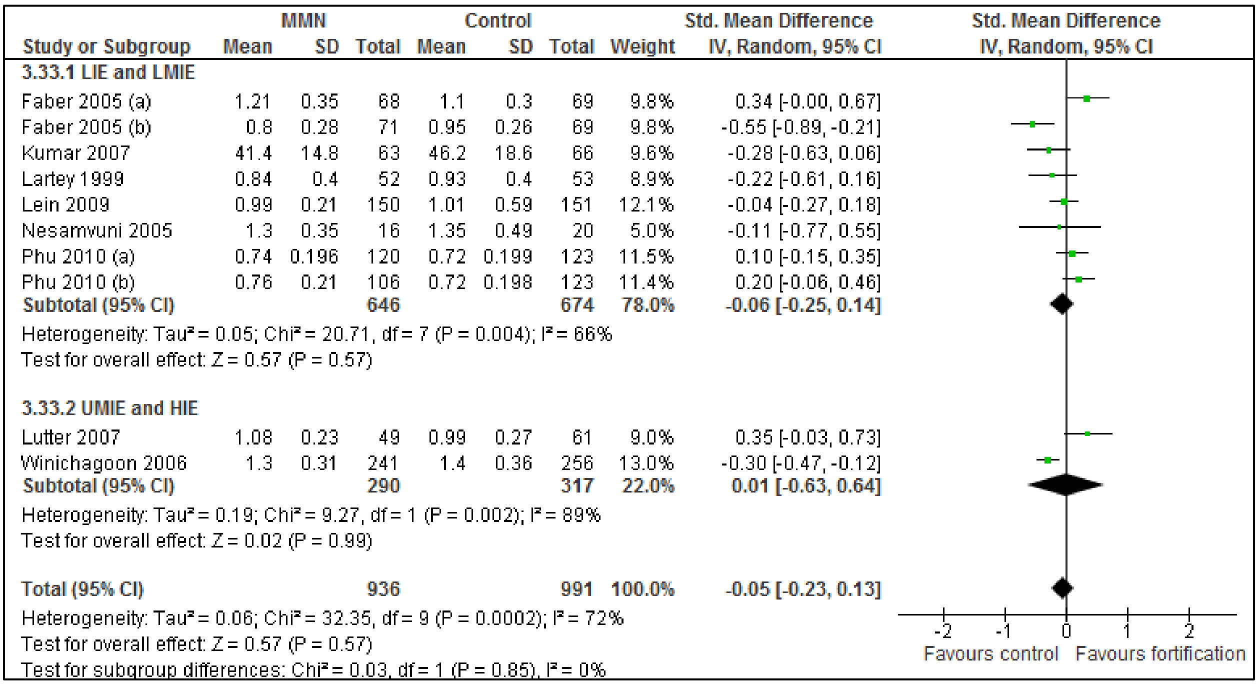

Figure S21 Effect of MMN fortification of vitamin A deficiency in children

Study or Subgroup	log[Risk Ratio]	SE	Fortification Total	Control Total	Weight	Risk Ratio IV, Fixed, 95% CI
**3.42.1 LIE and LMIE**						
Lartey 1999	0.2704	0.2981	52	53	8.2%	1.31 [0.73, 2.35]
Lein 2009	-0.4789	0.4343	150	151	3.9%	0.62 [0.26, 1.45]
Phu 2010 (a)	-0.1505	0.1506	120	123	32.2%	0.86 [0.64, 1.16]
Phu 2010 (b)	-0.2657	0.1653	106	123	26.7%	0.77 [0.55, 1.06]
Varma 2007	0.2601	0.3178	244	269	7.2%	1.30 [0.70, 2.42]
**Subtotal (95% CI)**			**672**	**719**	**78.2%**	**0.88 [0.73, 1.07]**
Heterogeneity: Chi² = 4.64, df = 4 (P = 0.33); I² = 14%						
Test for overall effect: Z = 1.28 (P = 0.20)						
**3.42.2 UMIE and HIE**						
Lutter 2007	-0.9849	0.6297	49	61	1.8%	0.37 [0.11, 1.28]
Winichagoon 2006	0.0332	0.191	266	269	20.0%	1.03 [0.71, 1.50]
**Subtotal (95% CI)**			**315**	**330**	**21.8%**	**0.95 [0.66, 1.36]**
Heterogeneity: Chi² = 2.39, df = 1 (P = 0.12); I² = 58%						
Test for overall effect: Z = 0.29 (P = 0.77)						
**Total (95% CI)**			**987**	**1049**	**100.0%**	**0.90 [0.76, 1.06]**
Heterogeneity: Chi² = 7.16, df = 6 (P = 0.31); I² = 16%						
Test for overall effect: Z = 1.27 (P = 0.20)						
Test for subgroup differences: Chi² = 0.12, df = 1 (P = 0.73), I² = 0%						

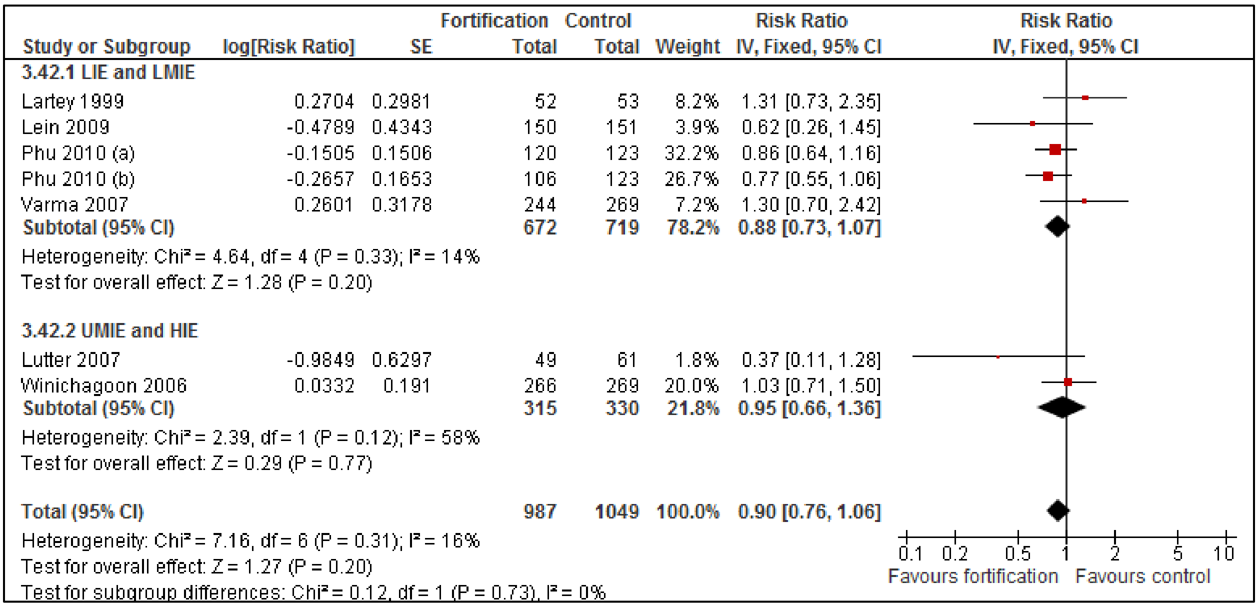

Figure S22 Effect of MMN fortification on anemia

Study or Subgroup	log[Risk Ratio]	SE	Fortification Total	Control Total	Weight	Risk Ratio IV, Random, 95% CI
**3.38.1 LIE and LMIE**						
Gibson 2011	-0.6006	0.1353	273	282	11.6%	0.55 [0.42, 0.72]
Lartey 1999	-0.0863	0.2597	52	53	8.8%	0.92 [0.55, 1.53]
Lein 2009	-0.7216	0.3043	150	151	7.8%	0.49 [0.27, 0.88]
Lopriore 2004	-1.6013	0.3525	82	93	6.9%	0.20 [0.10, 0.40]
Nga 2009	-0.2678	0.2917	114	118	8.1%	0.77 [0.43, 1.36]
Phu 2010 (a)	-0.4152	0.1441	120	123	11.5%	0.66 [0.50, 0.88]
Phu 2010 (b)	-0.6842	0.174	106	123	10.8%	0.50 [0.36, 0.71]
Varma 2007	-1.6252	0.3317	176	213	7.3%	0.20 [0.10, 0.38]
**Subtotal (95% CI)**			**1073**	**1156**	**72.9%**	**0.50 [0.37, 0.67]**
Heterogeneity: Tau² = 0.12; Chi² = 24.86, df = 7 (P = 0.0008); I² = 72%						
Test for overall effect: Z = 4.64 (P < 0.00001)						
**3.38.2 UMIE and HIE**						
Lutter 2007	-0.4377	0.2677	49	61	8.6%	0.65 [0.38, 1.09]
Rivera 2010	-0.8873	0.3372	357	210	7.2%	0.41 [0.21, 0.80]
Winichagoon 2006	0.0114	0.1512	58	91	11.3%	1.01 [0.75, 1.36]
**Subtotal (95% CI)**			**464**	**362**	**27.1%**	**0.69 [0.41, 1.15]**
Heterogeneity: Tau² = 0.15; Chi² = 6.90, df = 2 (P = 0.03); I² = 71%						
Test for overall effect: Z = 1.41 (P = 0.16)						
**Total (95% CI)**			**1537**	**1518**	**100.0%**	**0.55 [0.42, 0.71]**
Heterogeneity: Tau² = 0.13; Chi² = 39.80, df = 10 (P < 0.0001); I² = 75%						
Test for overall effect: Z = 4.54 (P < 0.00001)						
Test for subgroup differences: Chi² = 1.09, df = 1 (P = 0.30), I² = 8.7%						

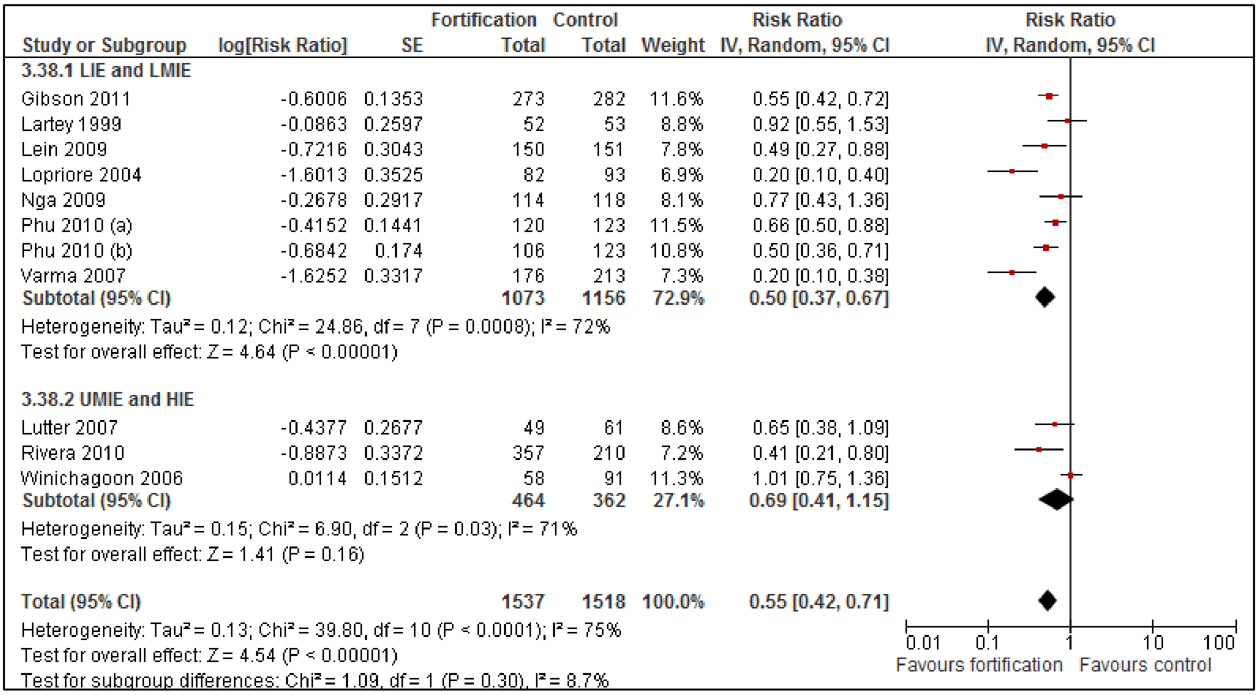

Figure S23 Effect of MMN fortification on Height-for-age Z Score in children

	MMN			Control				Std. Mean Difference
Study or Subgroup	Mean	SD	Total	Mean	SD	Total	Weight	IV, Random, 95% CI
Faber 2005 (a)	-0.94	1.05	143	-1.06	1.15	145	14.3%	0.11 [-0.12, 0.34]
Kuusipalo 2006 (a)	-2.3	0.6	9	-2.3	1	9	2.7%	0.00 [-0.92, 0.92]
Kuusipalo 2006 (b)	-2.3	0.7	9	-2.4	1	9	2.7%	0.11 [-0.81, 1.04]
Lein 2009	-1.64	0.78	150	-1.52	0.79	151	14.5%	-0.15 [-0.38, 0.07]
Lopriore 2004	-2.68	0.59	103	-2.84	0.71	106	12.9%	0.24 [-0.03, 0.52]
Lutter 2007	-1.5	0.99	49	-1.77	1.15	61	9.7%	0.25 [-0.13, 0.63]
Osei 2010	-2.2	1.15	249	-2.13	1.15	250	16.3%	-0.06 [-0.24, 0.11]
Oue'draogo 2010	-1.43	1	65	-1.57	0.7	60	10.4%	0.16 [-0.19, 0.51]
Sazawal 2010	0.28	0.47	267	0.09	0.42	257	16.4%	0.43 [0.25, 0.60]
**Total (95% CI)**			**1044**			**1048**	**100.0%**	**0.13 [-0.04, 0.29]**

Heterogeneity: Tau² = 0.03; Chi² = 22.87, df = 8 (P = 0.004); I² = 65%
Test for overall effect: Z = 1.54 (P = 0.12)

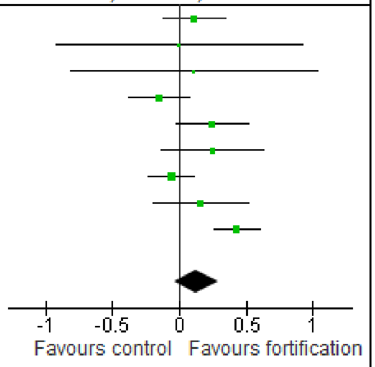

Figure S24 Effect of MMN fortification on Weight-for-age Z Score in children

	MMN			Control				Std. Mean Difference
Study or Subgroup	Mean	SD	Total	Mean	SD	Total	Weight	IV, Random, 95% CI
Faber 2005 (a)	0.05	1.25	143	0.09	1.37	145	17.1%	-0.03 [-0.26, 0.20]
Kuusipalo 2006 (a)	-2.3	0.5	9	-2.1	0.9	9	0.0%	-0.26 [-1.19, 0.67]
Kuusipalo 2006 (b)	-2.4	0.6	9	-2.2	1	9	0.0%	-0.23 [-1.16, 0.70]
Lein 2009	-1.68	0.64	150	-1.76	0.61	151	17.1%	0.13 [-0.10, 0.35]
Lopriore 2004	-1.71	0.63	103	-1.9	0.65	106	0.0%	0.30 [0.02, 0.57]
Lutter 2007	-2.62	0.91	49	-0.88	1.03	61	14.7%	-1.77 [-2.21, -1.32]
Osei 2010	-2.19	0.77	249	-2.1	0.77	250	17.5%	-0.12 [-0.29, 0.06]
Oue'draogo 2010	-1.34	0.99	65	-1.5	1.03	66	15.9%	0.16 [-0.19, 0.50]
Sazawal 2010	0.38	0.54	267	0.18	0.51	257	17.6%	0.38 [0.21, 0.55]
**Total (95% CI)**			**923**			**930**	**100.0%**	**-0.17 [-0.56, 0.22]**

Heterogeneity: Tau² = 0.22; Chi² = 82.57, df = 5 (P < 0.00001); I² = 94%
Test for overall effect: Z = 0.86 (P = 0.39)

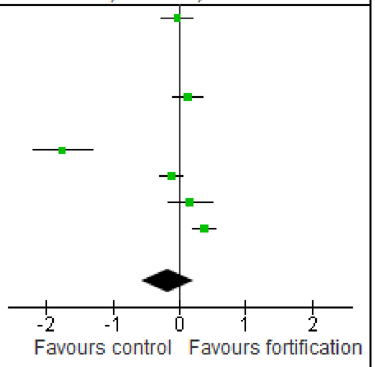

Figure S25 Effect of MMN fortification on Weight-for-Height Z Score in children

	MMN			Control				Std. Mean Difference
Study or Subgroup	Mean	SD	Total	Mean	SD	Total	Weight	IV, Random, 95% CI
Faber 2005 (a)	0.82	1.15	143	0.99	1.23	145	13.0%	-0.14 [-0.37, 0.09]
Kuusipalo 2006 (a)	-1	0.7	9	-0.9	1.2	9	5.7%	-0.10 [-1.02, 0.83]
Kuusipalo 2006 (b)	-1.3	0.6	9	-1.2	1.2	9	5.7%	-0.10 [-1.03, 0.82]
Lein 2009	-1.05	0.75	150	-1.14	0.68	151	13.0%	0.13 [-0.10, 0.35]
Lopriore 2004	-1.11	0.72	103	-0.28	0.66	106	12.3%	-1.20 [-1.49, -0.90]
Lutter 2007	0.19	0.87	49	0.04	0.97	61	11.4%	0.16 [-0.22, 0.54]
Osei 2010	-1.25	0.79	249	-1.16	0.72	250	13.5%	-0.12 [-0.29, 0.06]
Oue'draogo 2010	-0.79	0.99	65	-0.97	1.11	66	11.8%	0.17 [-0.17, 0.51]
Sazawal 2010	0.42	0.65	267	0.3	0.65	257	13.5%	0.18 [0.01, 0.36]
**Total (95% CI)**			**1044**			**1054**	**100.0%**	**-0.11 [-0.40, 0.17]**

Heterogeneity: Tau² = 0.15; Chi² = 71.52, df = 8 (P < 0.00001); I² = 89%
Test for overall effect: Z = 0.78 (P = 0.43)

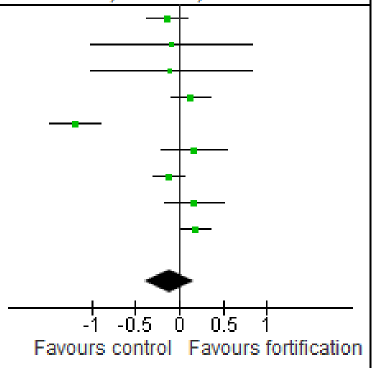

Figure S26 Effect of MMN fortification on Morbidities in children

Study or Subgroup	log[Risk Ratio]	SE	Weight	Risk Ratio IV, Random, 95% CI
**3.52.1 Fever**				
Manger 2008	-0.083	0.111	8.7%	0.92 [0.74, 1.14]
Osei 2010	0.33	0.2	6.9%	1.39 [0.94, 2.06]
Phu 2010 (a)	0.438	0.062	9.4%	1.55 [1.37, 1.75]
Phu 2010 (b)	0.425	0.063	9.4%	1.53 [1.35, 1.73]
**Subtotal (95% CI)**			**34.3%**	**1.33 [1.07, 1.67]**
Heterogeneity: Tau² = 0.04; Chi² = 18.60, df = 3 (P = 0.0003); I² = 84%				
Test for overall effect: Z = 2.54 (P = 0.01)				
**3.52.2 Respiratory Illness**				
Manger 2008	-0.186	0.065	9.3%	0.83 [0.73, 0.94]
Manno 2011	0.501	0.225	6.4%	1.65 [1.06, 2.57]
Osei 2010	-0.01	0.199	6.9%	0.99 [0.67, 1.46]
Phu 2010 (a)	0.27	0.062	9.4%	1.31 [1.16, 1.48]
Phu 2010 (b)	0.139	0.066	9.3%	1.15 [1.01, 1.31]
**Subtotal (95% CI)**			**41.4%**	**1.12 [0.90, 1.40]**
Heterogeneity: Tau² = 0.05; Chi² = 30.60, df = 4 (P < 0.00001); I² = 87%				
Test for overall effect: Z = 1.04 (P = 0.30)				
**3.52.3 UTI**				
Manno 2011	-0.843	0.365	4.1%	0.43 [0.21, 0.88]
**Subtotal (95% CI)**			**4.1%**	**0.43 [0.21, 0.88]**
Heterogeneity: Not applicable				
Test for overall effect: Z = 2.31 (P = 0.02)				
**3.52.4 Diarrheal Episodes**				
Manger 2008	-0.105	0.215	6.6%	0.90 [0.59, 1.37]
Osei 2010	-1.609	0.407	3.6%	0.20 [0.09, 0.44]
Phu 2010 (a)	1.258	0.214	6.7%	3.52 [2.31, 5.35]
Phu 2010 (b)	1.28	0.45	3.2%	3.60 [1.49, 8.69]
**Subtotal (95% CI)**			**20.1%**	**1.24 [0.39, 3.98]**
Heterogeneity: Tau² = 1.30; Chi² = 49.78, df = 3 (P < 0.00001); I² = 94%				
Test for overall effect: Z = 0.36 (P = 0.72)				
**Total (95% CI)**			**100.0%**	**1.19 [0.98, 1.44]**
Heterogeneity: Tau² = 0.10; Chi² = 134.68, df = 13 (P < 0.00001); I² = 90%				
Test for overall effect: Z = 1.77 (P = 0.08)				
Test for subgroup differences: Chi² = 8.97, df = 3 (P = 0.03), I² = 66.5%				

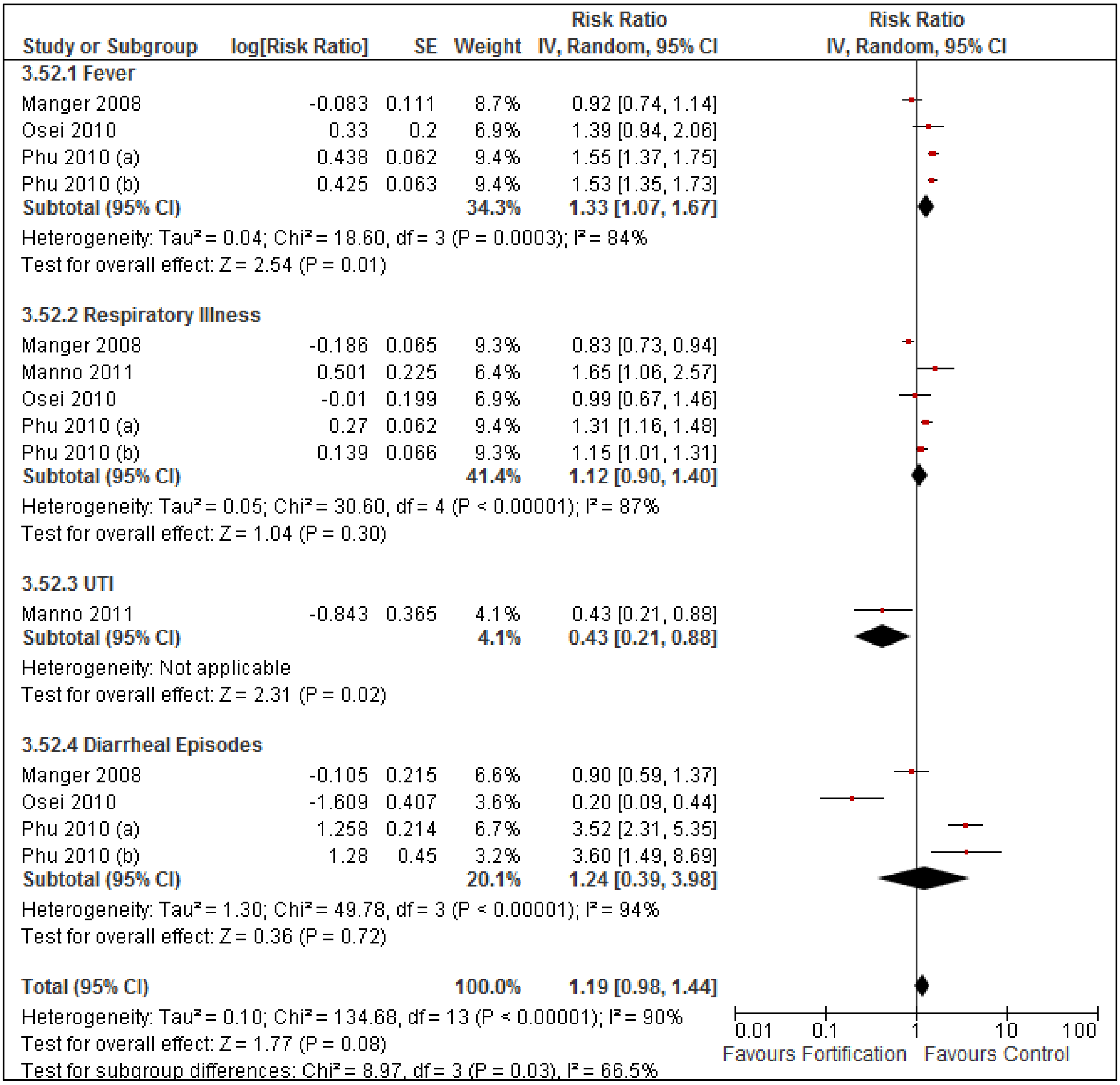

Figure S27 Effect of iron fortification on serum hemoglobin levels in women

Study or Subgroup	Fortification Mean	SD	Total	Control Mean	SD	Total	Weight	Std. Mean Difference IV, Random, 95% CI
**2.16.1 Healthy**								
Biebinger 2009	129	9	63	125	11	61	7.6%	0.40 [0.04, 0.75]
Chen 2005 WRA	127.6	9.57	505	122.6	9.7	480	8.5%	0.52 [0.39, 0.65]
Hotz 2008	142	1.1	75	138	1.28	70	6.6%	3.34 [2.83, 3.85]
Karl 2010	132	8	53	127	11	58	7.4%	0.51 [0.13, 0.89]
Sadighi 2008	135	14	556	129	14	294	8.5%	0.43 [0.29, 0.57]
Thuy 2005	131.1	11.5	199	128.6	12.27	189	8.3%	0.21 [0.01, 0.41]
Zimmerman 2005 ElectroFe	124	12	52	121	12	18	6.5%	0.25 [-0.29, 0.78]
Zimmerman 2005 FeSulphate	125	9	56	121	12	19	6.5%	0.40 [-0.12, 0.93]
Zimmerman 2005 Hyd Fe	122	12	52	121	12	19	6.5%	0.08 [-0.44, 0.61]
**Subtotal (95% CI)**			**1611**			**1208**	**66.5%**	**0.66 [0.30, 1.02]**
Heterogeneity: Tau² = 0.26; Chi² = 133.01, df = 8 (P < 0.00001); I² = 94%								
Test for overall effect: Z = 3.59 (P = 0.0003)								
**2.16.2 Deficient**								
Andersson 2010 MFP	13.5	0.7	37	13.3	0.9	19	6.4%	0.26 [-0.30, 0.81]
Andersson 2010 NaFeEDTA	13.6	0.9	30	13.3	0.9	19	6.2%	0.33 [-0.25, 0.91]
Blanco-Rojo 2011	13.7	0.7	23	13.1	0.7	18	5.8%	0.84 [0.19, 1.49]
Blanco-Rojo 2012	136	8	64	132	8	58	7.5%	0.50 [0.14, 0.86]
Thuy 2003	116.3	8.7	64	107.6	11	72	7.6%	0.87 [0.51, 1.22]
**Subtotal (95% CI)**			**218**			**186**	**33.5%**	**0.59 [0.34, 0.83]**
Heterogeneity: Tau² = 0.02; Chi² = 5.37, df = 4 (P = 0.25); I² = 26%								
Test for overall effect: Z = 4.73 (P < 0.00001)								
**Total (95% CI)**			**1829**			**1394**	**100.0%**	**0.62 [0.36, 0.89]**
Heterogeneity: Tau² = 0.22; Chi² = 139.42, df = 13 (P < 0.00001); I² = 91%								
Test for overall effect: Z = 4.55 (P < 0.00001)								
Test for subgroup differences: Chi² = 0.11, df = 1 (P = 0.74), I² = 0%								

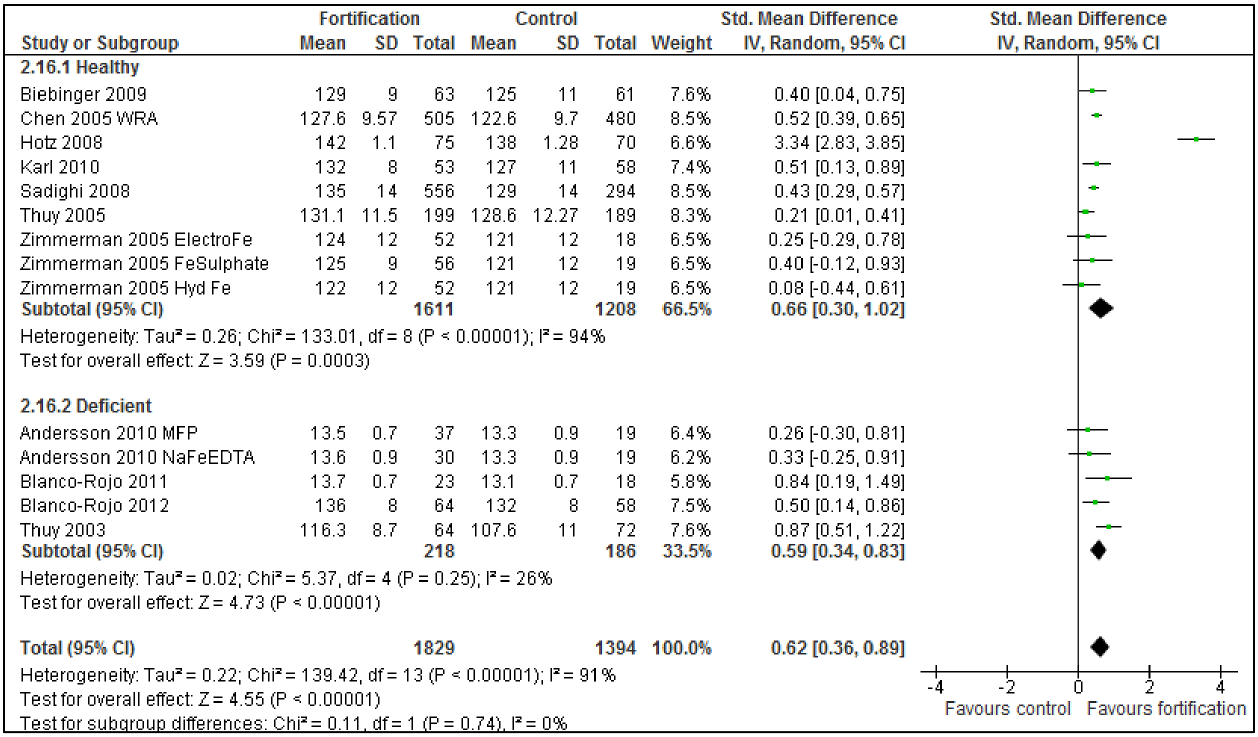

Figure S28 Effect of iron fortification on anemia prevalence in women

Study or Subgroup	log[Risk Ratio]	SE	Fortification Total	Control Total	Weight	Risk Ratio IV, Random, 95% CI
**2.17.1 Healthy**						
Karl 2010	-0.6763	0.4596	59	65	8.9%	0.51 [0.21, 1.25]
Sadighi 2008	-1.1282	0.2739	545	291	16.2%	0.32 [0.19, 0.55]
Zimmerman 2005 ElectroFe	0.1229	0.246	52	18	17.7%	1.13 [0.70, 1.83]
Zimmerman 2005 FeSulphate	-0.5108	0.3124	56	19	14.3%	0.60 [0.33, 1.11]
Zimmerman 2005 Hyd Fe	-0.1054	0.2643	56	19	16.7%	0.90 [0.54, 1.51]
**Subtotal (95% CI)**			**768**	**412**	**73.7%**	**0.65 [0.40, 1.04]**
Heterogeneity: Tau² = 0.20; Chi² = 13.31, df = 4 (P = 0.010); I² = 70%						
Test for overall effect: Z = 1.78 (P = 0.07)						
**2.17.2 Deficient**						
Thuy 2003	-0.3034	0.0996	64	72	26.3%	0.74 [0.61, 0.90]
**Subtotal (95% CI)**			**64**	**72**	**26.3%**	**0.74 [0.61, 0.90]**
Heterogeneity: Not applicable						
Test for overall effect: Z = 3.05 (P = 0.002)						
**Total (95% CI)**			**832**	**484**	**100.0%**	**0.68 [0.49, 0.93]**
Heterogeneity: Tau² = 0.09; Chi² = 13.57, df = 5 (P = 0.02); I² = 63%						
Test for overall effect: Z = 2.37 (P = 0.02)						
Test for subgroup differences: Chi² = 0.25, df = 1 (P = 0.62), I² = 0%						

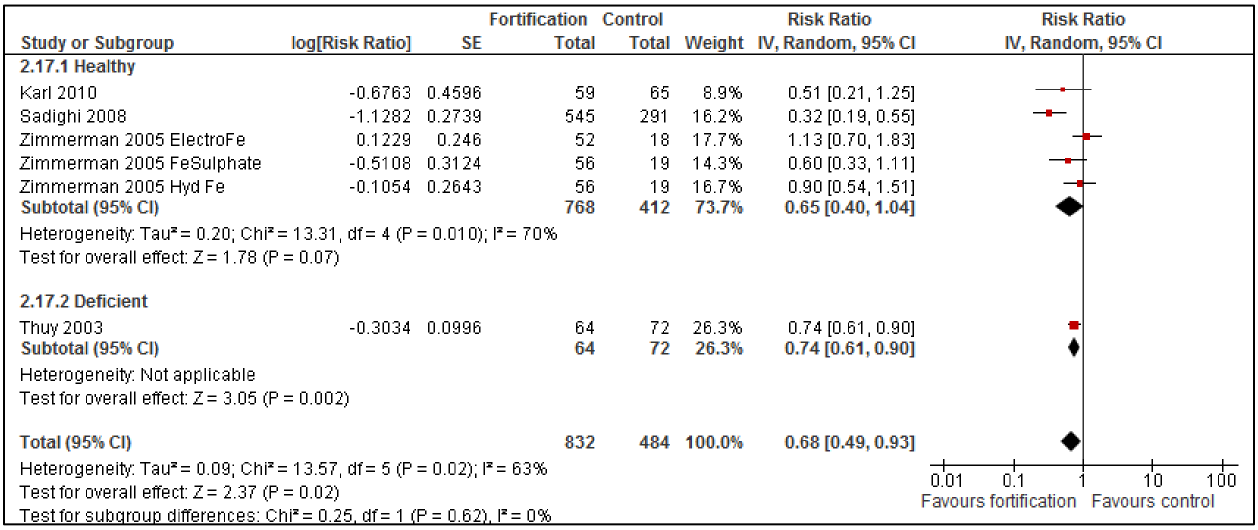

Figure S29 Effect of iron fortification on serum ferritin levels in women

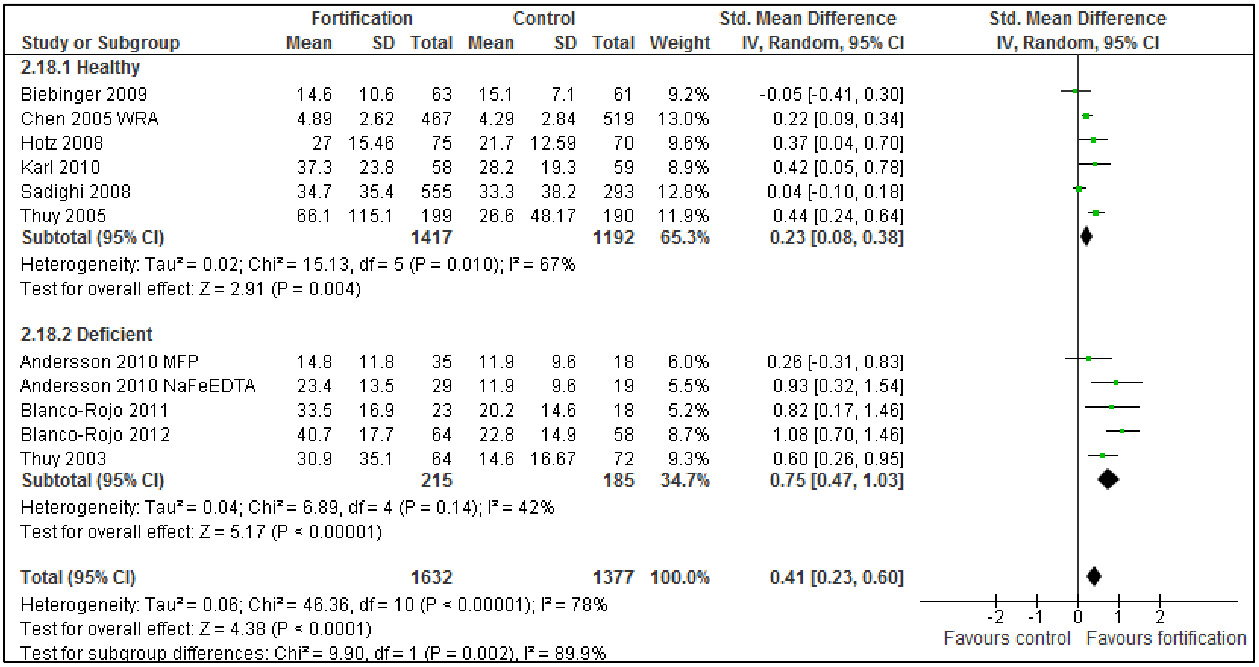

Study or Subgroup	Fortification Mean	Fortification SD	Fortification Total	Control Mean	Control SD	Control Total	Weight	Std. Mean Difference IV, Random, 95% CI
**2.18.1 Healthy**								
Biebinger 2009	14.6	10.6	63	15.1	7.1	61	9.2%	-0.05 [-0.41, 0.30]
Chen 2005 WRA	4.89	2.62	467	4.29	2.84	519	13.0%	0.22 [0.09, 0.34]
Hotz 2008	27	15.46	75	21.7	12.59	70	9.6%	0.37 [0.04, 0.70]
Karl 2010	37.3	23.8	58	28.2	19.3	59	8.9%	0.42 [0.05, 0.78]
Sadighi 2008	34.7	35.4	555	33.3	38.2	293	12.8%	0.04 [-0.10, 0.18]
Thuy 2005	66.1	115.1	199	26.6	48.17	190	11.9%	0.44 [0.24, 0.64]
**Subtotal (95% CI)**			**1417**			**1192**	**65.3%**	**0.23 [0.08, 0.38]**
Heterogeneity: Tau² = 0.02; Chi² = 15.13, df = 5 (P = 0.010); I² = 67%								
Test for overall effect: Z = 2.91 (P = 0.004)								
**2.18.2 Deficient**								
Andersson 2010 MFP	14.8	11.8	35	11.9	9.6	18	6.0%	0.26 [-0.31, 0.83]
Andersson 2010 NaFeEDTA	23.4	13.5	29	11.9	9.6	19	5.5%	0.93 [0.32, 1.54]
Blanco-Rojo 2011	33.5	16.9	23	20.2	14.6	18	5.2%	0.82 [0.17, 1.46]
Blanco-Rojo 2012	40.7	17.7	64	22.8	14.9	58	8.7%	1.08 [0.70, 1.46]
Thuy 2003	30.9	35.1	64	14.6	16.67	72	9.3%	0.60 [0.26, 0.95]
**Subtotal (95% CI)**			**215**			**185**	**34.7%**	**0.75 [0.47, 1.03]**
Heterogeneity: Tau² = 0.04; Chi² = 6.89, df = 4 (P = 0.14); I² = 42%								
Test for overall effect: Z = 5.17 (P < 0.00001)								
**Total (95% CI)**			**1632**			**1377**	**100.0%**	**0.41 [0.23, 0.60]**
Heterogeneity: Tau² = 0.06; Chi² = 46.36, df = 10 (P < 0.00001); I² = 78%								
Test for overall effect: Z = 4.38 (P < 0.0001)								
Test for subgroup differences: Chi² = 9.90, df = 1 (P = 0.002), I² = 89.9%								

Figure S30 Effect of folate fortification on neural tube defects

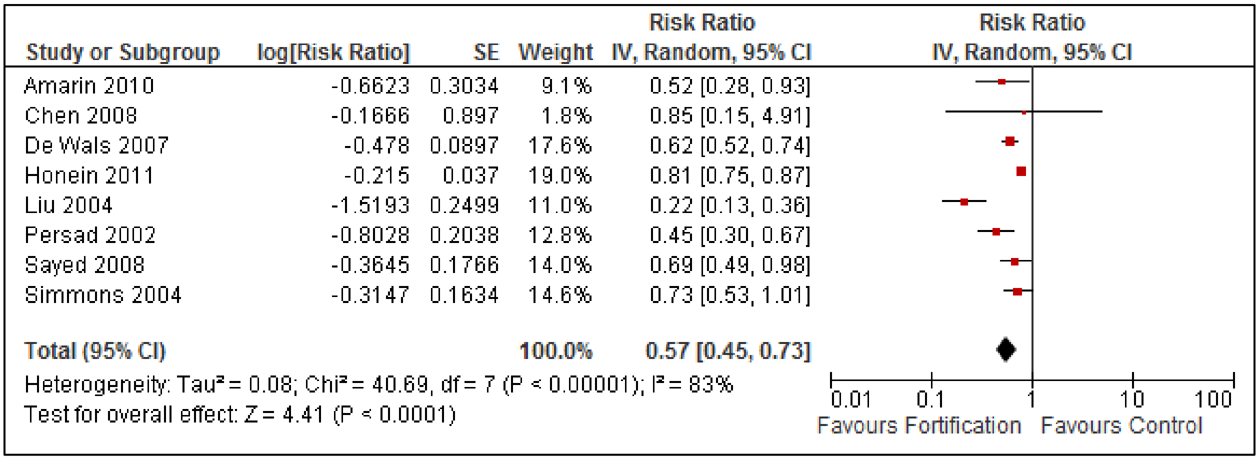

Study or Subgroup	log[Risk Ratio]	SE	Weight	Risk Ratio IV, Random, 95% CI
Amarin 2010	-0.6623	0.3034	9.1%	0.52 [0.28, 0.93]
Chen 2008	-0.1666	0.897	1.8%	0.85 [0.15, 4.91]
De Wals 2007	-0.478	0.0897	17.6%	0.62 [0.52, 0.74]
Honein 2011	-0.215	0.037	19.0%	0.81 [0.75, 0.87]
Liu 2004	-1.5193	0.2499	11.0%	0.22 [0.13, 0.36]
Persad 2002	-0.8028	0.2038	12.8%	0.45 [0.30, 0.67]
Sayed 2008	-0.3645	0.1766	14.0%	0.69 [0.49, 0.98]
Simmons 2004	-0.3147	0.1634	14.6%	0.73 [0.53, 1.01]
**Total (95% CI)**			**100.0%**	**0.57 [0.45, 0.73]**
Heterogeneity: Tau² = 0.08; Chi² = 40.69, df = 7 (P < 0.00001); I² = 83%				
Test for overall effect: Z = 4.41 (P < 0.0001)				

Figure S31 Effect of folate fortification on Spina Bifida

Study or Subgroup	log[Risk Ratio]	SE	Weight	Risk Ratio IV, Random, 95% CI
Boulet 2008	-0.0348	0.426	1.7%	0.97 [0.42, 2.23]
Canfield 2005	-0.4197	0.0398	14.5%	0.66 [0.61, 0.71]
Chen 2008	-0.1032	0.1064	10.2%	0.90 [0.73, 1.11]
De Wals 2007	-0.755	0.0823	11.9%	0.47 [0.40, 0.55]
Honein 2011	-0.2623	0.045	14.2%	0.77 [0.70, 0.84]
Lopez-Cmello 2005	-0.6713	0.1568	7.3%	0.51 [0.38, 0.69]
orioli 2011	-0.497	0.0642	13.1%	0.61 [0.54, 0.69]
Persad 2002	-0.9513	0.2743	3.5%	0.39 [0.23, 0.66]
Sayed 2008	-0.5378	0.2314	4.5%	0.58 [0.37, 0.92]
Simmons 2004	-0.5798	0.2114	5.1%	0.56 [0.37, 0.85]
Williams 2002	-0.371	0.046	14.2%	0.69 [0.63, 0.76]
**Total (95% CI)**			**100.0%**	**0.64 [0.57, 0.71]**

Heterogeneity: $Tau^2 = 0.02$; $Chi^2 = 47.52$, df = 10 (P < 0.00001); $I^2 = 79\%$
Test for overall effect: Z = 7.79 (P < 0.00001)

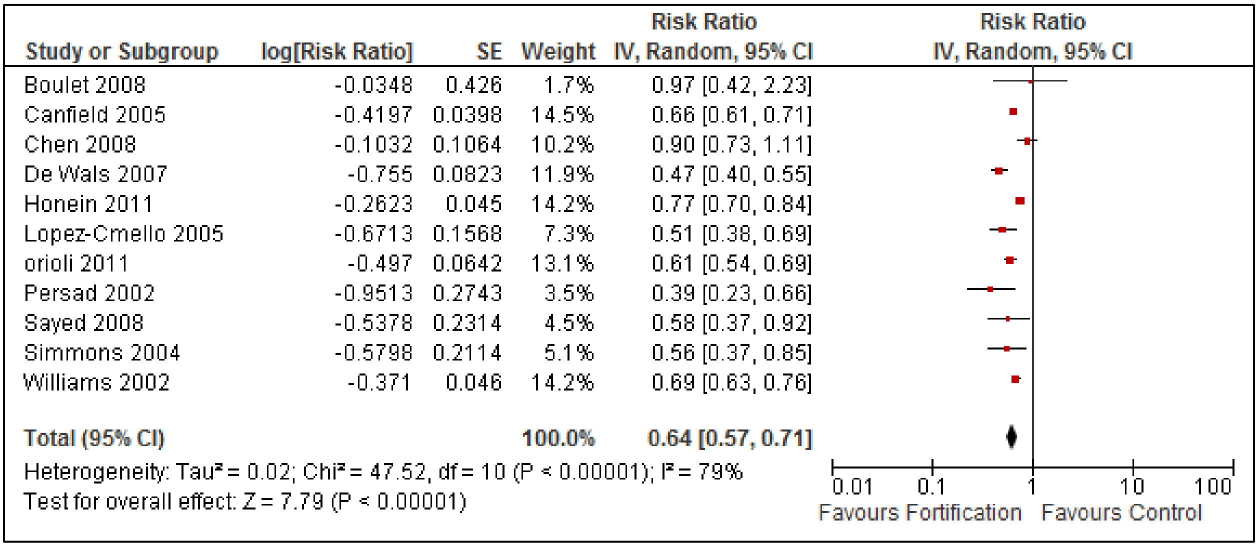

Figure S32 Effect of folate fortification on anencephaly

Study or Subgroup	log[Risk Ratio]	SE	Weight	Risk Ratio IV, Random, 95% CI
Boulet 2008	-0.2212	0.0533	15.9%	0.80 [0.72, 0.89]
Canfield 2005	-0.17118	0.0554	15.8%	0.84 [0.76, 0.94]
Chen 2008	-0.478	0.0897	12.7%	0.62 [0.52, 0.74]
De Wals 2007	-0.2663	0.1351	9.1%	0.77 [0.59, 1.00]
Honein 2011	-0.1156	0.065	14.9%	0.89 [0.78, 1.01]
Lopez-Cmello 2005	-0.9476	0.1862	6.3%	0.39 [0.27, 0.56]
Persad 2002	-0.7695	0.3598	2.2%	0.46 [0.23, 0.94]
Sayed 2008	-0.1156	0.2986	3.1%	0.89 [0.50, 1.60]
Simmons 2004	-0.0513	0.243	4.3%	0.95 [0.59, 1.53]
Williams 2002	-0.1917	0.0576	15.6%	0.83 [0.74, 0.92]
**Total (95% CI)**			**100.0%**	**0.76 [0.68, 0.85]**

Heterogeneity: $Tau^2 = 0.02$; $Chi^2 = 30.16$, df = 9 (P = 0.0004); $I^2 = 70\%$
Test for overall effect: Z = 4.83 (P < 0.00001)

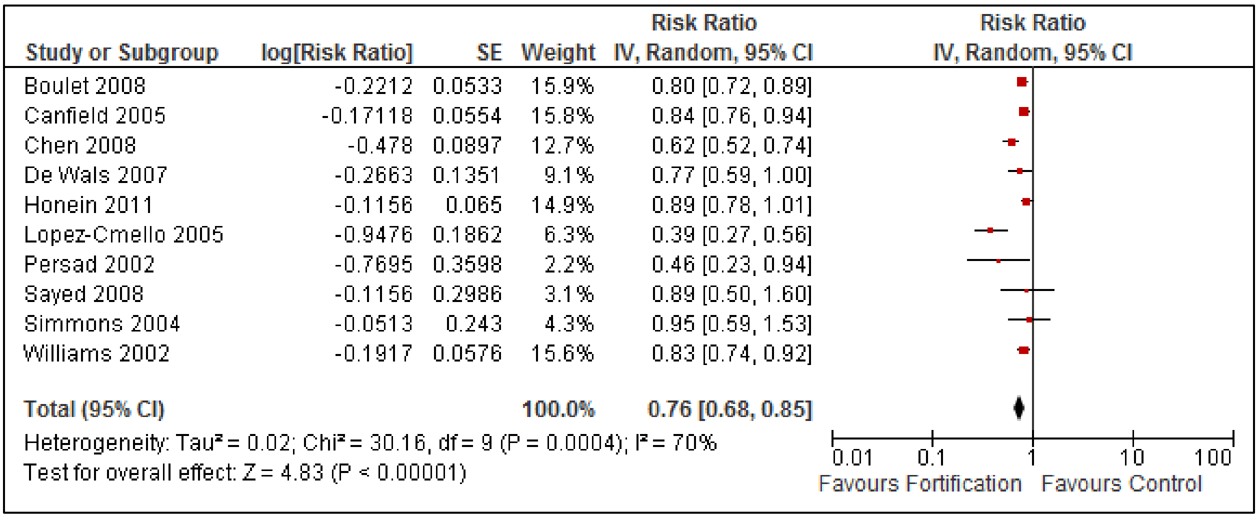

Figure S33 Effect of folate fortification on twinning

Study or Subgroup	log[Risk Ratio]	SE	Weight	Risk Ratio IV, Random, 95% CI
Kucik 2004	0.207	0.026	22.3%	1.23 [1.17, 1.29]
Lawrence 2004	0.0385	0.0381	21.6%	1.04 [0.96, 1.12]
Nazer 2006	0.2776	0.152	11.2%	1.32 [0.98, 1.78]
Shaw 2003	0	0.026	22.3%	1.00 [0.95, 1.05]
Waller 2003	-0.114	0.0186	22.6%	0.89 [0.86, 0.93]
**Total (95% CI)**			**100.0%**	**1.06 [0.92, 1.22]**

Heterogeneity: Tau² = 0.02; Chi² = 105.27, df = 4 (P < 0.00001); I² = 96%
Test for overall effect: Z = 0.84 (P = 0.40)

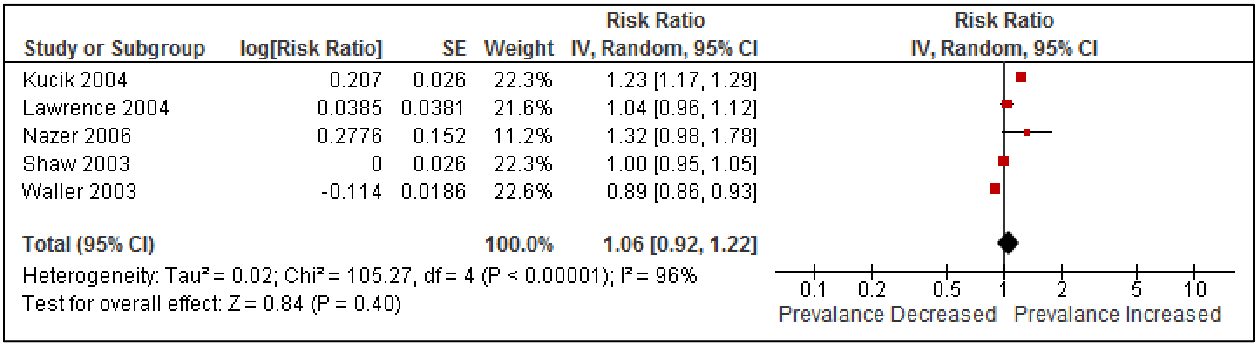

Figure S34 Effect of folate fortification on serum folate levels

Study or Subgroup	Experimental Mean	Experimental SD	Experimental Total	Control Mean	Control SD	Control Total	Weight	Std. Mean Difference IV, Random, 95% CI
Chen 2004 (rural)	12.5	18.6	300	9.6	7.03	190	25.0%	0.19 [0.01, 0.37]
Chen 2004 (urban)	15.8	9.09	288	10.1	5.83	204	25.0%	0.72 [0.54, 0.90]
Hertrampf 2003	37.2	9.5	605	9.7	4.32	605	25.0%	3.72 [3.54, 3.91]
Liu 2004	18.1	5.83	204	13.5	4.67	233	25.0%	0.88 [0.68, 1.07]
**Total (95% CI)**			**1397**			**1232**	**100.0%**	**1.38 [-0.20, 2.95]**

Heterogeneity: Tau² = 2.57; Chi² = 844.84, df = 3 (P < 0.00001); I² = 100%
Test for overall effect: Z = 1.72 (P = 0.09)

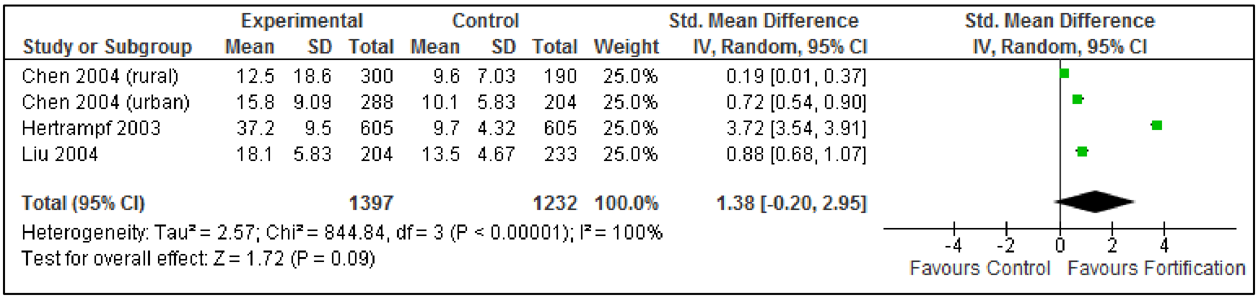

Figure S35 Effect of folate fortification on RBC folate levels

Study or Subgroup	Experimental Mean	Experimental SD	Experimental Total	Control Mean	Control SD	Control Total	Weight	Std. Mean Difference IV, Random, 95% CI
Bar-oz 2008	462	5,520	1537	226	1,012	221	33.4%	0.05 [-0.10, 0.19]
Hertrampf 2003	707	179	605	290	102	605	33.3%	2.86 [2.70, 3.02]
Liu 2004	818	255.05	204	625	186.91	233	33.3%	0.87 [0.67, 1.07]
**Total (95% CI)**			**2346**			**1059**	**100.0%**	**1.26 [-0.49, 3.01]**

Heterogeneity: Tau² = 2.38; Chi² = 679.83, df = 2 (P < 0.00001); I² = 100%
Test for overall effect: Z = 1.41 (P = 0.16)

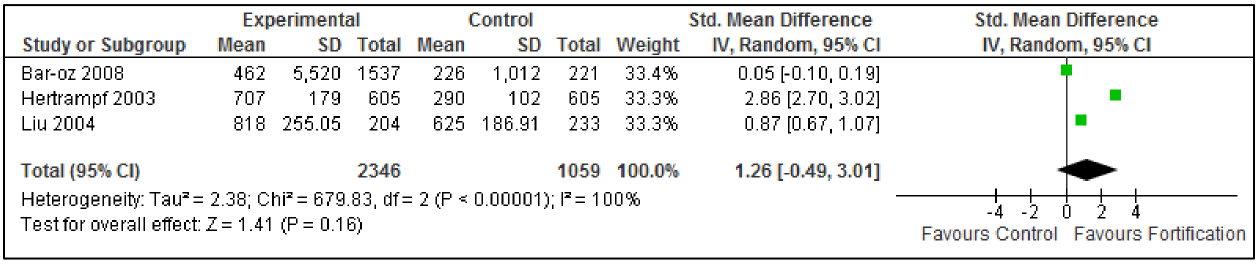

Figure S36 Effect of folate fortification on folate deficiency

Study or Subgroup	log[Risk Ratio]	SE	Weight	Risk Ratio IV, Random, 95% CI
Chen 2004 (rural)	-1.2483	0.2586	45.3%	0.29 [0.17, 0.48]
Chen 2004 (urban)	-2.2402	0.477	39.1%	0.11 [0.04, 0.27]
Hertrampf 2003	-4.9779	1.4197	15.6%	0.01 [0.00, 0.11]
**Total (95% CI)**			**100.0%**	**0.11 [0.03, 0.41]**

Heterogeneity: Tau² = 0.96; Chi² = 9.28, df = 2 (P = 0.010); I² = 78%
Test for overall effect: Z = 3.26 (P = 0.001)

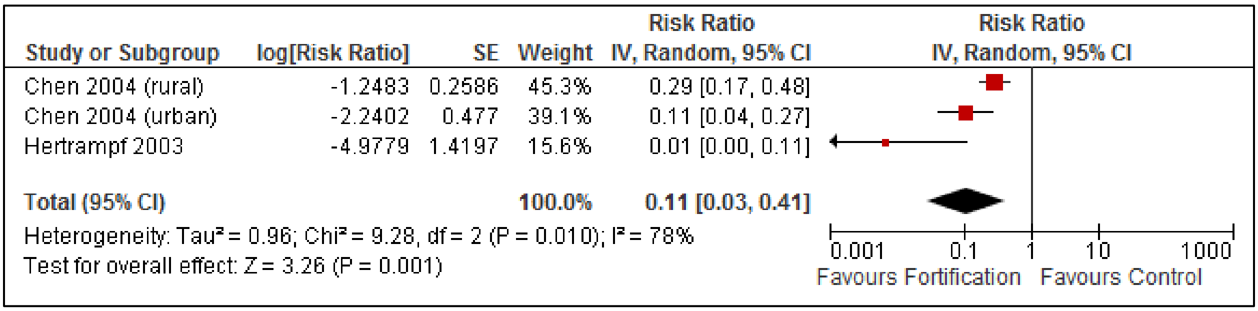

Figure S37 Effect of iodine fortification on urinary iodine concentration in women

Study or Subgroup	Fortification Mean	Fortification SD	Fortification Total	Control Mean	Control SD	Control Total	Weight	Std. Mean Difference IV, Random, 95% CI
Burgess 2007	86	4.14	229	76	3.16	285	25.0%	2.75 [2.51, 2.99]
Moleti 2011	76.3	7.13	105	52.2	7.76	160	25.0%	3.20 [2.83, 3.57]
Rasmussen 2008	128	3.89	2516	78	3.26	3011	25.0%	14.04 [13.77, 14.31]
Seal 2007	105	3.72	401	75	2.45	124	25.0%	8.65 [8.09, 9.21]
**Total (95% CI)**			**3251**			**3580**	**100.0%**	**7.16 [1.00, 13.31]**

Heterogeneity: Tau² = 39.41; Chi² = 4289.57, df = 3 (P < 0.00001); I² = 100%
Test for overall effect: Z = 2.28 (P = 0.02)

Std. Mean Difference IV, Random, 95% CI

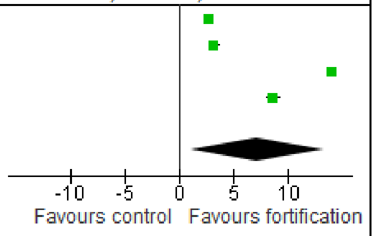

Figure S38 Effect of Iodine fortification on Hypothyroidism in women

Study or Subgroup	log[Risk Ratio]	SE	Weight	Risk Ratio IV, Random, 95% CI
Pederson 2011	0.182	0.077	51.2%	1.20 [1.03, 1.40]
Verjberg 2007 (b)	0.693	0.261	17.3%	2.00 [1.20, 3.34]
Verjgerg 2007 (a)	0.531	0.271	16.4%	1.70 [1.00, 2.89]
Verjgerg 2007 (c)	0.336	0.285	15.2%	1.40 [0.80, 2.45]
**Total (95% CI)**			**100.0%**	**1.42 [1.11, 1.82]**

Heterogeneity: Tau² = 0.03; Chi² = 4.83, df = 3 (P = 0.18); I² = 38%
Test for overall effect: Z = 2.75 (P = 0.006)

Risk Ratio IV, Random, 95% CI

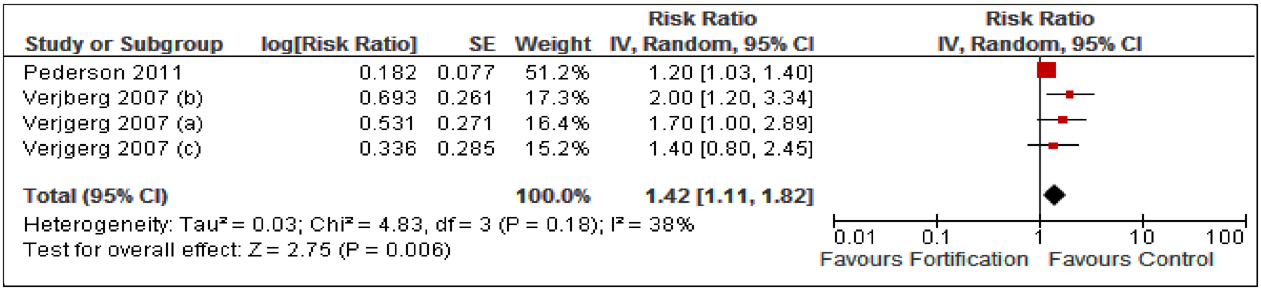

Figure S39 Effect of vitamin D and calcium fortification on serum vitamin D levels in post-menopausal women

Study or Subgroup	Fortification Mean	Fortification SD	Fortification Total	Control Mean	Control SD	Control Total	Weight	Std. Mean Difference IV, Random, 95% CI
**4.2.1 Calcium Only**								
Chee 2003	86.4	22	91	71.2	21.7	82	40.2%	0.69 [0.38, 1.00]
**Subtotal (95% CI)**			**91**			**82**	**40.2%**	**0.69 [0.38, 1.00]**
Heterogeneity: Not applicable								
Test for overall effect: Z = 4.41 (P < 0.0001)								
**4.2.3 Calcium and Vitamin D Only**								
Manios 2009	63.5	19.75	42	55.25	19.5	40	35.1%	0.42 [-0.02, 0.85]
Tenta 2010	68	21	20	38.25	15	20	24.6%	1.60 [0.88, 2.32]
**Subtotal (95% CI)**			**62**			**60**	**59.8%**	**0.97 [-0.18, 2.13]**
Heterogeneity: Tau² = 0.61; Chi² = 7.53, df = 1 (P = 0.006); I² = 87%								
Test for overall effect: Z = 1.65 (P = 0.10)								
**Total (95% CI)**			**153**			**142**	**100.0%**	**0.82 [0.30, 1.34]**

Heterogeneity: Tau² = 0.15; Chi² = 7.56, df = 2 (P = 0.02); I² = 74%
Test for overall effect: Z = 3.09 (P = 0.002)
Test for subgroup differences: Chi² = 0.21, df = 1 (P = 0.65), I² = 0%

Std. Mean Difference IV, Random, 95% CI

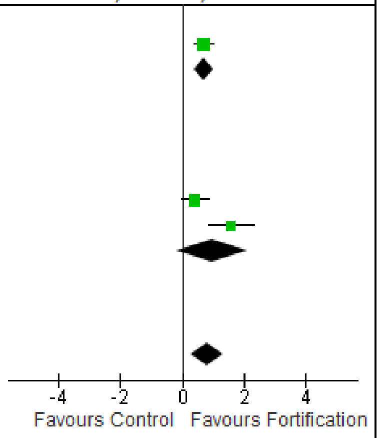

Figure S40 Effect of vitamin D and calcium fortification on serum vitamin D levels in women of reproductive age group

Study or Subgroup	Fortification Mean	SD	Total	Control Mean	SD	Total	Weight	Std. Mean Difference IV, Random, 95% CI
**4.4.2 Vitamin D Only**								
Green 2010	65	44.324	32	53	45.68	34	50.5%	0.26 [-0.22, 0.75]
**Subtotal (95% CI)**			**32**			**34**	**50.5%**	**0.26 [-0.22, 0.75]**
Heterogeneity: Not applicable								
Test for overall effect: Z = 1.06 (P = 0.29)								
**4.4.3 Calcium and Vitamin D Only**								
Kruger 2006	53.5	2.58	29	59.9	2.46	26	49.5%	-2.50 [-3.22, -1.78]
**Subtotal (95% CI)**			**29**			**26**	**49.5%**	**-2.50 [-3.22, -1.78]**
Heterogeneity: Not applicable								
Test for overall effect: Z = 6.82 (P < 0.00001)								
**Total (95% CI)**			**61**			**60**	**100.0%**	**-1.10 [-3.81, 1.60]**
Heterogeneity: Tau² = 3.72; Chi² = 39.08, df = 1 (P < 0.00001); I² = 97%								
Test for overall effect: Z = 0.80 (P = 0.42)								
Test for subgroup differences: Chi² = 39.08, df = 1 (P < 0.00001), I² = 97.4%								

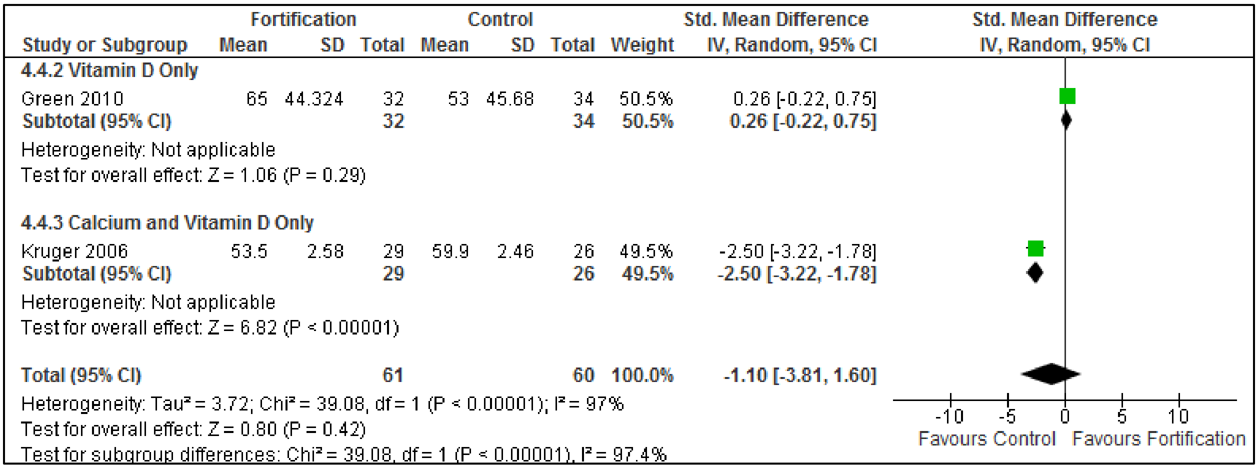

Figure S41 Effect of vitamin D and calcium fortification on serum PTH levels in post-menopausal women

Study or Subgroup	Favours Intervention Mean	SD	Total	Favours Control Mean	SD	Total	Weight	Std. Mean Difference IV, Random, 95% CI
**4.1.3 Calcium and Vitamin D only**								
Kruger 2010 (Indo)	4.43	0.23	27	5.95	0.23	29	23.2%	-6.52 [-7.87, -5.16]
Kruger 2010 (Phili)	3.75	0.27	30	4.54	0.27	30	25.3%	-2.89 [-3.62, -2.15]
Manios 2009	3.48	1.8	42	4.67	1.8	40	25.9%	-0.65 [-1.10, -0.21]
Tenta 2010	4.05	2.08	20	5.1	2.2	20	25.6%	-0.48 [-1.11, 0.15]
**Subtotal (95% CI)**			**119**			**119**	**100.0%**	**-2.53 [-4.42, -0.65]**
Heterogeneity: Tau² = 3.51; Chi² = 89.14, df = 3 (P < 0.00001); I² = 97%								
Test for overall effect: Z = 2.64 (P = 0.008)								
**Total (95% CI)**			**119**			**119**	**100.0%**	**-2.53 [-4.42, -0.65]**
Heterogeneity: Tau² = 3.51; Chi² = 89.14, df = 3 (P < 0.00001); I² = 97%								
Test for overall effect: Z = 2.64 (P = 0.008)								
Test for subgroup differences: Not applicable								

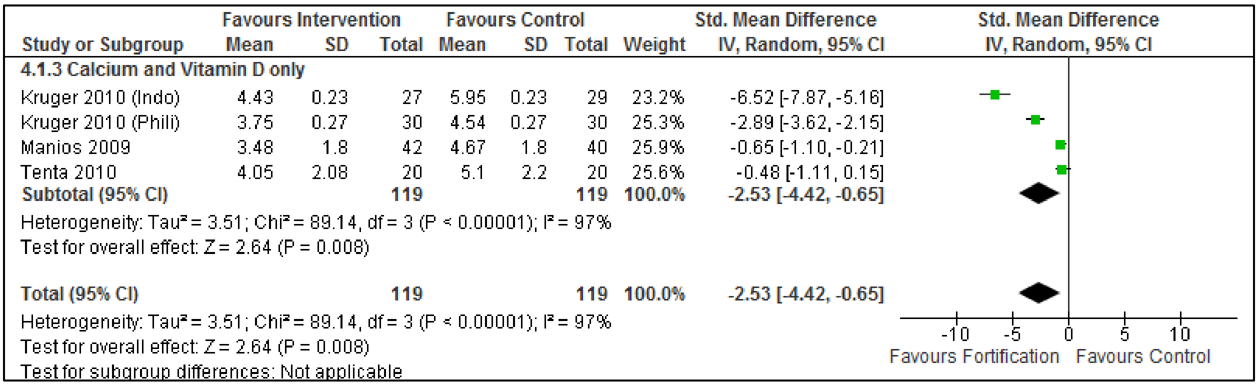

Figure S42 Effect of vitamin D and calcium fortification on serum PTH levels in women of reproductive age

Study or Subgroup	Favours Intervention Mean	SD	Total	Favours Control Mean	SD	Total	Weight	Std. Mean Difference IV, Random, 95% CI
**4.3.2 Vitamin D Only**								
Green 2010	2.9	1.662	32	2.9	1.203	37	43.5%	0.00 [-0.47, 0.47]
Natri 2006 (Rye)	2.08	0.8	11	2.13	0.65	9	12.5%	-0.06 [-0.95, 0.82]
Natri 2006 (Wheat)	2.46	0.2	10	2.13	0.65	9	11.2%	0.67 [-0.26, 1.60]
**Subtotal (95% CI)**			**53**			**55**	**67.3%**	**0.10 [-0.28, 0.48]**
Heterogeneity: Tau² = 0.00; Chi² = 1.75, df = 2 (P = 0.42); I² = 0%								
Test for overall effect: Z = 0.51 (P = 0.61)								
**4.3.3 Calcium and Vitamin D only**								
Kruger 2006	1.67	0.087	26	1.69	0.084	26	32.7%	-0.23 [-0.78, 0.32]
**Subtotal (95% CI)**			**26**			**26**	**32.7%**	**-0.23 [-0.78, 0.32]**
Heterogeneity: Not applicable								
Test for overall effect: Z = 0.83 (P = 0.41)								
**Total (95% CI)**			**79**			**81**	**100.0%**	**-0.01 [-0.32, 0.30]**
Heterogeneity: Tau² = 0.00; Chi² = 2.70, df = 3 (P = 0.44); I² = 0%								
Test for overall effect: Z = 0.05 (P = 0.96)								
Test for subgroup differences: Chi² = 0.95, df = 1 (P = 0.33), I² = 0%								

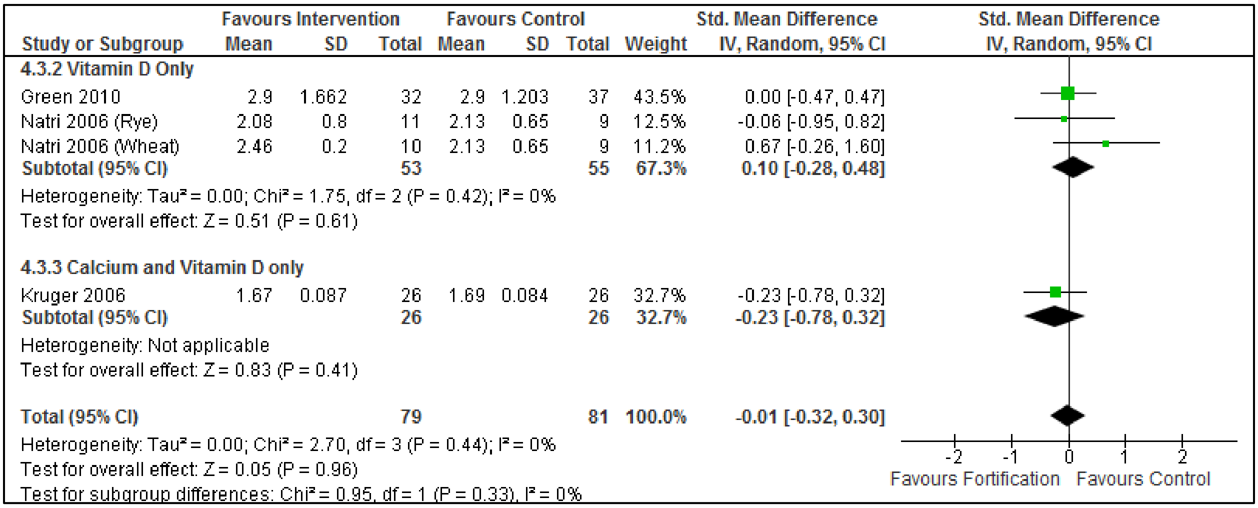

Figure S43 Effect of vitamin D and calcium fortification on serum calcium levels in women of reproductive age

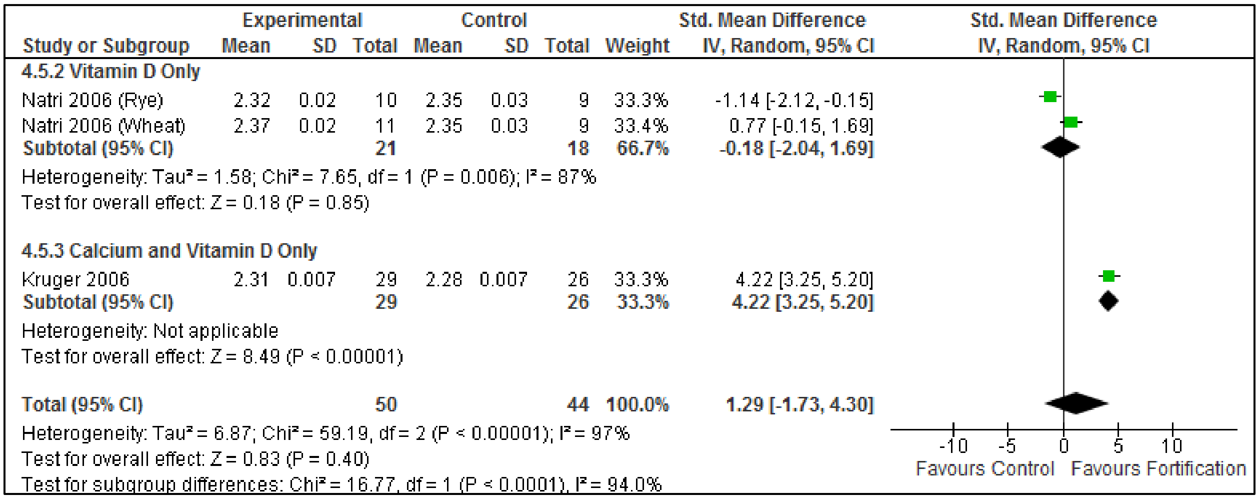

Study or Subgroup	Experimental Mean	Experimental SD	Experimental Total	Control Mean	Control SD	Control Total	Weight	Std. Mean Difference IV, Random, 95% CI
**4.5.2 Vitamin D Only**								
Natri 2006 (Rye)	2.32	0.02	10	2.35	0.03	9	33.3%	-1.14 [-2.12, -0.15]
Natri 2006 (Wheat)	2.37	0.02	11	2.35	0.03	9	33.4%	0.77 [-0.15, 1.69]
**Subtotal (95% CI)**			**21**			**18**	**66.7%**	**-0.18 [-2.04, 1.69]**
Heterogeneity: Tau² = 1.58; Chi² = 7.65, df = 1 (P = 0.006); I² = 87%								
Test for overall effect: Z = 0.18 (P = 0.85)								
**4.5.3 Calcium and Vitamin D Only**								
Kruger 2006	2.31	0.007	29	2.28	0.007	26	33.3%	4.22 [3.25, 5.20]
**Subtotal (95% CI)**			**29**			**26**	**33.3%**	**4.22 [3.25, 5.20]**
Heterogeneity: Not applicable								
Test for overall effect: Z = 8.49 (P < 0.00001)								
**Total (95% CI)**			**50**			**44**	**100.0%**	**1.29 [-1.73, 4.30]**
Heterogeneity: Tau² = 6.87; Chi² = 59.19, df = 2 (P < 0.00001); I² = 97%								
Test for overall effect: Z = 0.83 (P = 0.40)								
Test for subgroup differences: Chi² = 16.77, df = 1 (P < 0.0001), I² = 94.0%								

Figure S44 Effect of vitamin D and calcium fortification on CTx levels (bone resorption marker) in women

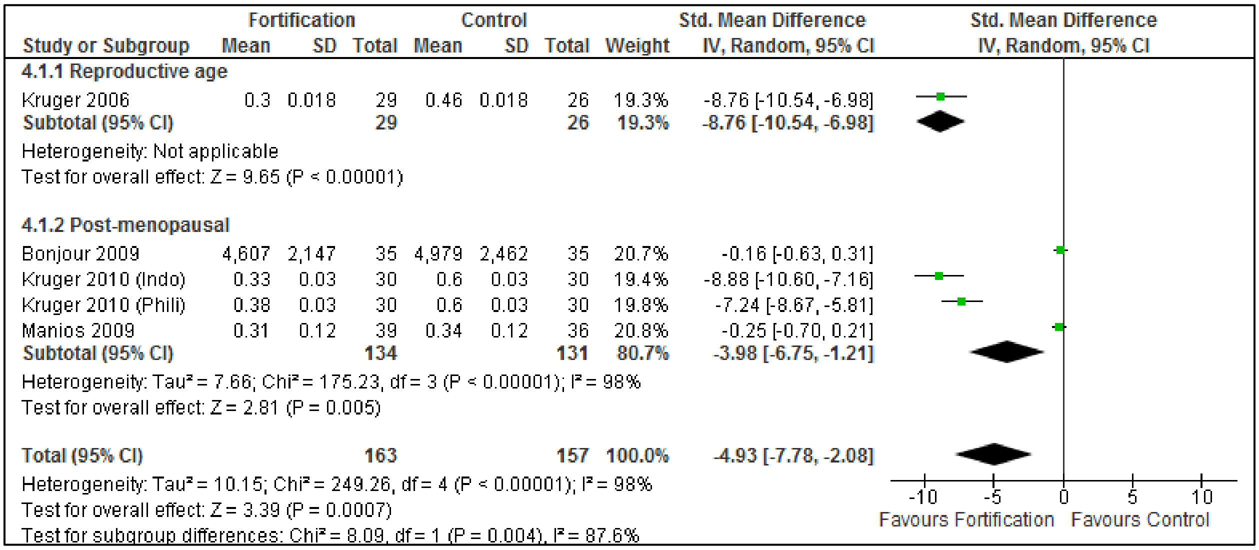

Study or Subgroup	Fortification Mean	Fortification SD	Fortification Total	Control Mean	Control SD	Control Total	Weight	Std. Mean Difference IV, Random, 95% CI
**4.1.1 Reproductive age**								
Kruger 2006	0.3	0.018	29	0.46	0.018	26	19.3%	-8.76 [-10.54, -6.98]
**Subtotal (95% CI)**			**29**			**26**	**19.3%**	**-8.76 [-10.54, -6.98]**
Heterogeneity: Not applicable								
Test for overall effect: Z = 9.65 (P < 0.00001)								
**4.1.2 Post-menopausal**								
Bonjour 2009	4,607	2,147	35	4,979	2,462	35	20.7%	-0.16 [-0.63, 0.31]
Kruger 2010 (Indo)	0.33	0.03	30	0.6	0.03	30	19.4%	-8.88 [-10.60, -7.16]
Kruger 2010 (Phili)	0.38	0.03	30	0.6	0.03	30	19.8%	-7.24 [-8.67, -5.81]
Manios 2009	0.31	0.12	39	0.34	0.12	36	20.8%	-0.25 [-0.70, 0.21]
**Subtotal (95% CI)**			**134**			**131**	**80.7%**	**-3.98 [-6.75, -1.21]**
Heterogeneity: Tau² = 7.66; Chi² = 175.23, df = 3 (P < 0.00001); I² = 98%								
Test for overall effect: Z = 2.81 (P = 0.005)								
**Total (95% CI)**			**163**			**157**	**100.0%**	**-4.93 [-7.78, -2.08]**
Heterogeneity: Tau² = 10.15; Chi² = 249.26, df = 4 (P < 0.00001); I² = 98%								
Test for overall effect: Z = 3.39 (P = 0.0007)								
Test for subgroup differences: Chi² = 8.09, df = 1 (P = 0.004), I² = 87.6%								

Figure S45 Effect of vitamin D and calcium fortification on P1NP levels (bone resorption marker) in women

	Fortification			Control				Std. Mean Difference
Study or Subgroup	Mean	SD	Total	Mean	SD	Total	Weight	IV, Random, 95% CI
**4.3.1 Reproductive age**								
Kruger 2006	42.1	2.13	29	53.4	2.08	26	24.7%	-5.29 [-6.44, -4.13]
**Subtotal (95% CI)**			**29**			**26**	**24.7%**	**-5.29 [-6.44, -4.13]**
Heterogeneity: Not applicable								
Test for overall effect: Z = 8.98 (P < 0.00001)								
**4.3.2 Post-menopausal**								
Bonjour 2009	98.3	76.1	35	82.4	46.5	35	25.4%	0.25 [-0.22, 0.72]
Kruger 2010 (Indo)	49.94	3.71	30	64.96	3.57	30	25.0%	-4.07 [-4.98, -3.16]
Kruger 2010 (Phili)	47.53	3.14	30	61.6	3.14	30	24.9%	-4.42 [-5.39, -3.46]
**Subtotal (95% CI)**			**95**			**95**	**75.3%**	**-2.73 [-6.12, 0.66]**
Heterogeneity: Tau² = 8.80; Chi² = 117.88, df = 2 (P < 0.00001); I² = 98%								
Test for overall effect: Z = 1.58 (P = 0.11)								
**Total (95% CI)**			**124**			**121**	**100.0%**	**-3.36 [-6.37, -0.35]**
Heterogeneity: Tau² = 9.21; Chi² = 160.00, df = 3 (P < 0.00001); I² = 98%								
Test for overall effect: Z = 2.19 (P = 0.03)								
Test for subgroup differences: Chi² = 1.96, df = 1 (P = 0.16), I² = 49.0%								

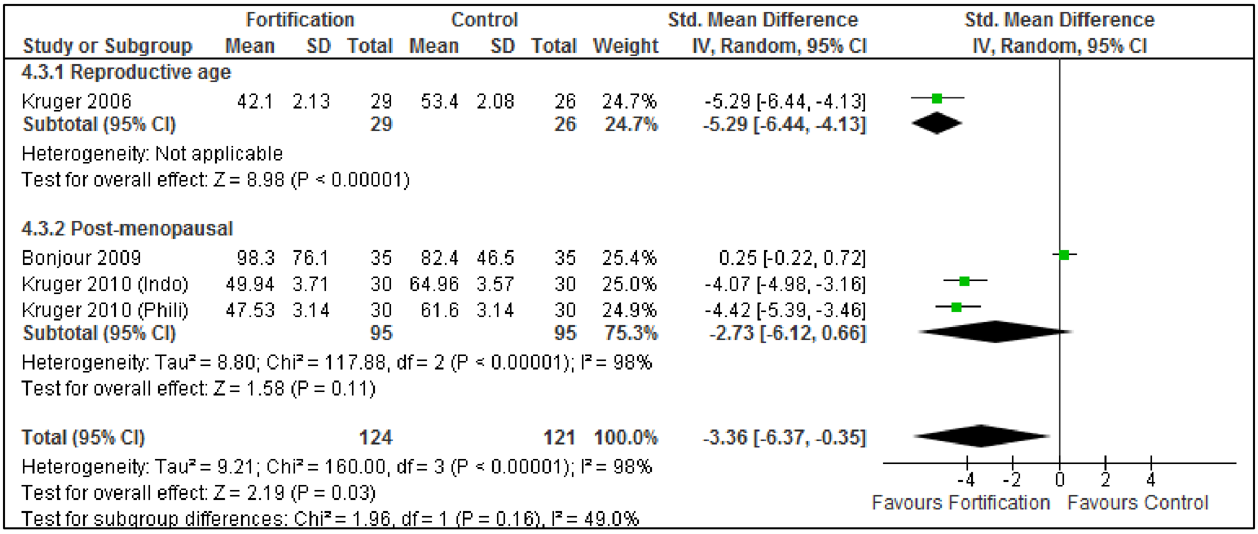

Figure S46 Effect of MMN fortification on serum ferritin levels in women

	Fortification			Control				Std. Mean Difference
Study or Subgroup	Mean	SD	Total	Mean	SD	Total	Weight	IV, Random, 95% CI
Huo 2011	0.86	0.16	269	0.76	0.26	247	42.5%	0.47 [0.29, 0.64]
Mardones 2007	24.3	9.76	365	20.05	8.03	333	57.5%	0.47 [0.32, 0.62]
**Total (95% CI)**			**634**			**580**	**100.0%**	**0.47 [0.36, 0.58]**
Heterogeneity: Tau² = 0.00; Chi² = 0.00, df = 1 (P = 0.96); I² = 0%								
Test for overall effect: Z = 8.08 (P < 0.00001)								

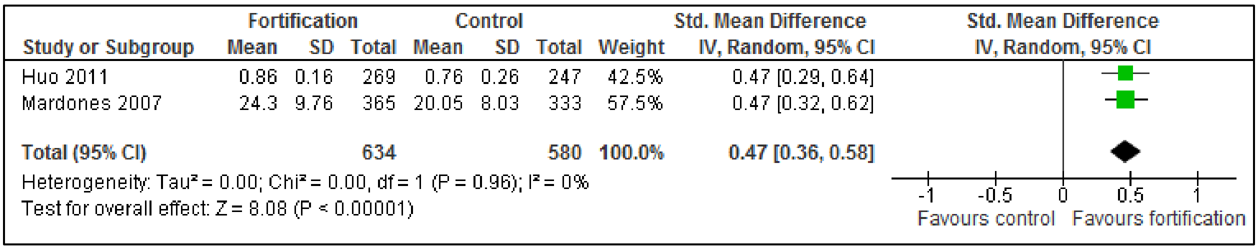

Figure S47 Effect of MMN fortification on serum zinc levels in women

	Fortification			Control				Std. Mean Difference
Study or Subgroup	Mean	SD	Total	Mean	SD	Total	Weight	IV, Random, 95% CI
Huo 2011	0.79	0.16	269	0.71	0.19	247	42.7%	0.46 [0.28, 0.63]
Mardones 2007	12.77	4.87	365	10.58	3.22	333	57.3%	0.53 [0.37, 0.68]
**Total (95% CI)**			**634**			**580**	**100.0%**	**0.50 [0.38, 0.61]**
Heterogeneity: Tau² = 0.00; Chi² = 0.34, df = 1 (P = 0.56); I² = 0%								
Test for overall effect: Z = 8.50 (P < 0.00001)								

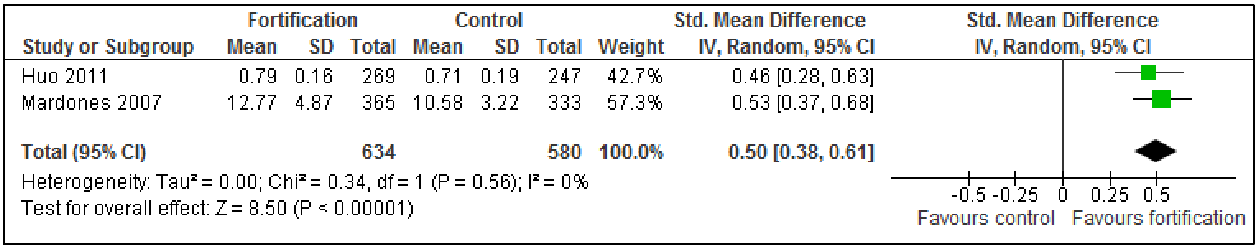

Supplement: Additional file 2 — Forest plots. [file 2046-4053-2-67-S2.pdf]
