# Supplementary material for: Micronutrient fortification of food and its impact on woman and child health: a systematic review
Source: Syst Rev. 2013 Aug 23;2:67. doi: 10.1186/2046-4053-2-67 (PMC3765883; doi:10.1186/2046-4053-2-67)
Supplement: Additional file 3 — Summary of findings and quality of outcomes. [file 2046-4053-2-67-S3.pdf]

Table S1. Effect of food fortification with various micronutrients on health outcomes in Children

Outcomes	Illustrative comparative risks* [95% CI]	Relative effect [95% CI]	No of Participants (studies)	Quality of the evidence (GRADE)	Comments
**Iron Fortification**					
**Hemoglobin Levels**	The mean hemoglobin levels in the intervention groups was **0.55 standard deviations higher** (0.34 to 0.76 higher)		6267 (30)	⊕⊕⊕⊖[1,2] **moderate**	**SMD: 0.55 [95% CI: 0.34, 0.76]**
**Serum Ferritin Levels**	The mean serum ferritin in the intervention groups was **0.91 standard deviations higher** (0.38 to 1.44 higher)		1507 (7)	⊕⊕⊕⊖[1,2] **moderate**	**SMD: 0.91 [95% CI: 0.38, 1.44]**
**Effect on Anemia**	Iron fortification reduced the prevalence of anemia by 45%	**RR: 0.55 [95% CI: 0.42, 0.72]**	3651 (17)	⊕⊕⊕⊖[1,2] **moderate**	
**Zinc Fortification**					
**Serum Zinc Levels**	The mean serum zinc levels in the intervention groups was **1.28 standard deviations higher** (0.56 to 2.01 higher)		683 (9)	⊕⊕⊕⊖[1,2] **moderate**	**SMD: 1.28 [95% CI: 0.56, 2.01]**
**Hemoglobin Levels**	The mean hemoglobin levels in the intervention groups was **0.11 standard deviations lower** (0.52 lower to 0.31 higher)		92 (3)	⊕⊕⊖⊖[1,2,4] **low**	**SMD: -0.11 [95% CI: -0.52, 0.31]**
**Serum Copper Levels**	The mean serum copper levels in the intervention groups was **0.57 standard deviations higher** (0.91 lower to 2.06 higher)		161 (4)	⊕⊖⊖⊖[1,2,4,5] **very low**	**SMD: 0.57 [95% CI: -0.91, 2.06]**
**Serum Alkaline Phosphatase Levels**	The mean serum alkaline phosphatase levels in the intervention groups was **0.94 standard deviations higher** (0.29 lower to 2.17		119	⊕⊕⊖⊖[1,2,4]	**SMD: 0.94 [95% CI: -0.29, 2.17]**

	higher)		(3)	**low**	
**Weight Gain**	The mean weight gain in the intervention groups was **0.50 standard deviations higher** (0.12 lower to 1.11 higher)		419 (6)	⊕⊕⊕⊖[1,2] **moderate**	**SMD: 0.50 [95% CI: -0.12, 1.11]**
**Height Growth**	The mean height growth levels in the intervention groups was **0.52 standard deviations higher** (0.01 to 1.04 higher)		451 (7)	⊕⊕⊕⊖[1,2] **moderate**	**SMD: 0.52 [95% CI: 0.01, 1.04]**
		**Iodine Fortification**			
**Serum thyroxin levels**	The mean serum thyroxin levels in the intervention groups was **0.45 standard deviations higher** (1.15 lower to 2.06 higher)		1131 (2)	⊕⊕⊖⊖[1,2,3] **low**	**SMD: 0.45 [95% CI: -1.15, 2.06]**
**Urinary iodine concentrations**	The mean urinary iodine concentration in the intervention groups was **6.39 standard deviations higher** (2.69 to 10.08 higher)		1016 (2)	⊕⊕⊕⊖[1,2] **moderate**	**SMD: 6.39 [95% CI: 2.69, 10.08]**
		**Vitamin A fortification**			
**Hemoglobin Levels**	The mean serum hemoglobin levels in the intervention groups was **0.48 standard deviations higher** (0.07 to 0.89 higher)		1538 (2)	⊕⊕⊖⊖[3,6] **low**	**SMD: 0.48 [95% CI: 0.07, 0.89]**
**Serum Vitamin A Concentration**	The mean serum vitamin A concentration in the intervention groups was **0.61 standard deviations higher** (0.39 to 0.83 higher)		2362 (3)	⊕⊕⊖⊖[3,6] **low**	**SMD: 0.61 [95% CI: 0.39, 0.83]**
**Vitamin A Deficiency**	Vitamin A fortification reduced prevalence of vitamin A deficiency by 61%	**RR: 0.39 [95% CI: 0.09, 1.74]**	1465 (2)	⊕⊕⊕⊖[6] **moderate**	

	**Calcium and Vitamin D fortification**				
**Serum PTH Levels**	The mean serum PTH levels in the intervention groups was **0.40 standard deviations lower** (0.56 to 0.24 lower)		637<br>(2)	⊕⊕⊝⊝[1,2,3]<br>**low**	**SMD: -0.40 [95% CI: -0.56, -0.24]**
**Serum Vitamin D Levels**	The mean serum vitamin D concentration in the intervention groups was 1**.23 standard deviations higher** (0.35 to 2.11 higher)		1119<br>(3)	⊕⊕⊕⊝[1,2]<br>**moderate**	**SMD: 1.23 [95% CI: 0.35, 2.11]**
**Serum Calcium Levels**	The mean serum calcium levels in the intervention groups was **0.40 standard deviations lower** (0.59 to 0.20 lower)		468<br>(1)	⊕⊕⊝⊝[1,2,3]<br>**low**	**SMD: -0.40 [95% CI: -0.59, -0.20]**
	**Multiple Micronutrient Fortification**				
**Hemoglobin Levels**	The mean hemoglobin in the intervention groups was **0.75 standard deviations higher** (0.41 to 1.08 higher)		3554<br>(16)	⊕⊕⊕⊝[1,2]<br>**moderate**	**SMD: 0.75 [95% CI: 0.41, 1.08]**
**Serum Ferritin Levels**	The mean ferritin in the intervention groups was **0.37 standard deviations higher** (0.13 to 0.62 higher)		2539<br>(9)	⊕⊕⊝⊝[1,2,3]<br>**low**	**SMD: 0.37 [0.13, 0.62**]
**Serum Zinc Levels**	The mean zinc in the intervention groups was **0.08 standard deviations higher** (0.02 lower to 0.19 higher		2821<br>(10)	⊕⊕⊕⊝[1,2]<br>**moderate**	**SMD: 0.08 [-0.02, 0.19]**
**Serum Retinol Levels**	The mean serum retinol in the intervention groups was **0.05 standard deviations lower** (0.23 lower to 0.13 higher		1927<br>(8)	⊕⊕⊝⊝[1,2,3]<br>**low**	**SMD: -0.05 [-0.23, 0.13]**

**Effect on Anemia**	Multiple Micronutrient fortification reduced risk of anemia by 45%	**RR: 0.55 [95% CI: 0.42, 0.71]**	4722 (13)	⊕⊕⊖⊖[1,2,3] **low**	
**Effect on Vitamin A deficiency**	Multiple Micronutrient fortification reduced risk of vitamin A deficiency by 10%	**RR: 0.90 [95% CI: 0.76, 1.06]**	2036 (6)	⊕⊕⊖⊖[1,2,3] **low**	**RR: 0.90 [95% CI: 0.76, 1.06]**
**Height-for-age Z Score**	The mean height-for-age z score in the intervention groups was **0.13 standard deviations higher** (0.04 lower to 0.29 higher)		2092 (8)	⊕⊕⊖⊖[1,2,3] **low**	**SMD: 0.13 [95% CI: -0.04, 0.29]**
**Weight-for-Age Z score**	The mean weight-for-age z score in the intervention groups was **0.12 standard deviations lower** (0.43 lower to 0.2 higher)		1988 (7)	⊕⊕⊖⊖[1,2,3] **low**	**SMD: -0.12 [95% CI: -0.43, 0.20]**
**Weight-for-height Z Score**	The mean weight-for-height z score in the intervention groups was **0.11 standard deviations lower** (0.4 lower to 0.17 higher)		2098 (8)	⊕⊕⊖⊖[1,2,3] **low**	**SMD: -0.11 [95% CI: -0.40, 0.17]**

CI: Confidence interval;

[1] Blinding of participants, personal and outcome assessment was not described or carried out in one or more of the studies
[2] Allocation concealment not carried out or discussed in one or more of the studies
[3] A large confidence interval noticed in the results that may account for imprecision in results
[4] An overall small sample size has been calculated
[5] A large unexplained/unaccounted drop out was observed in one of the studies
[6] A before-after study design was used

GRADE Working Group grades of evidence
High quality: Further research is very unlikely to change our confidence in the estimate of effect.
Moderate quality: Further research is likely to have an important impact on our confidence in the estimate of effect and may change the estimate.
Low quality: Further research is very likely to have an important impact on our confidence in the estimate of effect and is likely to change the estimate.
Very low quality: We are very uncertain about the estimate.

Table S2. Effect of food fortification with various micronutrients on health outcomes in women

Outcomes	Illustrative comparative risks* [95% CI]	Relative effect [95% CI]	No of Participants (studies)	Quality of the evidence (GRADE)	Comments
		**Iron Fortification**			
**Hemoglobin Levels**	The mean hemoglobin levels in the intervention groups was **0.62 standard deviations higher** (0.36 to 0.89 higher)		3223 (11)	⊕⊕⊕⊖[1,2] **moderate**	**SMD: 0.62 [95% CI: 0.36, 0.89]**
**Serum Ferritin Levels**	The mean serum ferritin in the intervention groups was **0.41 standard deviations higher** (0.23 to 0.60 higher)		3009 (10)	⊕⊕⊖⊖[1,2,4] **low**	**SMD: 0.41 [95% CI: 0.23, 0.60]**
**Effect on Anemia**	Iron fortification reduced the prevalence of anemia by 32%	**RR: 0.68 [95% CI: 0.49, 0.93]**	1316 (4)	⊕⊕⊖⊖[1,2,4] **low**	
		**Folate Fortification**			
**Neural Tube Defect**	Folate fortification reduced the incidence of neural tube defects by 33%	**RR: 0.57 [95% CI: 0.45, 0.73]**	62183 (8)	⊕⊕⊕⊖[6] **moderate**	
**Spina Bifida**	Folate fortification reduced the incidence of Spina Bifida by 36%	**RR: 0.64 [95% CI: 0.57, 0.71]**	41392 (11)	⊕⊕⊕⊖[6] **moderate**	
**Anencephaly**	Folate fortification reduced the incidence of anencephaly by 24%	**RR: 0.76 [95% CI: 0.68, 0.85]**	41261 (10)	⊕⊕⊕⊖[6] **moderate**	

**Serum Folate Levels**	The mean serum alkaline phosphatase levels in the intervention groups was **1.38 standard deviations higher** (0.20 lower to 2.95 higher)	1239<br>(3)	⊕⊕⊖⊖ [4,6]<br>**low**	**SMD: 1.38 [95% CI: -0.20, 2.95]**
**Iodine Fortification**				
**Urinary iodine concentrations**	The mean urinary iodine concentration in the intervention groups was 7**.16 standard deviations higher** (1.00 to 13.31 higher)	2352<br>(4)	⊕⊕⊕⊖[1,2]<br>**moderate**	**SMD: 7.16 [95% CI: 1.00, 13.31]**
**Calcium and Vitamin D fortification**				
**Serum PTH Levels**	The mean serum PTH levels of post-menopausal women in the intervention groups was **2.53 standard deviations lower** (4.42 to 0.65 lower)<br><br>The mean serum PTH levels of women of reproductive age in the intervention groups was **0.01 standard deviations lower** (0.32 to 0.30 lower)	398<br>(6)	⊕⊕⊖⊖[1,2,4]<br>**low**	**Post-menopausal women**<br>SMD: -2.53 [95% CI: -4.42, -0.65]<br><br>**Women of reproductive age**<br>SMD: -0.01 [95% CI: -0.32, -0.30]
**Serum Vitamin D Levels**	The mean serum vitamin D concentration of post-menopausal women in the intervention groups was 0.97 **standard deviations higher** (0.18 lower to 2.13 higher)<br><br>The mean serum vitamin D concentration of women of reproductive age in the intervention groups was 1**.10 standard deviations lower** (3.81 lower to 1.60 higher)	416<br>(5)	⊕⊕⊕⊖[1,2]<br>**moderate**	**Post-menopausal women**<br>SMD: 0.97 [95% CI: -0.18, 2.13]<br><br>**Women of reproductive age**<br>SMD: -1.10 [95% CI: -3.81, 1.60]
**Multiple Micronutrient Fortification**				

**Hemoglobin Levels**	The mean hemoglobin in the intervention groups was **0.31 standard deviations higher** (0.13 to 0.48 higher)		516 (1)	⊕⊕⊕⊖[1,2] **moderate**	**SMD: 0.31 [95% CI: 0.13, 0.48]**
**Serum Ferritin Levels**	The mean ferritin in the intervention groups was **0.47 standard deviations higher** (0.36 to 0.58 higher)		1214 (2)	⊕⊕⊕⊖[1,2] **moderate**	**SMD: 0.47 [95% CI: 0.36, 0.58]**
**Serum Zinc Levels**	The mean zinc in the intervention groups was **0.50 standard deviations higher** (0.38 to 0.61 higher		1214 (2)	⊕⊕⊕⊖[1,2] **moderate**	**SMD: 0.50 [95% CI: 0.38, 0.61]**
**Serum Retinol Levels**	The mean serum retinol in the intervention groups was **0.47 standard deviations lower** (0.30 to 0.65 higher		516 (1)	⊕⊕⊕⊖[1,2] **moderate**	**SMD: 0.47 [95% CI: 0.30, 0.65]**
**Effect on Anemia**	Multiple micronutrient fortification reduced the prevalence of anemia by 24%	**RR: 0.76 [95% CI: 0.48, 1.21]**	516 (1)	⊕⊕⊕⊖[1,2] **moderate**	

CI: Confidence interval;

[1] Blinding of participants, personal and outcome assessment was not described or carried out in one or more of the studies
[2] Allocation concealment not carried out or discussed in one or more of the studies
[3] A large confidence interval noticed in the results that may account for imprecision in results
[4] An overall small sample size has been calculated
[5] A large unexplained/unaccounted drop out was observed in one of the studies
[6] A before-after study design was used

GRADE Working Group grades of evidence
High quality: Further research is very unlikely to change our confidence in the estimate of effect.
Moderate quality: Further research is likely to have an important impact on our confidence in the estimate of effect and may change the estimate.
Low quality: Further research is very likely to have an important impact on our confidence in the estimate of effect and is likely to change the estimate.
Very low quality: We are very uncertain about the estimate.
